# Multi‐Stimuli‐Responsive Circularly Polarized Luminescence with Handedness Inversion and Near‐Infrared Phosphorescence in Chiral Metal‐Organic Framework Platform for White Light Emission and Information Encryption

**DOI:** 10.1002/advs.202502784

**Published:** 2025-03-17

**Authors:** Kun Zhang, Ni Dan, Ruo‐Yu Zhang, Jiaojiao Wei, Rui‐Xue Tian, Yongfan Zhang, Hong‐Ru Fu, Mei Qiu, Lu‐Fang Ma, Shuang‐Quan Zang

**Affiliations:** ^1^ College of Chemistry and Chemical Engineering Luoyang Normal University Luoyang 471934 China; ^2^ College of Materials and Chemical Engineering China Three Gorges University Yichang 443002 China; ^3^ College of Chemistry Fuzhou University Fuzhou 350116 China; ^4^ College of Chemistry and Materials Jiangxi Agricultural University Nanchang Jiangxi 330045 China; ^5^ College of Chemistry Zhengzhou University Zhengzhou 450001 China

**Keywords:** [2+2] cycloaddition, chiral metal‐organic framework, room temperature phosphorescence, stimuli‐responsive circularly polarized luminescence

## Abstract

Preparing multi‐color and multi‐stimuli‐responsive circularly polarized luminescence (CPL) materials and understanding the evolution of chirality through the visualized mode is still a challenge. Here, an encapsulation engineering approach of chiral metal‐organic frameworks (MOFs) is proposed to confine guest emitters to realize multi‐color and multi‐stimuli‐responsive CPL. Based on triplet‐triplet energy transfer (TTET), white CPL and near‐infrared circularly polarized room temperature phosphorescence (NIR‐CPRTP) can be obtained by introducing the pyrene derivatives. With the introduction of the guest containing vinylpyrene group, the light‐ and thermal‐responsive CPL with the signal inversion can be realized through the reversible [2+2] cycloaddition reaction between the ligand and guest triggered by visible light/ultraviolet light or heating. Furthermore, the excitation‐dependent CPL is successfully achieved with the incorporation of excited state intramolecular proton transfer (ESIPT) molecules into nanopores. Importantly, the chirality magnification can be greatly enhanced through the chiral spatial confinement, the accurate host‐guest single crystal structures of FLT@DCF‐12 and FLT@LCF‐12 provide the visualized mode to understand the mechanism of chirality transfer, amplification and responsiveness. White LED and multiple information display and encryption are further demonstrated. This breakthrough provides a new perspective to guest‐encapsulated chiral MOFs and contributes to the construction of stimuli‐responsive CPL‐active materials.

## Graphical abstract

Based on three strategies including Dexter energy transfer (DET), [2+2] cycloaddition and Excited intramolecular proton transfer (ESIPT), a universal platform is built by using chiral metal‐organic frameworks (MOFs) as host matrix to encapsulate guest emitters. NIR‐CPRTP, white CPL, and light‐, thermal‐ and excitation‐responsive CPL are achieved. The single crystal structures of FLT‐load MOFs provide a visualized mode to reveal the chirality transfer, amplification and response.

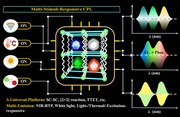

## 1 Introduction

Smart luminescent materials featuring multi‐stimuli responses to various stimuli, such as light,^[^ ^1^ ^]^ temperature,^[^ ^2^ ^]^ and chemical,^[^ ^3^ ^]^ have drawn considerable interest in advanced optoelectronic applications. Recently, there has been growing attention on the development of multi‐stimuli‐responsive circularly polarized luminescence (CPL) materials due to their high‐level applications in optical display,^[^ ^4^ ^]^ chemical sensing,^[^ ^5^ ^]^ and encryption.^[^ ^6^ ^]^ Up to now, a broad range of stimulus‐responsive chiroptical materials have been reported in the forms of liquid crystals,^[^ ^7^ ^]^ supramolecular polymers,^[^ ^8^ ^]^ and organic crystals.^[^ ^9^ ^]^ However, most of these materials mainly exhibit single‐mode fluorescence switching in CPL signals.^[^ ^10^ ^]^ The design of the chiroptical system allowing on‐demand multi‐mode and multi‐stimuli responsive luminescence switching in a universal platform remains challenging.

In recent years, room temperature phosphorescence (RTP) materials have been demonstrated to have potential applications in sensors,^[^ ^11^ ^]^ organic electronics,^[^ ^12^ ^]^ and encryption.^[^ ^13^ ^]^ Compared to circularly polarized fluorescence (CPF), circularly polarized phosphorescence (CPP) combining the natures of CPL and RTP has the unique advantages for photonic applications.^[^ ^14^ ^]^ Several kinds of CPP materials have been successfully prepared, such as polymer,^[^ ^15^ ^]^ supramolecular assemblies,^[^ ^16^ ^]^ heavy metal atom complexes.^[^ ^17^ ^]^ However, chiral materials are subject to the inherent limitation between the phosphorescence quantum yield (*Φ*_phos_) and the luminescence dissymmetry factor (*g*_lum_), it is difficult to maximize these two parameters simultaneously. Therefore, CPP‐active materials still face challenges such as limited variety and monotonous phosphorescence performance.^[^ ^18^ ^]^ The preparation of the multi‐stimuli‐responsive CPL systems with RTP is highly desirable.

Very recently, an increasing number of reports have focused on MOFs. Chiral MOFs, as a class of porous and crystalline medium, provide a powerful platform to promote the development of CPL‐active materials.^[^ ^19^ ^]^ Chiral MOFs owing to the long‐range periodic stacking, especially with helical channels, can improve the photoluminescence quantum yield (PLQY) and *g*_lum_ values.^[^ ^20^ ^]^ Chiral MOFs also can act as nanoporous containers to encapsulate achiral guest emitters, enabling the chirality of host to be better transmitted to guests, and the luminescence behaviors can be modulated through the introduction of different guest emitters,^[^ ^21^ ^]^ the multi‐color CPL and the amplification of *g*_lum_ can be achieved via the energy transfer.^[^ ^22^ ^]^ Importantly, the introduction of guest molecules into pores can largely restrict the vibration of the molecule and non‐radiative transitions, thus enhancing RTP.^[^ ^23^ ^]^ If the luminescent ligands of the host can bridge excitons from the singlet state of the guest emitter to its triplet state via TTET, the energy gap between the singlet and triplet energy could be remarkably reduced, also facilitating the generation of RTP.^[^ ^24^ ^]^ Our group has first reported MOFs with CPP.^[^ ^25^ ^]^ Liu's group has made systematical research on chiral induction and enlargement effect of some host‐guest MOFs with light‐responsive CPL.^[^ ^26^ ^]^ Despite all this, the guest‐encapsulated MOFs with stimuli‐responsive CPL were rarely.^[^ ^27^ ^]^ It is necessary to play the inherent potentials of MOFs to develop multi‐stimuli‐responsive CPL.

Excited intramolecular proton transfer (ESIPT) usually refers to a class of photochemical processes with photothermal and electrical‐induced Enol‐Keto tautomerization.^[^ ^28^ ^]^ This kind of luminescent molecule has a unique four‐level photocycle process, and usually has the characteristics of large Stokes shift, double emission and luminescence sensitivity to the environment.^[^ ^29^ ^]^ Therefore, CPL active materials with stimuli‐responsive luminescence behavior can be obtained by loading ESIPT molecules into chiral MOFs.

**Figure 1:**
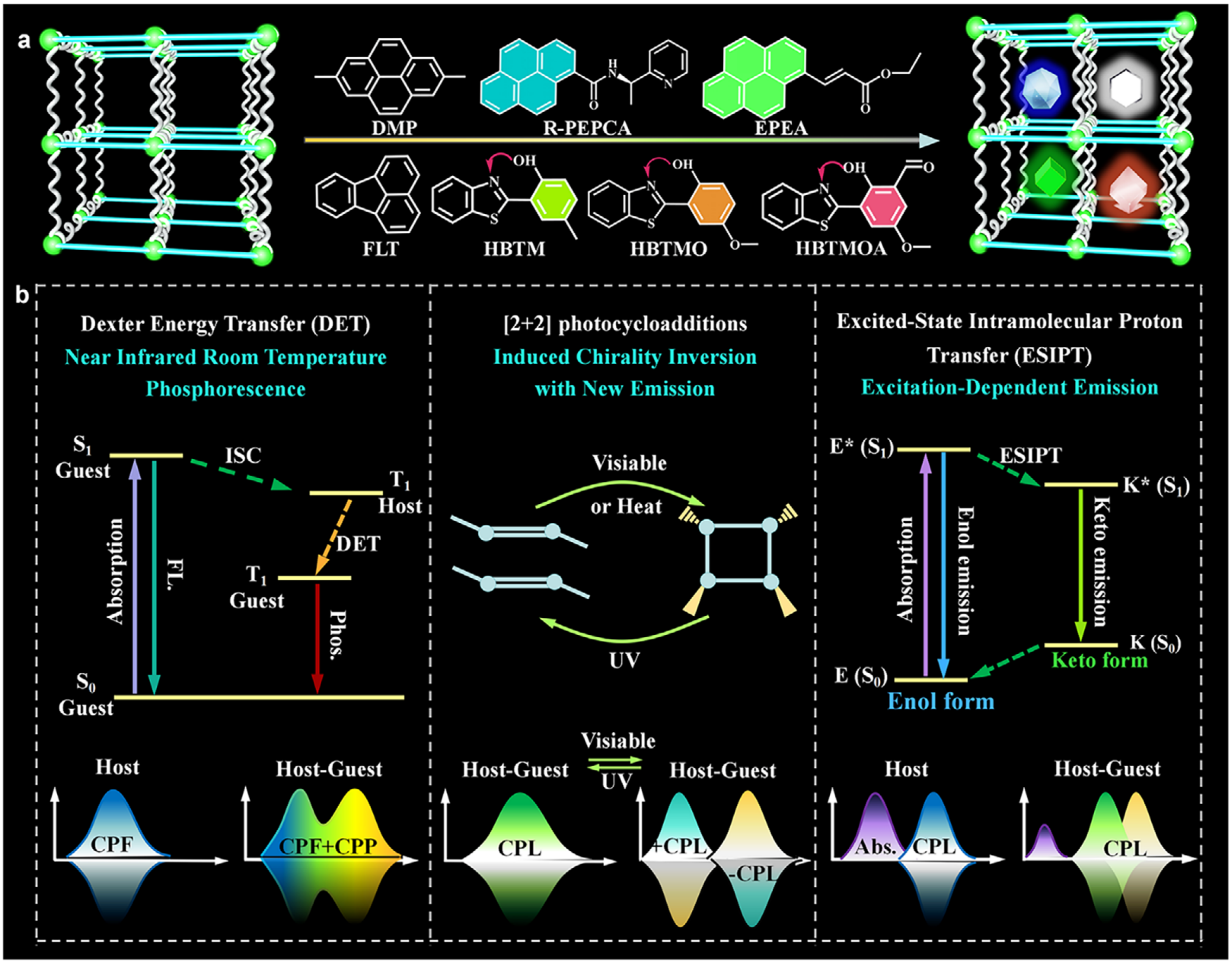
Schematic illustration of guest‐encapsulated chiral MOFs for the generation of dynamic CPL. a) Encapsulation of Guest Molecules into Chiral MOFs. b) Three Approaches for the Generation of Multi‐Color and Multi‐Stimuli‐Responsive CPL including DET, [2+2] cycloaddition and ESIPT.

Herein, we adopt three approaches including Dexter energy transfer (DET), [2+2] cycloaddition reaction and ESIPT to develop the multi‐mode and multi‐stimuli‐responsive CPL based on encapsulation engineering of chiral MOFs (**Figure** **1**). A pair of chiral MOFs (DCF‐12 and LCF‐12) were acted as host matrix, as well as eight molecules as guest emitters. First, with the introduction of pyrene‐based molecules (DMP and R/S‐PEPCA) into the confined pores. DMP@DCF‐12 and S‐PEPCA@DCF‐12 show the excitation‐dependent emission under the different excitation wavelengths, the near‐infrared RTP (NIR‐RTP) can be obtained owing to the stair‐stepping effect of DET. The *Φ*_Phos_ values of DMP@LCF‐12 and R‐PEPCA@LCF‐12 were calculated to be 87.73% and 78.51%, respectively. Importantly, the full‐color and white CPL (CIE: 0.34, 0.34) could be achieved in S‐PEPCA@DCF‐12 via fluorescence‐phosphorescence dual emission. Second, the light‐ and thermal‐responsive CPL with the inversion of signals can be smoothly modulated through [2+2] cycloaddition between guest (EPEA) and host framework. Third, with the incorporation of achiral guest emitters featuring ESIPT characteristics, these host‐guest composites exhibit the excitation‐dependent multi‐color CPL in a wide wavelength range. Importantly, the chirality evolution and the relationship of molecule stacking–the chirality of aggregates–chiroptical behaviors could be clearly revealed through the accurate single‐crystal structures of guest‐loaded MOFs. This work would be highly instructive for the design of multi‐color and stimuli‐responsive chiroptical materials via guest‐loaded MOFs.

## 2 Results and Discussion

### 2.1 The Encapsulation of Guests and Single Crystal Structure of Guest‐Loaded MOFs

Chiral MOFs as host matrix to encapsulate guests need to possess the following properties: high porosity, high stability, and easy preparation. Herein, DCF‐12 and LCF‐12 were utilized as host platforms.^[^ ^30^ ^]^ DCF‐12 crystallizes in the chiral orthorhombic *I*222 space group. DCF‐12 is a 3D pillar‐layer structure with 3D channels, TPE ligands exist in the formation of single chiral configuration. Two types of 1D helical channels own the size of 9.1 × 10.0 Å^2^ and 7.2 × 12.4 Å^2^, respectively (Figure S1, Supporting Information). The accessible void is estimated to be 45.0% of the total volume. The TG curve confirms that DCF‐12 can maintain the stability of the framework up to 350 °C, thereby, DCF‐12 and LCF‐12 are suitable for being as containers.

First, considering that the channels are straight, the rigid and planar molecules fluoranthene (FLT) and 2,7‐dimethylpyrene (DMP) were employed as guest emitters. The in‐situ encapsulation approach was used to construct chiral host‐guest adducts.

**Figure 2:**
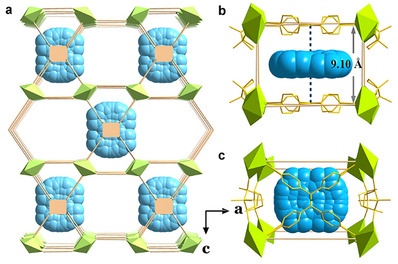
Single‐crystal structure of host‐guest composite FLT@DCF‐12. a) Single‐crystal structure of FLT@DCF‐12. b) Sandwich stacking of TPE and FLT, the distance is ≈4.55 Å between FLT and TPE. c) Sandwich stacking of TPE and FLT from the top view.

The crystal color of DCF‐12 is nearly unchanged with the incorporation of FLT. Generally, all FLT@DCF‐12 crystals have the high crystallinity, the morphology of FLT@DCF‐12 is in accordance with that of the parental MOFs, as well as the greatly matched PXRD (Figure S3a, Supporting Information). Significantly, FLT‐encapsulated DCF‐12 is suitable for X‐ray single crystal diffraction. We successfully obtained the single‐crystal structures of FLT@DCF‐12 and FLT@LCF‐12, the crystallographic analyses revealed that the host‐guest structures are similar to those of the pristine DCF‐12 and LCF‐12. Through the visualized structures, it can be found that not every pore was filled with FLT molecule, the encapsulation of FLT molecules exhibits the regular interval insertion, and these guest species were confined into the smaller pores rather than the biggest pores (**Figure** **2**a). Each guest molecule was immobilized between two TPE linkers to form a sandwich structure (Figure 2b). The perpendicular distance of the neighboring FLT molecules is ≈19.16 Å (Figure S2, Supporting Information), and the nearest distance between FLT molecule and TPE molecule is 4.55 Å, to a great extent, such distance between FLT and TPE means the formation of exciplex due to the parallel‐parallel stacking. The pore built of chiral D‐cam and *M*‐type TPE molecules present a chiral space, as FLT enters the chiral cavity and becomes part of these pores, FLT can be endowed with chirality via the chiral space transfer. The structural formula [Zn_2_(D‐cam)_2_(TPE)(FLT)_0.5_] from the single‐crystal structure of FLT@DCF‐12 can be given, the molar ratio of TPE/FLT is 2. In order to validate whether the residual pores can continue to accommodate guest molecules, the larger quantity of FLT was used. Nevertheless, when the stoichiometry of TPE/FLT is 1 and 1.5 in the raw materials, the molar ratio of TPE/FLT is still 2 in host‐guest systems. These results provide a basis for the host‐guest construction by using other guests.

FLT@DCF‐12 shows intense blue emission with Commission International de l'Eclairage (CIE) coordinates of (0.15, 0.17), the fluorescence quantum yield (*Φ*_FL_) of FLT@DCF‐12 is up to 59.42%, which is much higher than that of the FLT powder (27.62%) under excitation at 398 nm.

### 2.2 Heat‐Responsive Near‐Infrared Phosphorescence via TTET

Upon the loading of DMP, DMP@DCF‐12 displays yellow emission, rather than blue emission of DMP in DMA (Figure S10, Supporting Information), the emission spectra of DMP@DCF‐12 appeared nearly 160 nm red shift toward that of the DMP solution. This can be attributed to energy transfer from DCF‐12 to DMP due to the overlap between the absorption of DMP and the emission of DCF‐12 (Figures S20 and S18a, Supporting Information). Besides, the fluorescence lifetime of DMP@DCF‐12 is 43.74 ns at 560 nm (Figure S11a, Supporting Information), and the fluorescence lifetime of DCF‐12 and DMP is 7.26 and 5.49 ns at 505 and 610 nm, respectively (Figures S10d and S18b, Supporting Information). It can be observed that the fluorescence lifetime of DMP@DCF‐12 significantly becomes longer with the introduction of DMP compared to that of DCF‐12 itself and DMP. Meanwhile, considering the parallel stacking among DMP and organic linker TPE, the main peak of DMP@DCF‐12 with a red‐shift of 160 nm may be also attributed to the generation of exciplex between TPE and DMP. Interestingly, the red afterglow of DMP@DCF‐12 lasting ≈2.5 s can be observed by the naked eye at room temperature (Figure S31a, Supporting Information). When the photoluminescence (PL) spectra were measured by microsecond light, it is found that the new emission band ranging from 570 to 750 nm appears compared to the fluorescence spectra (Figure S27a, Supporting Information). By using a time‐gated approach with a delayed time of 0.5 ms, the red emission bands do not disappear, and the lifetimes at 590 and 650 nm are up to 178.83 and 186.55 ms excited at 380 nm at ambient conditions (Figure S27c, Supporting Information), these results indicate that the long‐lived emissions in the NIR region maybe belong to phosphorescence. Furthermore, the temperature‐dependent delayed emission was measured from 390 to 77 K, the overall emission gradually increased (Figure S26f, Supporting Information), which is in consist with the inhibitions of the non‐radiative transition at low temperature.^[^ ^31^ ^]^ Taking together the above results, it can be concluded that the afterglow behaviors in the longer wavelength are phosphorescence. The *Φ*_Phos_ of DMP@DCF‐12 is ≈87.73% (**Table** **1**; Figures S45–S47, Supporting Information). Importantly, under the varied excitation wavelengths from 300 to 380 nm, it can obtained that only the intensities of PL spectra changed without the shift of peak positions, once the excitation wavelengths in the visible region were adopted, the whole emission peaks gradually red‐shifted along with the maximum peak from 560 to 650 nm, showing the excitation‐dependent property (Figure S26b,c, Supporting Information), the CIE coordinates clearly shows the color of the PL spectra turned from green to orange to deep red (Figure S26d, Supporting Information).

**Table 1:** Summary of photophysical properties of guest‐encapsulated MOF_S_ at ambient temperature.

Compound^a^ ^)^	λ_FL_[nm]	λ_Phos_[nm]	τ_FL_ [ns]	τ_Phos_ [ms]	*Φ*_FL_ [%]	*Φ*_Phos_ [%]	S_1_ [Guest]	T_1_ [Guest]
FLT@DCF‐12	466	‒	49.56	‒	59.42	‒	2.66	1.61
DMP@DCF‐12	560	590/650	43.75	178.73/186.55	22.19	87.73	3.14	1.35
S‐PEPCA@DCF‐12	510	600/650	87.64	146.93/149.94	25.58	76.43	3.16	1.32
R‐PEPCA@DCF‐12	510	600/650	86.93	146.72/149.10	25.44	75.85	3.16	1.32
EPEA@DCF‐12	535	‒	80.40	‒	31.20	‒	2.52	1.05
HBTM@DCF‐12	425/465/520	‒	3.06/2.87/2.48	‒	5.90	‒	3.02	1.98
HBTMO@DCF‐12	435/470	‒	3.27/4.53	‒	4.21	‒	2.87	1.86
HBTMOA@DCF‐12	437/495/595	‒	1.73/1.79/3.97	‒	7.31	‒	2.71	1.82

^a)^ λ_FL_, main fluorescence peak; λ_Phos_, main phosphorescence peak; τ_FL_, fluorescence lifetime; τ_Phos_, phosphorescence lifetime; *Φ*_FL_, fluorescence quantum yield; *Φ*_Phos_, phosphorescence quantum yield; S_1_, the value of singlet state of guest molecule; T_1_, the value of triplet state of guest molecule.

Given the generation of the phosphorescence property with the encapsulation of pyrene‐based molecule, and compared to the rigid and planar achiral molecules mentioned above, the other pyrene‐cored molecules (R/S‐PEPCA and EPEA) were used.

**Figure 3:**
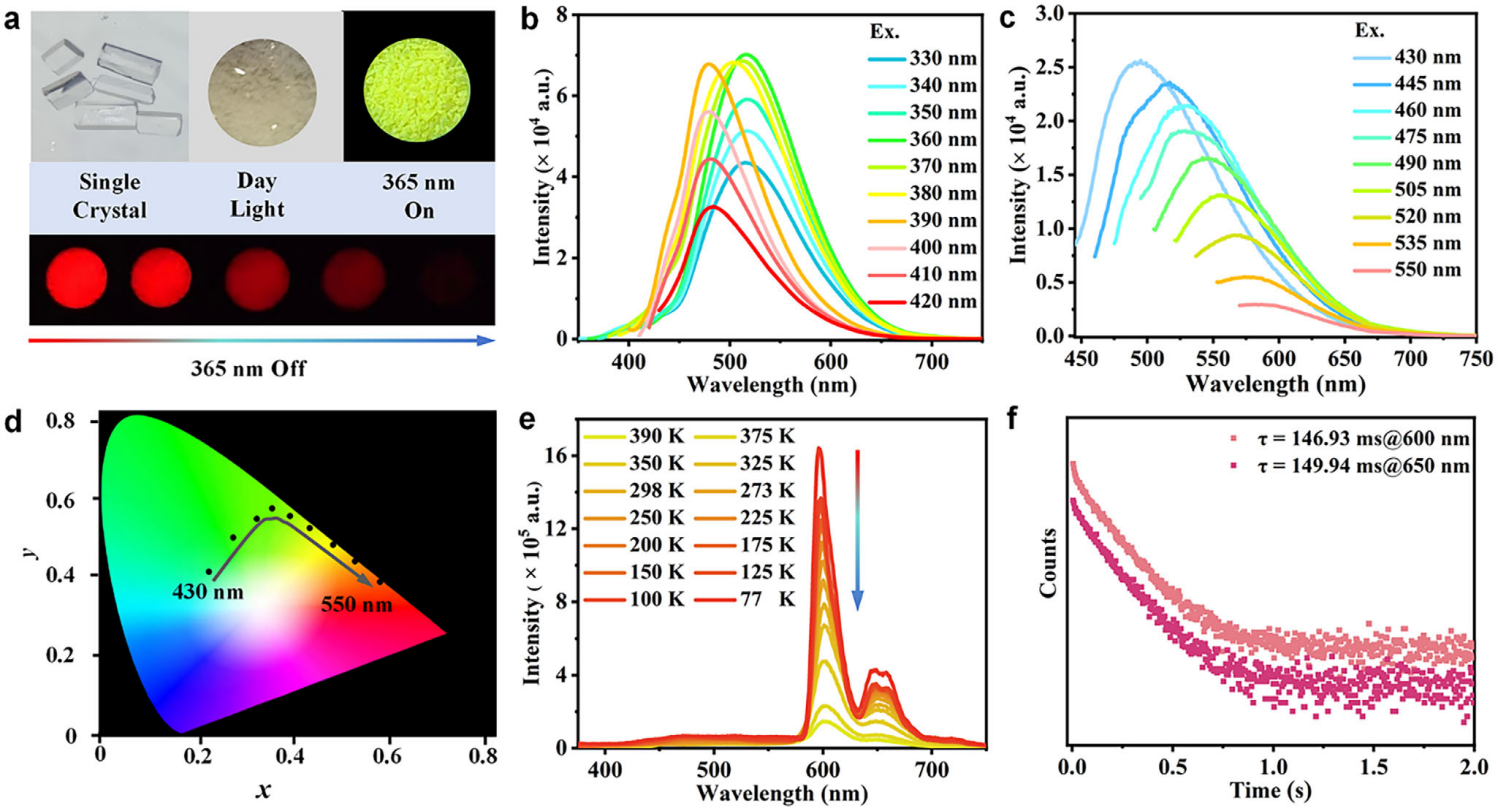
PL properties of S‐PCEA@DCF‐12. a) Photographs of S‐PCEA@DCF‐12 under visible light, 365 nm and after turning off UV light. b) Emission spectra of S‐PCEA@DCF‐12 under different excitation wavelengths from 330 to 420 nm. c) Emission spectra of S‐PCEA@DCF‐12 under different excitation wavelengths from 430 to 550 nm. d) CIE coordinate diagram of the PL spectra of S‐PCEA@DCF‐12 (Ex. from 430 to 550 nm). e) Temperature‐dependent phosphorescence spectra of S‐PCEA@DCF‐12 with a delayed time of 0.5 ms. f) Phosphorescence lifetimes at 600 and 650 nm under ambient conditions.

The 3D size of S‐PEPCA is ≈7.7 × 9.6 × 15.5 Å^3^, respectively, which is smaller than the scale of pores, it can be introduced into DCF‐12. The prompt spectra of S‐PEPCA in solid state shows blue fluorescence (Figure S12, Supporting Information), while S‐PEPCA@DCF‐12 shows the green fluorescence emission, the time‐resolved fluorescence decay measurements showed that the lifetime of S‐PEPCA@DCF‐12 increased from 7.16 to 87.64 ns compared to that of S‐PEPCA power (Figures S12e and S32a, Supporting Information), indicating that energy transfer occurs between S‐PEPCA and TPE.^[^ ^32^ ^]^ S‐PEPCA@DCF‐12 also exhibits the excitation‐dependent photoluminescence under the irradiation in the range from 430 to 550 nm (**Figure** **3**c). As shown in the CIE coordinate diagram, the color variation presents approximately a linear trend like that of DMP@DCF‐12 (Figure 3d). Intriguingly, S‐PEPCA@DCF‐12 also exhibits the remarkable red afterglow under ambient conditions, the temperature‐dependent delayed emission was measured with a delayed time of 0.5 ms, the emission intensity between 580 and 690 nm gradually enhanced with the decreasing temperatures (Figure 3e), and the emission bands are almost identical to the delayed spectra of S‐PEPCA, indicating the phosphorescence emission originates from S‐PEPCA (Figure S12, Supporting Information). The time‐resolved delayed spectra shows that the lifetimes at 600 nm and 650 nm are 146.93 and 149.94 ms, respectively (Figure 3f). The *Φ*_Phos_ of S‐PEPCA@DCF‐12 is 76.43%. In contrast, no afterglow can be observed from S‐PEPCA at ambient temperature. These results confirm that the nonradiative decay process of S‐PEPCA can be greatly restricted in the confined pores, and the phosphorescence intensity and lifetimes can be dramatically enhanced.^[^ ^33^ ^]^ What's more, the PL spectra of S‐PEPCA@DCF‐12 exhibit white‐light emission with multiple emission peaks at 437, 470, 525, 602, and 650 nm, covering the whole visible region upon excitation at 310 nm (Figure S33a, Supporting Information), which benefits from the fluorescence and phosphorescence emission, corresponding to CIE coordinates of (0.34, 0.34), which is close to the white‐light emission standard (Figure S33b, Supporting Information).

### 2.3 Light‐ and Thermal‐Responsive Fluorescence via Photocycloaddition

**Figure 4:**
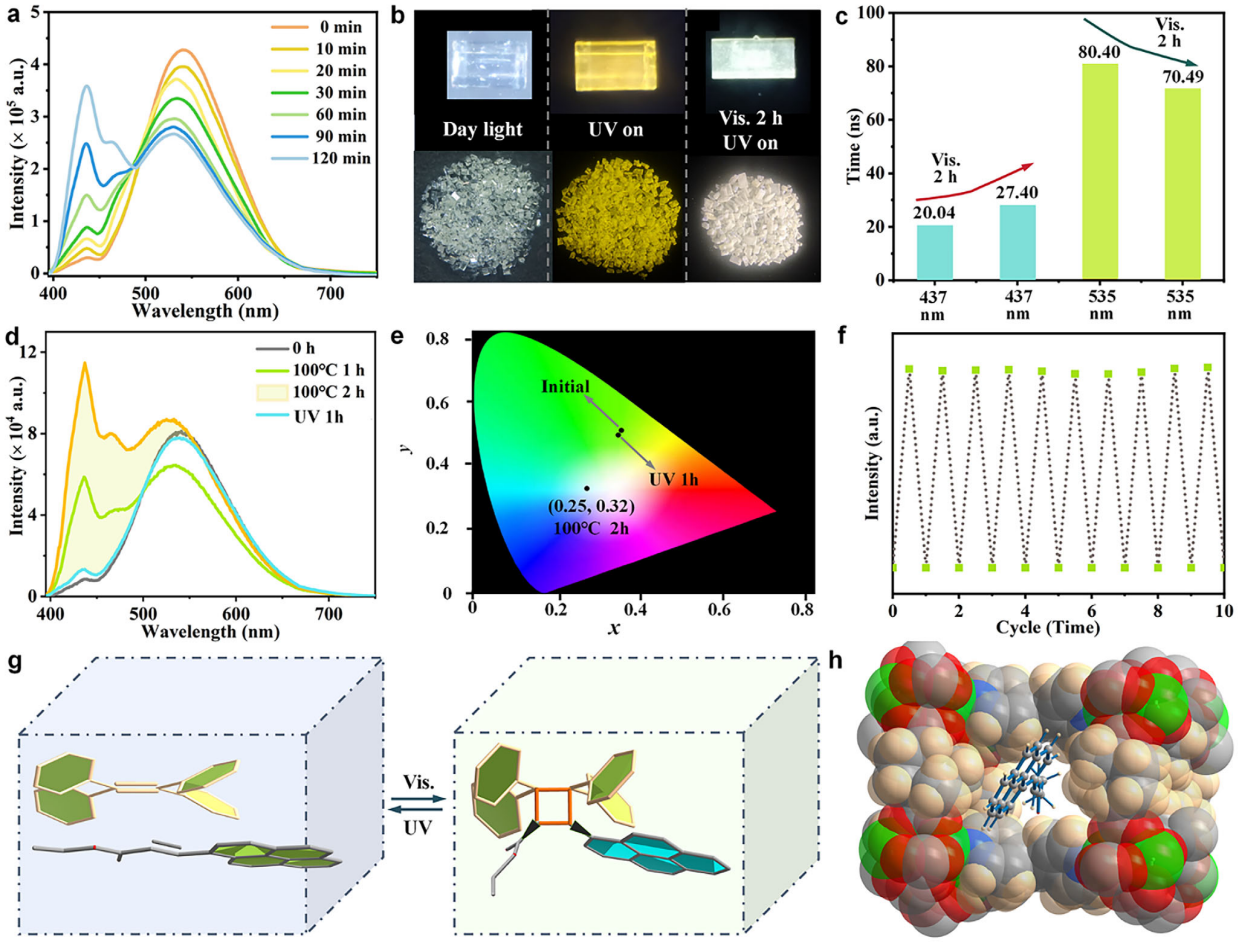
PL properties and light‐ and thermal‐responsive mechanism of EPEA@DCF‐12. a) Emission of EPEA@DCF‐12 upon visible light irradiation under different times. b) Photographs for EPEA@DCF‐12 before and after UV irradiation and under visible light irradiation for 2 h. c) The change of fluorescence lifetimes before and after cycloaddition at 437 and 535 nm. d) The reversible cycloaddition reaction can be achieved through heating and UV irradiation. e) CIE coordinates of EPEA@DCF‐12 under initial state, being heated at 100 °C for 2 h and restored state. f) Fluorescence intensity at 437 nm of EPEA@DCF‐12 after alternating exposure to heating and UV light. g) The structure switching of TPE and EPEA. h) The simulated host‐guest structure of EPEA@DCF‐12.

EPEA‐encapsulated DCF‐12 crystals are transparent, and show a bright yellow emission upon exciting at 365 nm (**Figure** **4**b). The DMA solution of EPEA displays a blue emission at 437 and 467 nm under excitation at 365 nm, as well as green emission at 504 nm of EPEA powder (Figure S14, Supporting Information). Instead, the emission band of EPEA in the confined channels shifted to the longer wavelength. The same reasons identical to that of DMP@DCF‐12 could be affirmed. The CIE coordinates of EPEA@DCF‐12 are (0.35, 0.52). The *Φ*_FL_ of EPEA@DCF‐12 was measured to be 31.20%, much higher than 1.47% of DCF‐12. Unlike the above pyrene derivatives‐embedded DCF‐12, no phosphorescence emission can be detected in EPEA@DCF‐12. Surprisingly, when EPEA@DCF12 crystals are exposed to visible light, the fluorescence peak at 437 nm was significantly enhanced, the peak at 535 nm gradually decreased with the time from 0 to 2 h, the crystals emit near white light (Figure 4a,b), and the fluorescence peak at 437 nm was similar to the emission of EPEA in DMA (10^−5^ M), largely indicating that this fluorescence peak comes from the monomer emission of EPEA molecules. The reversible switching of the fluorescence emission at 437 and 535 nm can be regulated by the visible and UV stimuli. We analyzed that the reversible switching of the emission may be attributed to the [2+2] cycloaddition reaction between TPE and EPEA molecules. The reasons are as follows: first, the distance of the adjacent parallel TPE molecules is approximately 10 Å, so the center‐to‐center distance of C = C groups between TPE and EPEA is close to 5 Å, such distance is highly suitable for cycloaddition.^[^ ^34^ ^]^ Secondly, all the C = C skeletons are restricted intensively in the order framework and confined channels, which is beneficial for cycloaddition.^[^ ^35^ ^]^ Importantly, from the practical monitor by high‐resolution mass spectrometry, the actual molecular weight (636.76) of the cycloaddition product is nearly equal to the theoretical value (636.22), unambiguously confirming the existence of the cycloaddition reaction of EPEA and TPE (Figure S37, Supporting Information). Accompanied by the occurrence of reactions, the face‐to‐face stacking between EPEA and TPE is twisted, and the π‐π conjugations are destroyed, the pyrene moieties of EPEA molecules present the single molecule state, leading to the generation of the monomer emission of pyrene at 437 nm. In addition, the fluorescence lifetime at 437 and 535 nm was tested after visible irradiation for 2 h, the lifetime at 535 nm was shortened from 80.40 ns to 70.49 ns, and the lifetime at 437 nm increased, illustrating that the falling‐off of exciplex emission and occurrence of cycloaddition reaction (Figure 4c).

Interestingly, we found that the reversible switching of the cycloaddition reaction could be also finished through the heating processes. When EPEA@DCF12 crystals were heated at 100 °C for 2 h, the emission at 437 nm gradually enhanced with a continuous luminescence switching behavior (Figure 4d), the fluorescence color turned from yellow green to white with the change of CIE from (0.34, 0.51) to (0.25, 0.32) (Figure 4e). The heat‐activated crystals can be restored the initial state by UV irradiation. The photocoupling and ‐decoupling for EPEA@DCF12 can be cycled at least ten times (Figure 4f). To the best of our knowledge, the ring opening and closing transformation of [2+2] cycloaddition reaction triggered by light and thermal media simultaneously is rare.^[^ ^36^ ^]^ Moreover, theoretical simulation was carried out to figure out the existence forms of EPEA into host framework. It revealed that EPEA molecule is almost suspended in the confined channel via intermolecular force, the location of C = C bond of EPEA is relatively close to the center of TPE, the distances among the centers of the C = C bond of EPEA and two TPE were calculated to be 4.85 Å and 4.91 Å, such short distance is very favorable to the cycloaddition reaction in spatial structure (Figure 4e; Figure S36, Supporting Information), which is in accordance with the experimental results.

### 2.4 Excitation‐Dependent Fluorescence via ESIPT

Next, a series of ESIPT molecules were designed as guests. ESIPT molecules, which own the tautomeric transformation between the excited Enol form and the excited Keto form, can realize the transformable emissions.^[^ ^37^ ^]^ 2‐(2′‐hydroxyphenyl)benzothiazole (HBT) molecule is a typical ESIPT chromophore.^[^ ^38^ ^]^ Here, three HBT‐based small molecules (HBTM, HBTMO, HBTMOA) were prepared. The photophysical properties of these HBT derivatives were investigated (Figures S15−S17, Supporting Information), the similar optical characteristics were obtained, which are consistent with the previous reports.^[^ ^39^ ^]^

**Figure 5:**
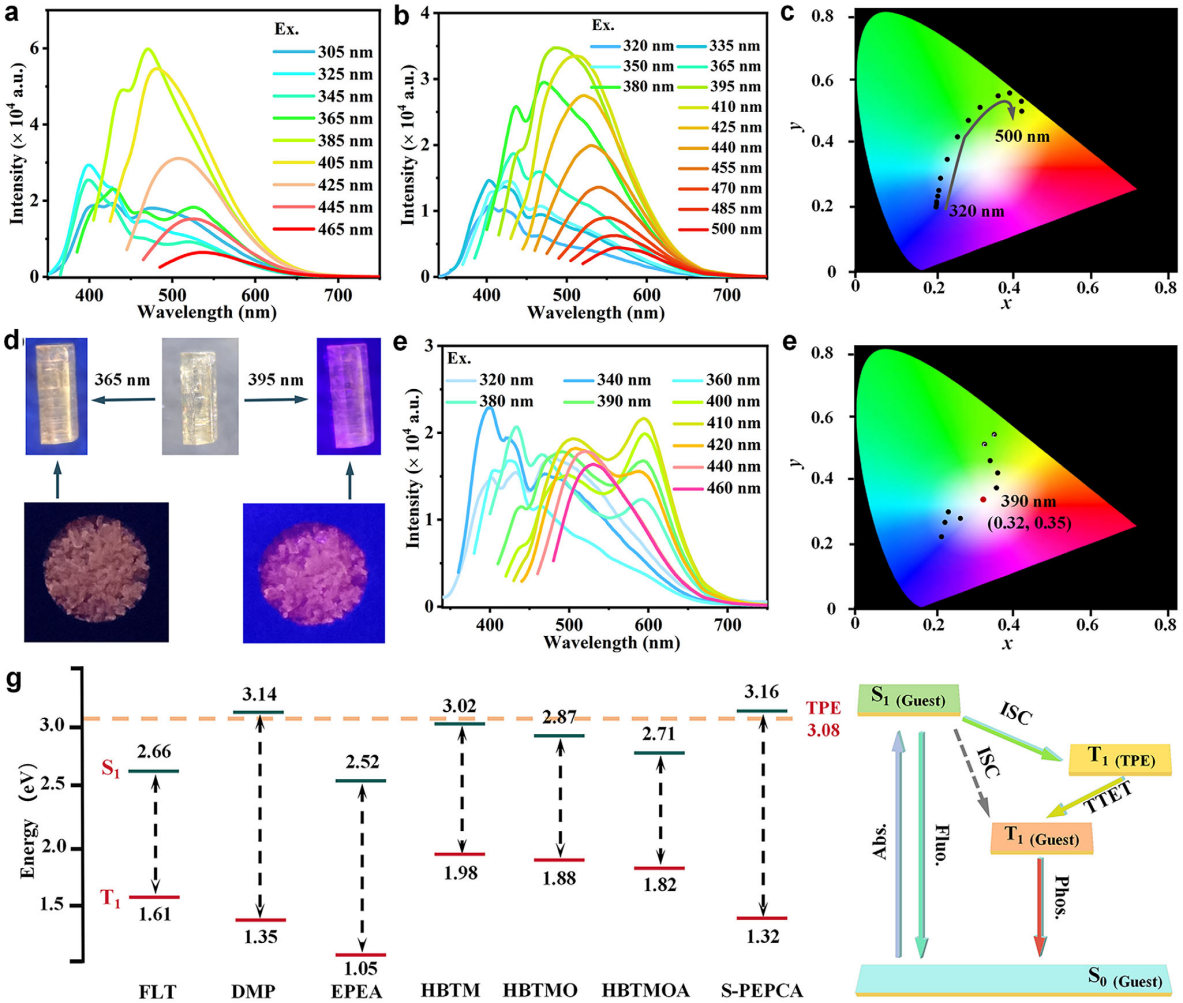
PL properties of HBTM@DCF‐12, HBTMO@DCF‐12 and HBTMOA@DCF‐12, and energy transfer mechanism for the generation of RTP. a) Excitation‐dependent fluorescence emission of HBTM@DCF‐12. b) Excitation‐dependent fluorescence emission of HBTMO@DCF‐12. c) CIE coordinate diagram of HBTMO@DCF‐12. d) Photographs of HBTMOA@DCF‐12 under 365 and 395 nm. e) Excitation‐dependent fluorescence emission of HBTMOA@DCF‐12. f) CIE coordinate diagram of HBTMOA@DCF‐12. g) Energy levels of guest and ligand molecules (green: S_1_, red: T_1_).

The solid‐state emission spectra of HBTM@DCF‐12 were first investigated at room temperature. As expected, HBTM@DCF‐12 shows a broad PL band with two peaks centered at 430 and 475 nm when excited at 305 nm (**Figure** **5**a). Upon changing the excitation wavelengths from 325 to 365 nm, the peak located at longer wavelengths become more apparent. Then, the PL bands only display one emission peak with a significant red shift under excitation wavelengths above 385 nm, and the red‐shifted emission gradually decreases. The CIE coordinate diagram shows the obvious color change (Figure S41a, Supporting Information). For HBTM crystal powder, only one peak was observed at 523 nm under the excitation wavelengths ranging from 270 to 410 nm, no excitation‐wavelength‐dependent fluorescence was observed for

HBTM itself (Figure S15d, Supporting Information). Compared to the solid‐state photophysical properties, the dilute solution of HBTM in DMA (10^−5^ M) exhibited the strong fluorescence emission with one peak at 400 nm, and weak fluorescence with dual peaks at ≈400 and 480 nm in DMA (10^−3^ M), and the intensity gradually enhanced with the increasing HBTM concentration (Figure S15a, Supporting Information), indicating that the proper dispersion favors the generation of ESIPT processes.^[^ ^40^ ^]^ Thereby, these results demonstrated that DCF‐12 is like a porous solid solvent, the confinement and separation of HBTM molecules into its pores can efficiently improve ESIPT property. Additionally, the time‐resolved spectra showed that the lifetime of the fluorescence emission, which was excited under irradiation over 385 nm, was longer than that of one under excitation less than 385 nm, the lifetime changed from 2.93 to 4.39 ns (Figure S42 and Table S1, Supporting Information). Through comprehensive consideration of the host‐guest stacking, the ESIPT feature, it is enormously certain that the long wavelength‐excited emission results from the exciplex formation consisting of HBTM and TPE, and this state is beneficial for the generation of excitation wavelength‐dependent emission.^[^ ^41^ ^]^

Similar processes for HBTMO and HBTMOA were carried out. HBTMO@DCF‐12 exhibits the multi‐emission at 403, 430, and 470 nm with the intensity change without the wavelength shift, when the excitation wavelength is below 380 nm, while the remarkable excitation‐dependent emission can be realized under the excitation wavelengths ranging from 395 to 500 nm, the CIE coordinate variation exhibits almost linearly from (0.21, 0.23) to (0.47, 0.52), and with the color of change from the blue to green to yellow (Figure 5b,c).

As for HBTMOA@DCF‐12, the emission color is pink and purplish red following excitation at 365 and 395 nm, respectively (Figure 5d). The emission behaviors present colorful variations. The emission bands gradually become wide under the excitation from 320 to 380 nm. When the excitation wavelengths are tuned from 390 to 420 nm, a new emission peak at 595 nm appears, HBTMOA@DCF‐12 exhibits dual emission with two peaks at ≈500 and 595 nm. Whereas, with the continued increase of excitation wavelengths, the emission bands only show a single peak accompanied by a red shift (Figure 5e). Impressively, the white‐light emission can be realized upon the excitation wavelength at 390 nm with the CIE coordinates of (0.32, 0.35) (Figure 5f).

It should be noted that as for the guests except FLT, although we can only obtain the single crystal structures of the host framework without guest molecules, it reveals that all molecules maintain the chiral skeleton without the generation of chiral inversion, and the space group is as same as that of DCF‐12. These results can guarantee the correctness of the below chiroptical characterization.

### 2.5 Theoretical Calculations for Luminescence Properties

Aiming at thoroughly figure out the luminescence mechanism of host‐guest systems, the charge and energy transfer was evaluated. TD‐DFT calculations on the highest occupied molecular orbitals (HOMO), the lowest unoccupied molecular orbitals (LUMO), and the energy levels of singlet and triplet states of all guests and linkers were carried out (Figures S48−S51, Supporting Information). It can find that the charge transfer only can occur between TPE and HBTM and HBTMO, resulting in these guests‐loaded hosts with relatively low *Φ*_FL_. Besides, it can confirm that the phosphorescence generation of DMP@CMOFs and R/S‐PEPCA@CMOFs is without charge transfer effect. The energy level ranges of the lowest singlet state (S_1_) and lowest triplet state (T_1_) of eight guest molecules are 2.52–3.16 and 1.05–1.98 eV, respectively (Figure 5g). The band gaps (ΔE_ST_) between S_1_ and T_1_ of eight guest molecules were in the range of 0.89–1.84 eV. Such large ΔE_ST_ values do obviously not facilitate intersystem crossing (ISC) of the excitons, no phosphorescence can be observed in these guest powders at ambient temperature. For host‐guest structures, the new energy transfer steps are built between S_1_ and T_1_ of pyrene derivatives. The T_1_ state (3.08 eV) of TPE lying between the S_1_ states and T_1_ states of DMP and R/S‐PEPCA can act as a bridge for the energy transfer of the guests, the energy transfer can occur from S_1_ of DMP and R/S‐PEPCA to T_1_ of TPE and to T_1_ of DMP and R/S‐PEPCA, resulting in the reduction of ΔE_ST_. The ΔE_ST_ between the DMP and TPE are narrowed to 0.06 eV, as well as 0.08 eV for S‐PEPCA and the hosts. The reduced energy gaps are beneficial for ISC process.^[^ ^42^ ^]^ Therefore, when the guest‐doped composites are excited at different excitation wavelengths, the exciton transition between guest molecules and TPE can undergo different passages to generate the distinct phosphorescence behaviors.^[^ ^43^ ^]^

### 2.6 Stimuli‐Responsive Chiroptical Properties

The chiroptical properties of these host‐guest adducts are characterized under ambient conditions. First, circular dichroism (CD) spectra were measured. DCF‐12 and LCF‐12 show a negative and a positive solid‐state CD signal at 246 and 325 nm with KBr pellets, respectively. A negative and positive Cotton effect of FLT@DCF‐12 and FLT@LCF‐12 was observed at 260 and 315 nm corresponding to the absorption bands of these composites (Figure S25, Supporting Information). For DMP@DCF‐12 and DMP@LCF‐12, the negative and positive CD signals with two new peaks ≈280 and 355 nm are obtained, which was mainly attributed to the π‐π* transitions of guests and TPE (Figure S30, Supporting Information).

S‐PEPCA has two absorption peaks at 330 nm and 343 nm in 1 × 10 M^−5^ DMA solutions (Figure S12, Supporting Information). S‐PEPCA and R‐PEPCA present mirror‐like CD spectra in DMA solutions (10^−5^ M), and S‐PEPCA powder has a negative CD signal at 275 nm and a positive signal at 350 nm, respectively (Figure S13a, Supporting Information). When S‐PEPCA and R‐PEPCA are packaged into DCF‐12, the CD spectra of S‐PEPCA@DCF‐12 and R‐PEPCA@DCF‐12 exhibit nearly the same CD signals with the same signal directions in the wavelength range of 220−400 nm. Accordingly, S‐PEPCA@LCF‐12 and R‐PEPCA@LCF‐12 exhibit excellent opposite mirrored CD signals to S‐PEPCA@DCF‐12 and R‐PEPCA@DCF‐12 (Figure S32i, Supporting Information). These results are quite distinct from those of R/S‐PEPCA in the solid‐state or solution, suggesting the chirality behaviors depending on the global structure of host‐guest systems.^[^ ^44^ ^]^ The fresh EPEA@DCF‐12 and EPEA@LCF‐12 exhibit the nearly mirror‐image CD signals with main peak located ≈220 and 247 nm, while EPEA@DCF‐12 and EPEA@LCF‐12 show a positive and negative CD signal at 220 nm after visible irradiation, respectively (Figures S21 and S39, Supporting Information).

HBTM@DCF‐12 and HBTM@LCF‐12 exhibited the CD characteristics with the mirror‐image peaks at 255 and 300 nm, respectively (Figures S22 and S41d, Supporting Information). HBTMO@DCF‐12 and HBTMO@LCF‐12 and HBTMOA@DCF‐12 and HBTMOA@LCF‐12 given the similarity of CD spectra (Figures S23, S24, S43, and S44, Supporting Information). The excellent results of CD spectra of these guest‐encapsulated MOFs indicate that these chiral host‐guest complexes are enantiomeric, and the guests inside the pores were endowed with chirality property through the chirality transfer of chiral space and chiral lattice.

The diversified luminescence and CD behaviors prompted us to further investigate their excited states by CPL spectra. The CPL signals of DCF‐12 and LCF‐12 cannot be detected due to the weak luminescence. In contrast, the mirror‐symmetric CPL spectra of FLT@DCF‐12 and FLT@LCF‐12 were obtained, showing intense emission centered at 470 nm (Figure S25e, Supporting Information), and matching the emission of FLT chromophore (λ_em_ = 465 nm). The magnitude of induced CPL was evaluated by *g*_lum_ around the maximum emission wavelength, affording the values of ‒0.97 × 10^‒2^ and +1.02 × 10^‒2^ for FLT@DCF‐12 and FLT@LCF‐12, respectively (Figure S25f, Supporting Information). Where *g*_lum_ = 2 × (*I*_L_‒*I*_R_)/(*I*_L_+*I*_R_), *I*_L_ and *I*_R_ are the luminescence intensities of left and right circularly polarized light, respectively.^[^ ^45^ ^]^ DMP@DCF‐12 and DMP@LCF‐12 exhibit mirror‐image CPF spectra centered at 555 nm, but also show the CPP characteristic, when these crystal samples were treated under 120 °C for 5 h (Figure S31b,c, Supporting Information). Interestingly, the apparent CPP signals can be detected, when these crystal samples were dropped by BzOH after 0.1 hours, the main peaks centered at 650 nm (Figure S31d,e, Supporting Information). Importantly, In order to evaluate the repeatability, the luminescent properties containing the phosphorescence intensity and phosphorescence lifetime between the wet and dry state almost keep stable after 8 cycles (Figure S31f,g, Supporting Information), indicating the excellent repeatability of CPL responsiveness.

**Figure 6:**
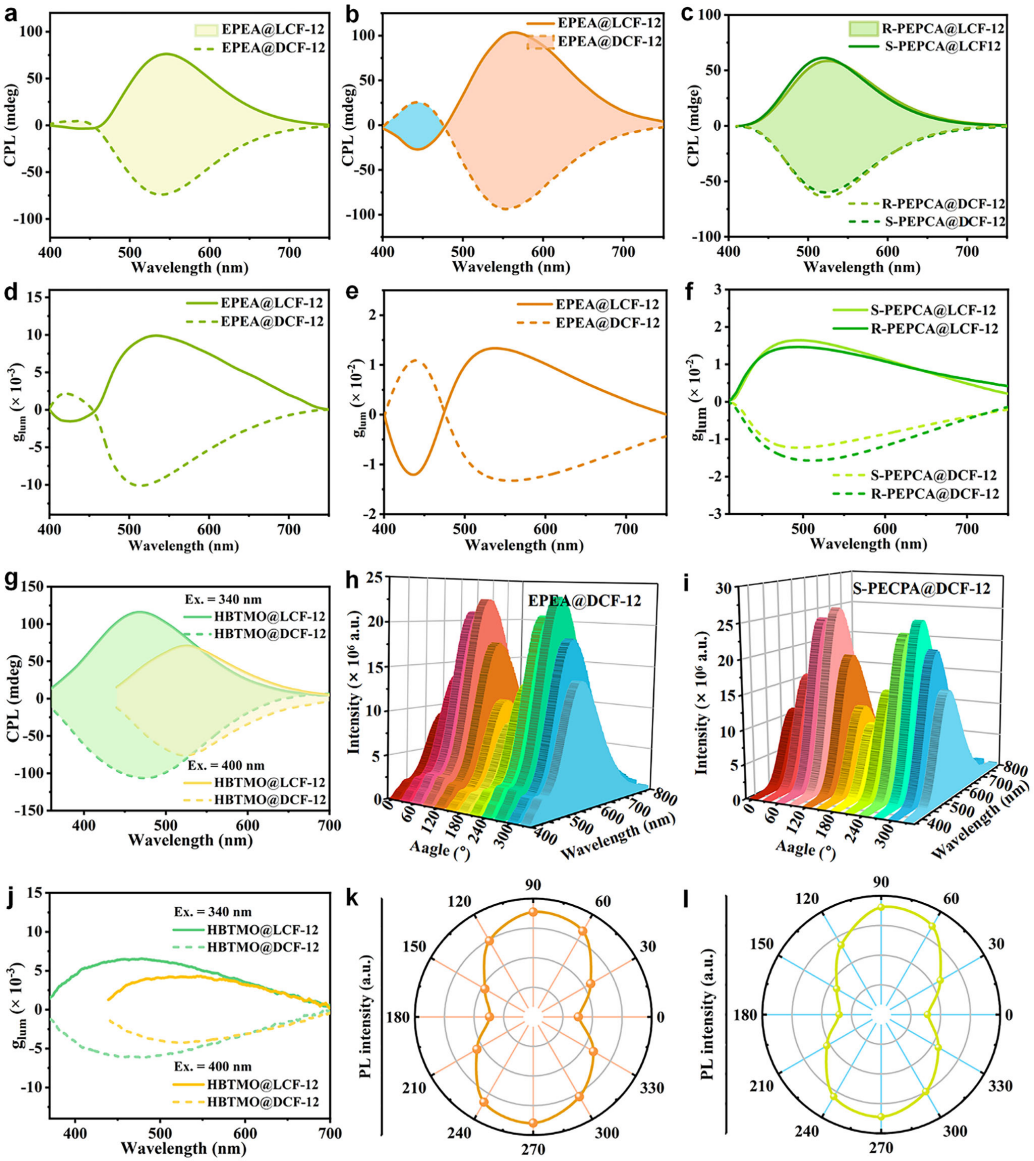
The chiroptical properties of host‐guest systems. a) CPL spectra of EPEA@DCF‐12 and EPEA@LCF‐12 after UV irradiation. b) CPL spectra of EPEA@DCF‐12 and EPEA@LCF‐12 after visible irradiation. c) CPL spectra of S‐PECPA@DCF‐12, R‐PECPA@DCF‐12, S‐PECPA@LCF‐12 and R‐PECPA@LCF‐12. d) *g*_lum_ values of EPEA@DCF‐12 and EPEA@LCF‐12 after UV irradiation. e) *g*_lum_ values of EPEA@DCF‐12 and EPEA@LCF‐12 after visible irradiation. f) *g*_lum_ values of S‐PECPA@DCF‐12, R‐PECPA@DCF‐12, S‐PECPA@LCF‐12 and R‐PECPA@LCF‐12. g) CPL spectra of HBTMO@DCF‐12 and HBTMO@LCF‐12 under excitation wavelength at 340 and 400 nm, respectively. h) Emission intensity of EPEA@DCF‐12 crystal powder at changed angles (0–360°) i) Emission intensity of S‐PECPA@DCF‐12 crystal powder at changed angles (0–360°). j) *g*_lum_ values of HBTMO@DCF‐12 and HBTMO@LCF‐12 under excitation wavelength at 340 and 400 nm, respectively. k) The maximum polarized emission spectra of EPEA@DCF‐12 crystal powder at changed angles (0–360°). l) The maximum polarized emission spectra of S‐PECPA@DCF‐12 crystal powder at changed angles (0–360°).

R/S‐PEPCA almost have no CPL signals in DMA solution, but relatively weak CPL signals in the solid state (Figure S13, Supporting Information). When R/S‐PEPCA are packaged into DCF‐12 and LCF‐12, the host‐guest composites exhibit the intense CPL signals (**Figure** **6**c). Importantly, the *g*_lum_ curves in Figure 6f demonstrated the amplification of *g*_lum_ values of R/S‐PEPCA‐loaded host‐guest systems compared to those of isolated R/S‐PEPCA. The *g*_lum_ values of R/S‐PEPCA at 465 nm were −4.82 × 10^‒3^ and +5.14 × 10^−3^, respectively (Figure S14f, Supporting Information). The *g*_lum_ values of R‐PEPCA@DCF‐12 and S‐PEPCA@LCF‐12 at 520 nm are up to ‒1.57 × 10^‒2^ and +1.64 × 10^‒2^, respectively, showing a about threefold improvement compared to that of R/S‐PEPCA. These results indicate that the orderly immobilization of R/S‐PEPCA into the periodic channels of the crystalline matrix contributes to the amplification of CPL properties and the enhancement of *g*_lum_ values.^[^ ^46^ ^]^

For fresh EPEA@DCF‐12 and EPEA@LCF‐12, the CPL spectra have one main peak at 545 nm (Figure 6a). After visible irradiation for 2 h, the mirror‐like CPL spectra of EPEA@DCF‐12 and EPEA@LCF‐12 show dual signals centered at 443 and 555 nm in agreement with their emission spectra (Figure 6b). It should be noted that the directions of two CPL signals are opposite, the CPL signal of EPEA@DCF‐12 at 443 nm is positive, but negative at 555 nm, and the *g*_lum_ values of EPEA@DCF‐12 and EPEA@LCF‐12 at 443 nm are calculated to be +1.09 × 10^‒2^ and −1.21 × 10^‒2^, as well as −1.34 × 10^‒2^ and +1.33 × 10^‒2^ at 555 nm, respectively (Figure 6e). According to the above investigation, the inversion of CPL signals can be attributed to [2+2] cycloaddition‐induced chirality inversion. As for DCF‐12, the cycloaddition reaction between TPE and EPEA took place after the visible irradiation. TPE molecules, which do not involve in this reaction, maintain the *M* configuration, while TPE involved in the formation of host‐guest adduct, the adduct presents an absolute configuration rather than trans‐cis isomerization. Therefore, the dual CPL signals with the opposite directions were realized. Moreover, these EPEA‐incorporated chiral MOFs can show the switchable CPL behaviors through precisely manipulating the reversible cycloaddition reaction via light and heat. To our knowledge, this result presents the first example of host‐guest MOFs showing the light‐ and thermal‐responsive CPL inversion.

Similarly, HBTM@DCF‐12 and HBTM@LCF‐12 also exhibit mirror‐like CPL spectra, with the intense emission peaks observed at 510 nm when excited at 360 nm, accompanied by maximum *g*_lum_ values of −5.19 × 10^‒3^ for HBTM@DCF‐12 and +5.56 × 10^‒3^ for HBTM@LCF‐12 (Figure S41, Supporting Information). Likewise, the highest emission intensities of HBTMO@DCF‐12 and HBTMO@LCF‐12 were found at 470 nm under 340 nm excitation, with corresponding *g*_lum_ values of −6.18 × 10^‒3^ and +6.56 × 10^‒3^, respectively. Importantly, when the excitation wavelength changes from 340 to 400 nm, the CPL bands shift from 470 to 530 nm, demonstrating the excitation wavelength‐dependent CPL property in HBTMO@DCF‐12 and HBTMO@LCF‐12 (Figure 6g,j). HBTMOA@DCF‐12 and HBTMOA@LCF‐12 displayed clear (‒)‐CPL and (+)‐CPL, respectively. The g_lum_ values were −4.22 × 10^‒3^ and +4.42 × 10^‒3^ (**Table** **2**; Figure S44, Supporting Information).

**Table 2:** Summary of chiroptical properties of guest‐encapsulated MOF_S_.

Compound	FLT@DCF‐12 FLT@LCF‐12	DMP@DCF‐12 DMP@LCF‐12	S‐PEPCA@DCF‐12 S‐PEPCA@LCF‐12	EPEA@DCF‐12 EPEA@LCF‐12	EPEA@DCF‐12 EPEA@LCF‐12	HBTMO@DCF‐12 HBTMO@LCF‐12
CPL	−, 467 nm	‒, 560 nm	‒, 522 nm	+, 443 nm^a^ ^)^ ‒, 555 nm^a^ ^)^	‒, 540 nm^b)^	‒, 470 nm^c)^ 530 nm^d)^
	+, 470 nm	+, 560 nm	+, 519 nm	‒, 442 nm^a^ ^)^ +, 555 nm^a^ ^)^	+, 545 nm^b)^	+, 470 nm^c)^ 530 nm^d)^
*g*_lum_	‒0.97 × 10^‒2^	‒1.18 × 10^‒2^	‒1.23 × 10^‒2^	+1.09× 10^‒2^ ‒1.34 × 10^‒2^	‒1.01 × 10^‒2^	‒6.18 × 10^‒3^ ^c)^ ‒4.26 × 10^‒3^ ^d)^
	+1.02 × 10^‒2^	+1.10 × 10^‒2^	+1.64 × 10^‒2^	‒1.33 × 10^‒2^ +1.21 × 10^‒2^	+0.99 × 10^‒2^	+6.56 × 10^‒3^ ^c)^ +4.32 × 10^‒3^ ^d)^

^a)^ under UV irradiation;

^b)^ after daylight irradiation;

^c)^ under excitation wavelength at 340 nm;

^d)^ under excitation wavelength at 400 nm.

It should be noted that the CPL activity was obtained on the macro level, which reflects the synergistic effect of chiral materials, while these enantiomeric crystals also have optical anisotropy, especially, the linear optical property can confuse the circular behaviors.^[^ ^47^ ^]^ In order to accurately evaluate the chiroptical properties, the linear polarized behaviors of EPEA@DCF‐12 and S‐PECPA@DCF‐12 were measured. As shown in Figure 5k,l, the multi‐angle linear polarized emission shows the varied intensities by rotating the linear polarizer from 0 to 360°, the maximum emission intensity presents an obvious polarization dependence, indicating that the CPL activity of these guest‐encapsulated MOFs is subjected to optical anisotropy. However, the conventional chiral spectrophotometers cannot accurately measure chiroptical properties and optical anisotropy simultaneously, thus it cannot give the corresponding data correction, and further investigations are worthy and needed.

In general, the chirality amplification could be strengthened through the encapsulation engineering of chiral MOFs, where the chirality transfer and enlargement could be realized benefiting from the multi‐level chirality including chiral helix, chiral space and chiral crystalline lattice. Remarkably, the luminescent behaviors can be modulated by the guest molecules, the CPL performance can be flexibly regulated through the host‐guest collaboration. The confinement effect, which can restrain the aggregation‐induced quenching effect of guest molecules and reduce the non‐radiative energy loss, together leading to the emission enhancement. The achiral guest molecules may form a regular and ordered accumulation along the helical channels, which is beneficial for chiral amplification of CPL. The encapsulation of guests into chiral porous MOFs offers a general approach to fabricate multi‐color CPL‐active materials and stimulus‐responsive dynamic CPL materials.

## 3 Multifunctional Applications for Anti‐Counterfeiting and WLED

Considering that these guests‐encapsulated MOFs exhibiting the multi‐color and multi‐mode emission with the chiroptical properties, have the advantages for multifunctional applications such as multiple anti‐counterfeiting and white‐light devices.

**Figure 7:**
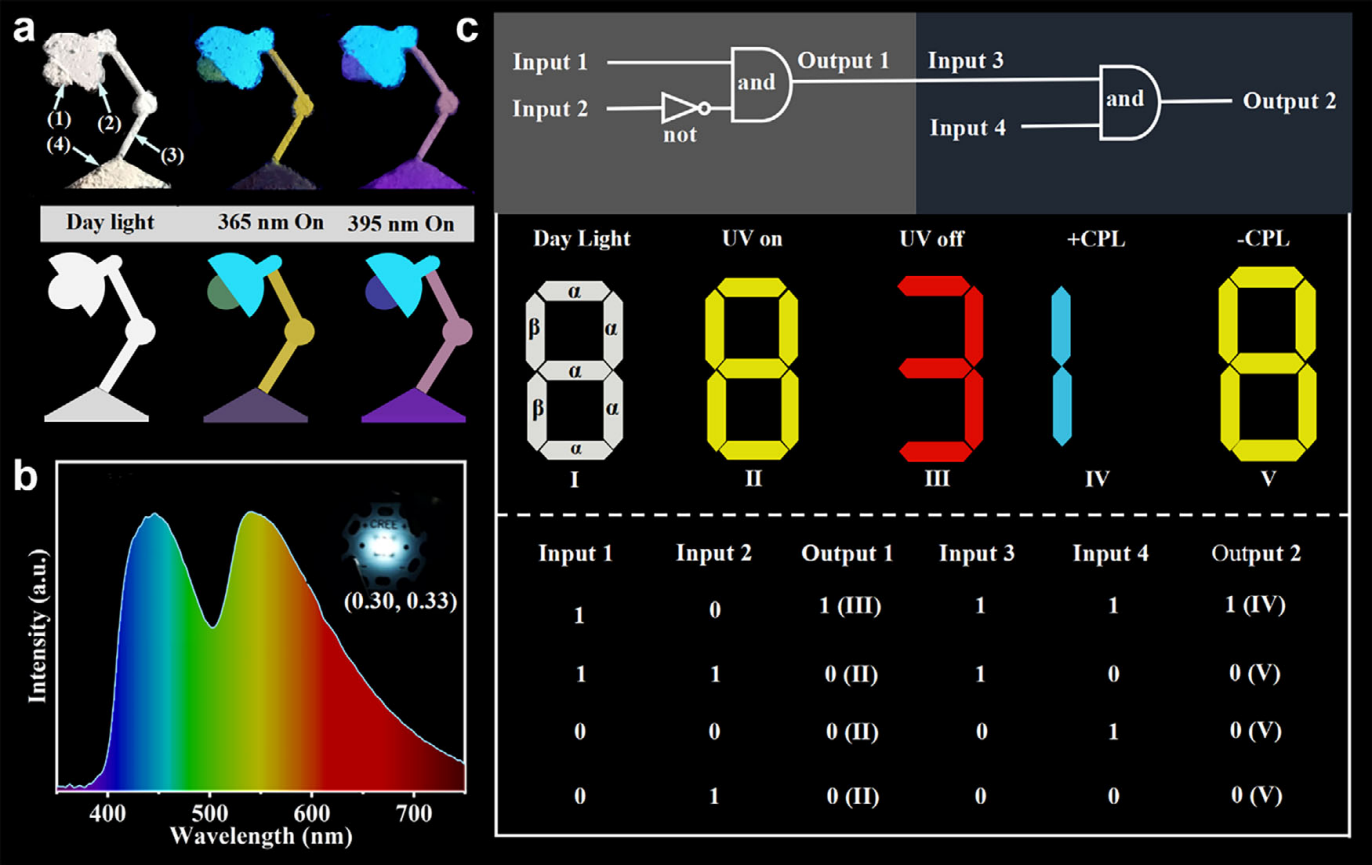
Optical applications of these composites. a) Photographs of multi‐level anti‐counterfeiting. (1): S‐PEPCA@DCF‐12, (2): FLT@DCF‐12, (3): EPEA@DCF‐12, (4): HBTMOA@DCF‐12. b) Luminescence behaviors of LED by using S‐PEPCA@DCF‐12 as phosphor powder. c) Optical logic gates whose operation is based on DMP@DCF‐12 with solvent‐ and thermo‐responsive solid‐state CPP characteristics, and EPEA@DCF‐12 owning the chirality inversion.

A “lamp” pattern, which consists of S‐PEPCA@DCF‐12 (lamp bead), EPEA@DCF‐12 (lamp shade), FLT@DCF‐12 (lamp post) and HBTMOA@DCF‐12 (lamp holder), shows white under daylight. Under 365 nm irradiation, the lamp bead, lamp shade, lamp post and lamp holder show green, yellow, blue and red fluorescence, respectively. When excited by 395 nm UV light, the color of lamp shade changes from green to blue, the color of lamp post changes from yellow to pink. After removing the ultraviolet light, the “lamp bead” shows a red afterglow, and other components have no emission. This multi‐step patterns greatly enhance the security and reliability of anti‐counterfeiting (**Figure** **7**a).

DMP@DCF‐12 with solvent‐ and thermo‐responsive solid‐state CPP characteristics, and EPEA@DCF‐12 owning the chirality inversion controlled by natural light/UV irradiation, were utilized to design an optical logic gate (Figure 7c). A number “8” (I) was made of DMP@DCF12 and EPEA@DCF12, corresponding to these two parts marked *α* and *β*. The introduction of benzyl alcohol (BzOH) and heating at 100 °C as Input 1 (In1) and Input 2 (In2). Meanwhile, the red RTP at 590 and 650 nm and yellow fluorescence emission were taken as “1” and “0” in Output 1 (Out1). The RTP performance of DMP@DCF‐12 was triggered by dropping BzOH, it can display red afterglow (III) after removing the 365 nm excitation sources, and the situation can return to its original state (I) through the thermal desorption of BzOH. The addition of BzOH is as Input 3 without heating, as well as the natural light irradiation for Input 4. As for Output 2, +CPL and −CPL signals were defined as “1” and “0”, respectively. Upon polarized light irradiation, the negative CPL signal (V) at 555 nm was obtained, and the positive CPL (VI) was obtained under natural light conditions. These stimuli‐responsive host‐guest chiral MOFs exhibit great potential in the design of logic gates.

The efficient white‐light emission of single‐phased S‐PEPCA@DCF‐12 makes it act as phosphor for white light emitting diode (WLED). We fabricated the WLED by coating the S‐PEPCA@DCF‐12 powder on a commercial 310 nm UV chip. As displayed in Figure 7b, the corresponding CIE coordinate is determined as (0.30, 0.33), which closely approaches the ideal white‐light coordinates (0.33, 0.33). In addition, The PL spectrum of the WLED exhibits a high color rendering index (CRI) of 81.2 and a correlated color temperature (CCT) of 7074 K. These results indicate that the single‐phased white‐light emitter S‐PEPCA@DCF‐12 has the bright future for solid‐state white emission devices.

## 4 Conclusions

To sum up, eight pairs of guest‐encapsulated chiral MOFs were prepared through in‐situ method. The multi‐color and multi‐mode CPL can be achieved through three approaches including DET, light‐ and thermal‐responsive [2+2] reaction and ESIPT. Based on DET, the excitation‐dependent luminescence and RTP can be presented with the encapsulation of pyrene‐based molecules. DMP@DCF‐12/DMP@LCF‐12, R/S‐PEPCA@DCF‐12 and R/S‐PEPCA@LCF‐12 exhibited NIR‐CPP with high *Φ*_Phos_ over 75% in air, and the *g*_lum_ values of S‐PEPCA@LCF‐12 and R‐PEPCA@DCF‐12 are +1.64 × 10^‒2^ and −1.57 × 10^‒2^. The single‐phased white‐light CPL was achieved for WLED by using S‐PEPCA‐encapsulated DCF‐12. Notably, the introduction of the specific pyrene‐cored molecule containing vinyl bonds exhibits the reversible photochromism and regular chirality inversion through the [2+2] cycloaddition reaction. With the introduction of molecules featuring ESIPT property, the excitation‐responsive CPF can be obtained. Importantly, the host‐guest structures of FLT‐encapsulated MOFs could be obtained via single‐crystal diffraction, providing direct evidences to prove that the chirality of guest molecules can be endowed through the chirality transfer of chiral confined space. These results demonstrate that the encapsulation of guest emitters can efficiently enhance chirality amplification, and offers a universal strategy to develop wavelength‐, photo‐ and thermal‐responsive CPL. This work not only offers new insights into the development of CPL through guest‐modulated MOFs, but also lays the groundwork for designing new multi‐color and stimuli‐responsive CPL‐active materials.

## 5 Experimental Section

### Materials

All reagents and solvents were purchased commercially. Ethyl alcohol (EtOH), N,N‐dimethylformamide (DMF) and N,N‐dimethylacetamide (DMA) (China National Pharmaceutical Reagent Co., Ltd.), Benzyl alcohol (BzOH) (Aladdin), (1,1,2,2‐tetra(pyridin‐4‐yl)ethene) (TPE) (Chemsoon), D/L‐camphoric acid (D/L‐cam) (TCI), Fluoranthene (FLT) (TCI), 2,7‐dimethylpyrene (DMP) (Bidepharm), 2‐hydroxy‐5‐methylbenzaldehyde (Aladdin), 2‐hydroxy‐5‐methoxybenzaldehyde (Aladdin), 2‐aminothiophenol (Aladdin), sodium pyrosulfite (Na_2_S_2_O_5_) (Aladdin), hexamethylenetetramine (Aladdin), trifluoroacetic acid (Aladdin), (*E*)‐ethyl 3‐(pyren‐1‐yl)acrylate (EPEA) (Tensus Biotech), (R/S)‐N‐(1‐(pyridin‐2‐yl)ethyl)pyrene‐1‐carboxamide (R/S‐PEPCA) (Tensus Biotech).

### Synthesis of 2‐(Benzo[d]thiazol‐2‐yl)‐4‐Methylphenol (HBTM)

2‐hydroxy‐5‐methylbenzaldehyde (10.0 mmol, 1.4 g), 2‐aminothiophenol (11.0 mmol, 1.4 g), and sodium pyrosulfite (Na_2_S_2_O_5_, 10.0 mmol, 1.9 g) were mixed in a 100 mL round‐bottom flask with 50 mL of DMF. Then, the mixture was heated to 110 °C and refluxed for 2 h in an oil bath condition. The TLC template was employed to monitor the progress of the reaction. The mixture was cooled to room temperature after the reaction was finished and then 500 mL of water was added to the mixture, and the crude product was precipitated from the mixture. The precipitate was filtered and washed three times with cold water, and the obtained crude product was purified by flash column chromatography. Finally, a white solid product (2.3 g) was obtained. Yield: 92%. ^1^H NMR (400 MHz, CDCl_3_) δ 12.34 – 12.30 (s, 1H), 7.99 – 7.93 (d, *J* = 8.2 Hz, 1H), 7.90 – 7.84 (d, *J* = 8.0 Hz, 1H), 7.52 – 7.42 (dd, *J* = 15.0, 7.1 Hz, 2H), 7.42 – 7.34 (t, *J* = 7.7 Hz, 1H), 7.21 – 7.15 (d, *J* = 8.5 Hz, 1H), 7.04 – 6.98 (dd, *J* = 8.4, 1.8 Hz, 1H), 2.48 – 2.24 (s, 3H); ^13^C NMR (100 MHz, CDCl_3_) *δ* = 169.53, 155.93, 152.05, 133.85, 132.73, 128.78, 128.43, 126.73, 125.53, 122.23, 121.59, 117.77, 116.46, 20.59.

### Synthesis of 2‐(Benzo[d]thiazol‐2‐yl)‐4‐Methoxyphenol (HBTMO)

2‐hydroxy‐5‐methoxybenzaldehyde (10.0 mmol, 1.5 g), 2‐aminothiophenol (11.0 mmol, 1.4 g), and sodium pyrosulfite (Na_2_S_2_O_5_, 10 mmol, 1.9 g) were mixed in a 100 mL round‐bottom flask with 50 mL of DMF. Then, the mixture was heated to 110 °C and refluxed for 2 h in an oil bath condition. The TLC template was employed to monitor the progress of the reaction. The mixture was cooled to room temperature after the reaction was finished and then 500 mL of water was added to the mixture, and the crude product was precipitated from the mixture. The precipitate was filtered and washed three times with cold water, and the obtained crude product was purified by flash column chromatography. Finally, a yellow solid product (2.1 g) was obtained. Yield: 83%. ^1^H NMR (400 MHz, CDCl_3_) δ 12.23 – 11.94 (s, 1H), 8.02 – 7.95 (d, *J* = 8.2 Hz, 1H), 7.93 – 7.86 (d, *J* = 8.0 Hz, 1H), 7.55 – 7.46 (m, 1H), 7.45 – 7.37 (t, *J* = 7.8 Hz, 1H), 7.18 – 7.14 (d, *J* = 2.8 Hz, 1H), 7.08 – 6.96 (m, 2H), 3.87 – 3.82 (s, 3H); ^13^C NMR (100 MHz, CDCl_3_) δ = 169.16, 152.51, 152.36, 152.04, 132.75, 126.82, 125.66, 122.33, 121.61, 119.92, 118.82, 116.51, 111.98, 56.08.

### Synthesis of 3‐(Benzo[d]thiazol‐2‐yl)‐2‐Hydroxy‐5‐Methoxybenzaldehyde (HBTMOA)

Compound HBTMO (5.0 mmol, 1.3 g) and hexamethylenetetramine (15.0 mmol, 2.1 g) were mixed in a 50 mL round‐bottom flask with 20 mL of trifluoroacetic acid. Then, the mixture was stirred vigorously and refluxed at 100 °C for 12 h. After the resulting mixture was cooled to room temperature, a 4 mol L^−1^ HCl solution (50 mL) and 150 mL of water were added and an orange‐yellow precipitate appeared. The precipitate was filtered and dissolved in dichloromethane. After extraction with dichloromethane and saturated brine three times, the organic phase was collected by reduced pressure distillation, and the obtained crude product was purified by flash column chromatography. Finally, an orange‐yellow solid product (1.1 g) was obtained. Yield: 76%. ^1^H NMR (400 MHz, CDCl_3_) δ 12.92 – 12.28 (s, 1H), 10.60 – 10.32 (s, 1H), 8.00 – 7.94 (d, *J* = 8.1 Hz, 1H), 7.91 – 7.85 (d, *J* = 8.0 Hz, 1H), 7.56 – 7.48 (dd, *J* = 16.2, 7.6 Hz, 2H), 7.44 – 7.36 (m, 2H), 3.86 – 3.82 (s, 3H); ^13^C NMR (100 MHz, CDCl_3_) δ = 190.23, 167.01, 155.35, 152.25, 151.62, 133.18, 127.08, 126.15, 124.38, 122.60, 121.74, 121.69, 119.56, 114.85, 56.22.

### Synthesis of Guests@DCF‐12

D‐cam (0.099 mmol), TPE (0.030 mmol), Zn(NO_3_)_2_·6H_2_O (0.151 mmol) and guest (0.015 mmol) were dissolved in solution of DMA (4 mL), BzOH (2 mL) and Deionized water (1 mL) in the 23 mL glass vial, and the vial was placed in an oven at 100 °C for 72 h. The resulting crystals were collected and washed with DMA several times. Guests@LCF‐12 were prepared by using L‐cam instead of D‐cam.

### LED Preparation

For the UV‐pumped white LED, the grounded S‐PEPCA@DCF‐12 were blended with PMMA to form a well‐distributed mixture; then, the composite was coated on the surface of a 310 nm UV LED chip and cured for 30 min at room temperature.

### Characterizations of Organic Molecules and Host‐Guest Systems

Powder X‐ray diffraction (PXRD) patterns were collected on a Bruker D8 ADVANCE diffractometer with Cu Kα (λ = 1.5418 Å) radiation. Measurements were operated power was 40 KV, 40 mA, and a 2*θ* range of 5–50° at room temperature with a counting time of 0.2 s per step. Thermogravimetric analysis (TGA) was performed on a PerkinElmer TG‐7 analyzer heated from 30 to 750 °C at the atmosphere with a ramp rate of 5 °C min^−1^. Ultraviolet‐visible (UV‐vis) absorption spectra were collected on a PerkinElmer Lambda spectrophotometer and SHIMADZU UV‐2600 UV‐vis spectrophotometer. The fluorescence and phosphorescence spectra were conducted on Edinburgh FLS1000 with xenon lamp and nanosecond flash‐lamp. High‐resolution mass spectra were obtained on SCIEX X‐500R QTOF (ESI mode). ^1^H and ^13^C NMR spectra were collected on German BRUKER AVANCE III 400 MHz, and deuterated chloroform (CDCl_3_) as solvent.

### Single Crystal X‐Ray Diffraction

Single‐crystal X‐ray diffraction data were obtained from Bruker SMART APEX CCD diffractometer equipped with Mo‐Kα radiation (λ = 0.71073 Å) by *ω* scan mode at room temperature. The structure was determined by direct methods and refined by the full‐matrix least‐squares method with the *SHELX* ^[^ ^48^ ^]^ and *OLEX2.0* ^[^ ^49^ ^]^ program package. All non‐hydrogen atoms were located successfully from Fourier maps and were refined anisotropically. The H atoms on the ligands were placed in idealized positions and refined using a riding model. The disordered guest molecules could not be located successfully from Fourier maps, and the highly disordered lattice guest molecules were removed using the *SQUEEZE* procedure by *PLATON*.^[^ ^50^ ^]^

### Chiroptical Measurement

CPL spectra for all samples were recorded on a JASCO CPL‐300 spectrophotometer in the solid state with scanning speed. The basic mode is used. Scanning speed, E_x_ slit width, E_m_ slit width, and accumulations are 100 nm min^−1^, 2000 um, 2000 um, and 4, respectively. The circular dichroism (CD) spectra were measured from 200–800 nm range using JASCO J‐1500 circular dichroism spectrometer. The samples were prepared by using the method for infrared measurement. A mixture of crystals and KBr (crystal/KBr 1:400, weight ratio, total weight of 100 mg) was finely ground and pressed into a transparent pellet with a diameter of 13 mm. The pellet was directly used for measurement of CD. The wavelength and bandwidth of the monochromator were set to 280 nm and 1.0 nm, and the time‐per‐point of each sampling point was 0.5 s.

### Phosphorescence Quantum Yields

The quantum yields (QYs) performed on FLS1000 Edinburgh photoluminescence spectrometer (under the optimal excitation wavelengths based on the spectra measurement). The phosphorescence QYs was calculated using the following equation by reference to the literatures.^[^ ^51^ ^]^ As follows: *Ф*_Phos_ = *Ф*_PL_ × A_phos_/(A_FL_ + A_Phos_). Herein, *Ф*_Phos_ is the phosphorescence QYs, *Ф*_PL_ is the PLQY; A_FL_ and A_Phos_ are integral peak areas of fluorescence and phosphorescence.

### Theoretical Simulation

Time dependent density functional theory (TD‐DFT) of B3LYP/6‐31 G (d,p) level was used to calculate the HOMO orbits, LUMO orbits and excitation energy in Gaussian 16 (version A.03) software. VMD and Multiwfn 3.8 software were used to visualize HOMO and LUMO orbits.^[^ ^52^ ^]^ The density functional theory (DFT) calculations were performed using a Dmol3 module of Material Studio 2020.^[^ ^53^ ^]^ The generalized gradient approximation (GGA) method with Perdew‐Burke‐Ernzerhof (PBE) function was employed to describe the interactions between core and electrons. The force and energy convergence criterion were set to 0.002 Ha Å^−1^ and 10^−5^ Ha, respectively. When the optimization was completed, the ESP and mulliken charge calculations were performed. The binding energy (ΔE) was calculated as ΔE(eV) = 27.212*(E_total_(Ha) – E_1_(Ha) – E_2_(Ha)) where the E_total_ is the energy of the optimized system; E_1_ is the energy of the material; E_2_ is the energy of micromolecule.

[CCDC 2391055–2391056 contains the supplementary crystallographic data for this paper. These data can be obtained free of charge from The Cambridge Crystallographic Data Centre via www.ccdc.cam.ac.uk/data_request/cif.]

## Conflict of Interest

The authors declare no conflict of interest.

## Supporting information



Supporting Information

## Data Availability

The data that support the findings of this study are available from the corresponding author upon reasonable request.
