# Supplementary material for: Multi‐Stimuli‐Responsive Circularly Polarized Luminescence with Handedness Inversion and Near‐Infrared Phosphorescence in Chiral Metal‐Organic Framework Platform for White Light Emission and Information Encryption
Source: Adv Sci (Weinh). 2025 Mar 17;12(18):2502784. doi: 10.1002/advs.202502784 (PMC12079507; doi:10.1002/advs.202502784)
Supplement: Supplementary file 1 — Supporting Information [file ADVS-12-2502784-s001.docx]

Supporting Information

**Multi-Stimuli-Responsive Circularly Polarized Luminescence with Handedness Inversion and Near-Infrared Phosphorescence in Chiral Metal-Organic Framework Platform for White Light Emission and Information Encryption**

*Kun Zhang, Ni Dan, Ruo-Yu Zhang, Jiaojiao Wei, Rui-Xue Tian, Yongfan Zhang, Hong-Ru Fu,^*^ Mei Qiu,^*^ Lu-Fang Ma,^*^ Shuang-Quan Zang^*^*

K. Zhang, N. Dan, R. Zhang, J. Wei, R. Tian, H. Fu, L. Ma

College of Chemistry and Chemical Engineering, Luoyang Normal University, Luoyang 471934, China

E-mail: hongrufu2015@163.com; [mazhuxp@126.com](mailto:mazhuxp@126.com)

K. Zhang, N. Dan

College of Materials and Chemical Engineering, China Three Gorges University, Yichang 443002, China

Y. Zhang, M. Qiu

College of Chemistry, Fuzhou University, Fuzhou 350116, China

M. Qiu

College of Chemistry and Materials, Jiangxi Agricultural University, Nanchang, Jiangxi 330045, China

E-mail: qium@jxau.edu.cn

S. Zang

College of Chemistry, Zhengzhou University, Zhengzho 450001, China

E-mail: zangsqzg@zzu.edu.cn

| **Table of Contents** | |
| --- | --- |
| **Figure S1** | The structure of DCF-12. |
| **Figure S2** | 3D structure of FLT@DCF-12. |
| **Figures S3–S5** | PXRD patterns of Guests@MOFs. |
| **Figures S6–S8** | TG curves of Guests@MOFs. |
| **Figures S9–S16** | Photophysical properties of guests |
| **Figure S18** | (a) The luminescence emission of DCF-12 and LCF-12 at excitation wavelengths of 400 nm. (b) Fluorescence lifetime decay profiles of DMP@DCF-12 at solid. (c) Fluorescence lifetime decay profiles of DMP@LCF-12 at solid. (d) The solid-state absorption of TPE. (e) The solid-state absorption of D-cam and L-cam. (f) The solid-state absorption of DCF-12 and LCF-12. |
| **Figures S19–S24** | The absorbance in DMA solution of guests at different concentrations from 1 × 10^‒5^ to 1 × 10^‒3^ mol/L and solid-state. |
| **Figure S25** | (a) The prompt emission of FLT@DCF-12 under different excitation wavelengths. (b) The lifetime of FLT@DCF-12 at 437 and 466 nm, respectively. (c) The solid-state absorption of FLT@DCF-12 and FLT@LCF-12. (d) CD spectra of FLT@DCF-12 and FLT@LCF-12. (e) CPL spectra of FLT@DCF-12 and FLT@LCF-12. (f) The *g*_lum_ values of FLT@DCF-12 and FLT@LCF-12. |
| **Figure S26** | (a) The luminescence emission of DMP@DCF-12 under different excitation wavelengths from 300 to 410 nm. (b) The luminescence emission of DMP@DCF-12 under different excitation wavelengths from 420 to 520 nm. (c) The luminescence emission of DMP@DCF-12 under different excitation wavelengths from 530 to 590 nm. (d) CIE coordinate diagram of the luminescence spectra of DMP@DCF-12 with the excitation wavelengths changing from 410 to 590 nm. (e) Excitation-phosphorescence mapping of the DMP@DCF-12 under ambient conditions with different excitation wavelengths from 420 to 520 nm. (f) Temperature-dependent afterglow spectra for DMP@DCF-12 with a delayed time of 0.5 ms from 77 to 390 K. (g) Time-resolved decay curves for emission at 592 and 655 nm at 77 K with a delayed time of 0.5 ms. (h) Temperature-dependent luminescence emission spectra for DMP@DCF-12 from 77 to 390 K. (i) The prompt lifetime at 500, 520 540 nm under different temperatures. |
| **Figure S27** | (a) The photoluminescence spectra of DMP@DCF-12 under different excitation wavelengths from 340 to 440 nm with a delayed time of 0.5 ms. (b) CIE coordinate diagram of the photoluminescence spectra of DMP@DCF-12 with the excitation wavelengths changing from 290 to 390 nm with a delayed time of 0.5 ms. (c) and (d) Time-resolved decay curves for emission of DMP@DCF-12 and DMP@LCF-12 at 590 and 650 nm with a delayed time of 0.5 ms under ambient conditions. |
| **Figure S28** | (a) The visible luminescence of the solvent-activated samples under UV light. (b) The visible afterglow of the solvent-activated samples. (c) The solvent-activated samples were dried under 120 °C for 5 hours. (d) The visible luminescence of the solvent-activated samples was recovered under UV light. (e) The similar visible afterglow of the solvent-activated samples could be observed again. The single crystal sample was treated by n-hexane, EA, DCM, Ether, 1,4-dioxane, MeOH, DMA, H_2_O and BzOH, respectively. |
| **Figure S29** | (a) The phosphorescence spectra of DMP@DCF-12 after being heated under 120 ℃ for 5 hours. (b) Time-resolved decay curves for emission at 565, 565, 600 and 610 nm upon excitation at 300, 320 340 and 360 nm under ambient temperature. (c,d) PXRD patterns of DMP@DCF-12 after the cycling experiment. |
| **Figure S30** | (a) The luminescence emission of DMP@DCF-12 and DMP@LCF-12 at excitation wavelengths of 410 nm. (b) The lifetime of DMP@DCF-12 and DMP@LCF-12 at 560 nm. (c) CPL spectra of DMP@DCF-12 and DMP@LCF-12. (d) The *g*_lum_ values of DMP@DCF-12 and DMP@LCF-12. (e) The solid-state absorption of DMP@DCF-12 and DMP@LCF-12. (f) CD spectra of DMP@DCF-12 and DMP@LCF-12 in solid-state. |
| **Figure S31** | (a) The photographs for crystalline samples of DMP@DCF-12 under daylight, 365 nm, and after being dropped BzOH and turning off 365 nm irradiation. (b) CPL spectra of DMP@DCF-12 and DMP@LCF-12 under ambient conditions, when the samples were treated under 120 ℃ for 5 hours. (c) The *g*_lum_ values of DMP@DCF-12 and DMP@LCF-12, when the samples were treated under 120 ℃ for 5 hours. (d) CPL spectra of DMP@DCF-12 and DMP@LCF-12 under ambient conditions, when the samples were dropped by BzOH after 5 hours. (e) The *g*_lum_ values of DMP@DCF-12 and DMP@LCF-12, when the samples were dropped by BzOH after 5 hours. (f) The phosphorescence intensity at 590 nm of DMP@DCF-12 during the 8 cycles under ambient conditions. (g) The phosphorescence lifetimes at 590 nm of DMP@DCF-12 after 8 cycles under ambient conditions. |
| **Figure S32** | (a) The prompt lifetime of S-PEPCA@DCF-12 at 510 nm under room temperatures. (b) Time-resolved decay curves for emission of S-PEPCA@DCF-12 at 600 and 650 nm at 77 K with a delayed time of 0.5 ms. (c) The prompt lifetime of R-PEPCA@LCF-12 at 510 nm under room temperatures. (d) Time-resolved decay curves for emission of R-PEPCA@LCF-12 at 600 and 650 with a delayed time of 0.5 ms under ambient conditions. (e) The prompt lifetime of S-PEPCA@LCF-12 at 510 nm under room temperatures. (f) The maximum polarized emission spectra of S-EPECA@LCF-12 crystal powder at changed angles (0‒360°). (g) The maximum polarized emission spectra of S-PEPCA@LCF-12 crystal powder at changed angles (0‒360°). (h) The solid-state absorption of S-PEPCA@DCF-12, R-PEPCA@DCF-12, S-PEPCA@LCF-12 and R-PEPCA@LCF-12. (i) CD spectra of S-PEPCA@DCF-12, R-PEPCA@DCF-12, S-PEPCA@LCF-12 and R-PEPCA@LCF-12 in solid-state. |
| **Figure S33** | (a) The photoluminescence spectra of S-PEPCA@DCF-12 under different excitation wavelengths from 290 to 390 nm with a delayed time of 0.5 ms. (b) CIE coordinate diagram of the photoluminescence spectra of S-PEPCA@DCF-12 with the excitation wavelengths changing from 290 to 390 nm with a delayed time of 0.5 ms. |
| **Figure S34** | (a) The photographs of fluorescence and afterglow for crystalline samples of DMP@DCF-12 under daylight, 365 nm, and after dropping BzOH and turning off 365 nm irradiation. (b) Excitation-phosphorescence mapping of the DMP@DCF-12 after being heated under 120 ℃ for 5 hours with different excitation wavelengths from 280 to 450 nm. (c) The phosphorescence spectra of DMP@DCF-12 after being heated under 120 ℃ for 5 hours. (d) Time-resolved decay curves for emission at 593 nm at ambient temperature with a delayed time of 0.5 ms. (e) PXRD patterns of DMP@DCF-12 and the dried DMP@DCF-12. |
| **Figure S35** | (a) Emission of EPEA@DCF-12 upon visible light irradiation under different times. (b) The CIE coordinates of DCF-1 and LCF-13. |
| **Figure S36** | The EPEA@DCF-12 structures of the simulation results. |
| **Figure S37** | The mass spectrum: EPEA@DCF-12 which was exposed in visible for 2 hours, was dissolved in aqueous solution by concentrated hydrochloric acid (37% HCl). |
| **Figure S38** | (a) The prompt lifetime of EPEA@DCF-12 at 437 and 535 nm at room temperature under ring opening state, namely, EPEA@DCF-12 recovered to the initial state through the heating treatment at 100 ℃. (b) The prompt lifetime of EPEA@DCF-12 at 437 and 535 nm under heating treatment at 100 ℃. (c) The prompt lifetime of EPEA@LCF-12 at 437 and 535 nm at room temperature under ring opening state, namely, EPEA@LCF-12 recovered to the initial state through the heating treatment at 100 ℃. (d) The prompt lifetime of EPEA@LCF-12 at 437 and 535 nm under heating treatment at 100 ℃. (e) The emission intensity of EPEA@LCF-12 crystal powder at changed angles (0‒360°). (f) The maximum polarized emission spectra of EPEA@LCF-12 crystal powder at changed angles (0‒360°). (g) The [2+2] cycloaddition reaction between EPEA and TPE shows the excellent reversibility. The emission behaviors at 437 and 535 nm show the good recoverability after 10 times. (h) CIE coordinate diagram of the luminescence spectra of EPEA@DCF-12 upon visible light irradiation under different times (0‒120 min). (i) luminescence emission of EPEA@DCF-12 encapsulated with different volumes of EPEA solution (1 × 10^‒2^ mol/L in DMA). |
| **Figure S39** | (a) The solid-state absorption of EPEA@DCF-12 and EPEA@LCF-12. (b) CD spectra of EPEA@DCF-12 and EPEA@LCF-12 under ring opening state. (c) CD spectra of EPEA@DCF-12 and EPEA@LCF-12 under ring closing state. |
| **Figure S40** | The photographs of EPEA@DCF-12 under daylight and UV. |
| **Figure S41** | (a) CIE coordinate diagram of the luminescence spectra of HBTM@DCF-12 with the excitation wavelengths changing from 305 to 465 nm. (b) The prompt lifetime of HBTM@DCF-12 at 425 465 and 520 nm at room temperature. (c) The solid-state absorption of HBTM@DCF-12 and HBTM@LCF-12. (d) CD spectra of HBTM@DCF-12 and HBTM@LCF-12. (e) CPL spectra of HBTM@DCF-12 and HBTM@LCF-12. (f) The *g*_lum_ values of HBTM@DCF-12 and HBTM@LCF-12. |
| **Figure S42** | (a) Emission and excitation spectra of HBTM@DCF-12 at 365 nm. (b) Emission and excitation spectra of HBTM@DCF-12 at 385 nm. (c) Emission and excitation spectra of HBTM@DCF-12 under different excitation wavelengths from 405 to 500 nm. (d) The prompt lifetime of HBTM@DCF-12 at 437 and 475 nm at room temperature. (e) The prompt lifetime of HBTM@DCF-12 at 495 and 510 nm at room temperature. (f) The prompt lifetime of HBTM@DCF-12 at 520 and 530 nm at room temperature. |
| **Table S1** | Emission peaks and their lifetimes of HBTM@DCF-12 at different excitation wavelengths. |
| **Figure S43** | (a) The prompt lifetime of HBTMO@DCF-12 at 435 and 470 nm at room temperature. (b) The solid-state absorption of HBTMO@DCF-12 and HBTMO@LCF-12. (c) CD spectra of HBTMO@DCF-12 and HBTMO@LCF-12. |
| **Figure S44** | (a) The prompt lifetime of HBTMOA@DCF-12 at 437 495 and 595 nm at room temperature. (b) The solid-state absorption of HBTMOA@DCF-12 and HBTMOA@LCF-12. (c) CD spectra of HBTMOA@DCF-12 and HBTMOA@LCF-12. (d) The prompt lifetime of HBTMOA@DCF-12 at 520 and 530 nm at room temperature. (e) CPL spectra of HBTMOA@DCF-12 and HBTMOA@LCF-12. (f) The *g*_lum_ values of HBTMOA@DCF-12 and HBTMOA@LCF-12. |
| **Figure S45–S47** | Phosphorescence and fluorescence quantum yields of DMP@DCF-12. |
| **Figure S48–S50** | DFT-calculated HOMO and LUMO of guests and TPE. |
| **Figure S51** | The energy levels of TPE and the guest emitters. |
| **Table S2** | The crystallographic parameters of FLT@DCF-12 and FLT@LCF-12. |
| **Table S3** | The yields of DCF-12/LCF-12 and guest-encapsulated MOFs. |
| **Figure S52–S53** | NMR spectrum of HBTM. |
| **Figure S54–S55** | NMR spectrum of HBTMO. |
| **Figure S56–S57** | NMR spectrum of HBTMOA. |

1. **The structures of DCF-12 and FLT@DCF-12**

**
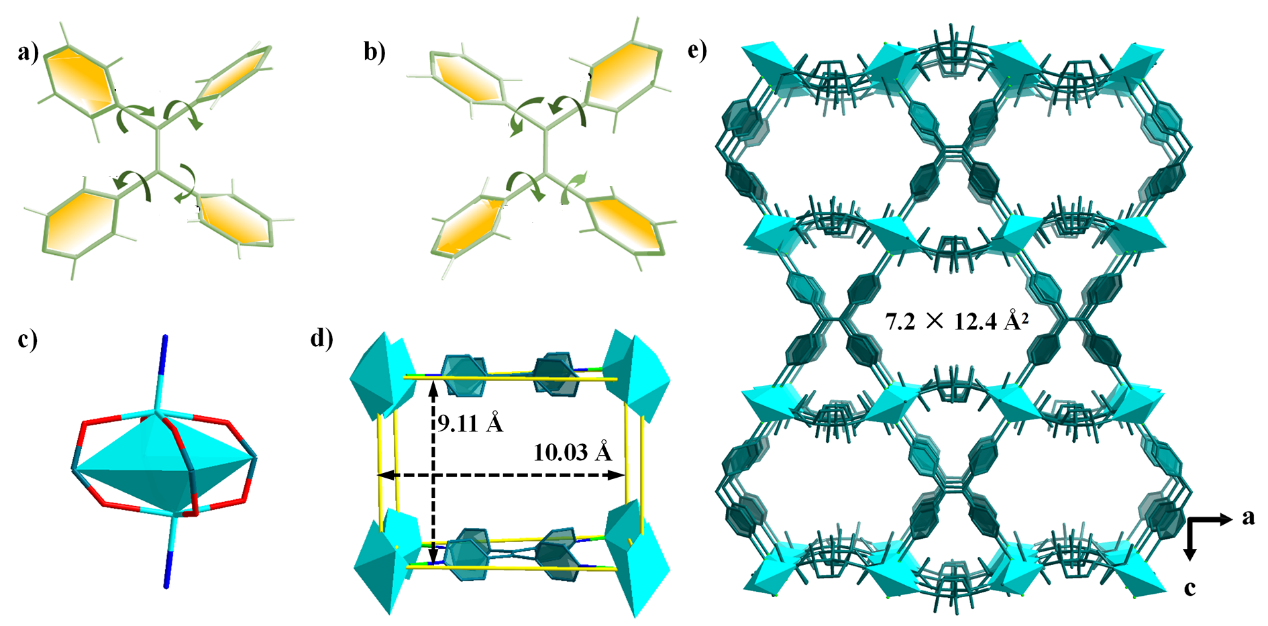
**

**Figure S1.** 3D structure of DCF-12.

**
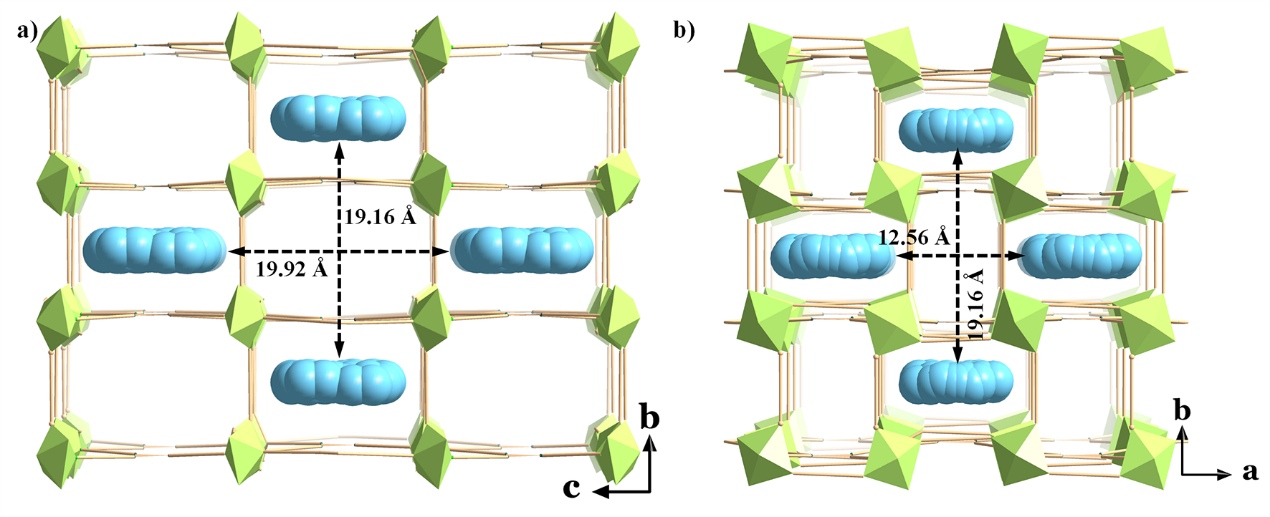
**

**Figure S2.** 3D structure of FLT@DCF-12 in the direction of a (a) and c (b) axis, respectively.

1. **PXRD and TGA properties of Guests@MOFs**


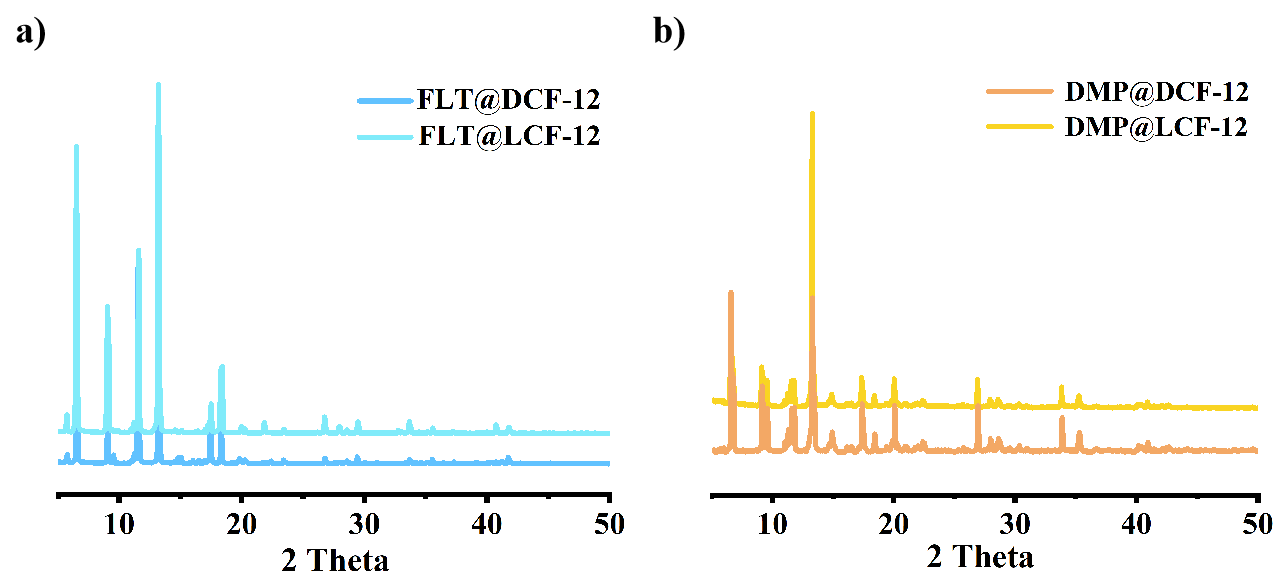


Figure **S3**. (a) PXRD patterns of FLT@DCF-12 and FLT@LCF-12. (b) PXRD patterns of DMP@DCF-12 and DMP@LCF-12.

**
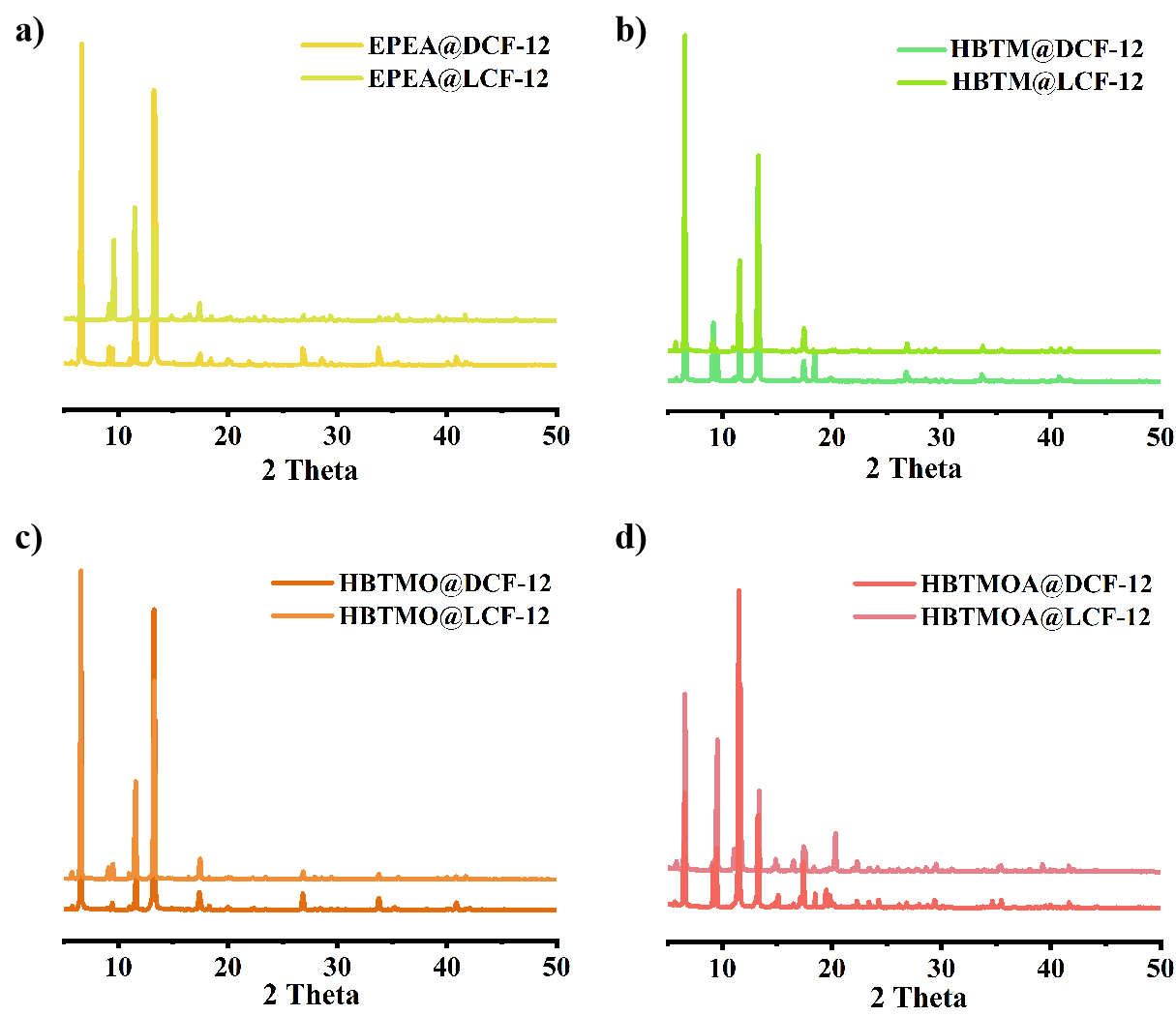
**

Figure **S4**. (a) PXRD patterns of EPEA@DCF-12 and EPEA@LCF-12. (b) PXRD patterns of HBTM@DCF-12 and HBTM@LCF-12. (c) PXRD patterns of HBTMO@DCF-12 and HBTMO@LCF-12. (d) PXRD patterns of HBTMOA@DCF-12 and HBTMOA@LCF-12.


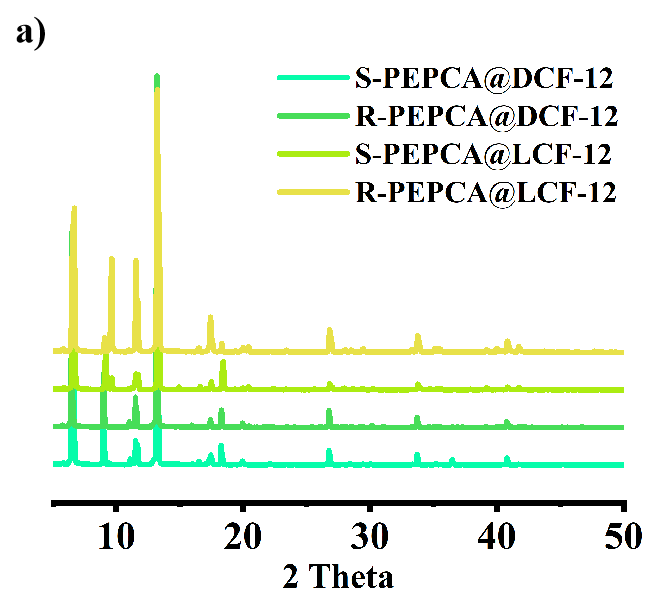


Figure **S5**. (a) PXRD patterns of S-PEPCA@DCF-12, R-PEPCA@DCF-12, S-PEPCA@LCF-12 and R-PEPCA@LCF-12.


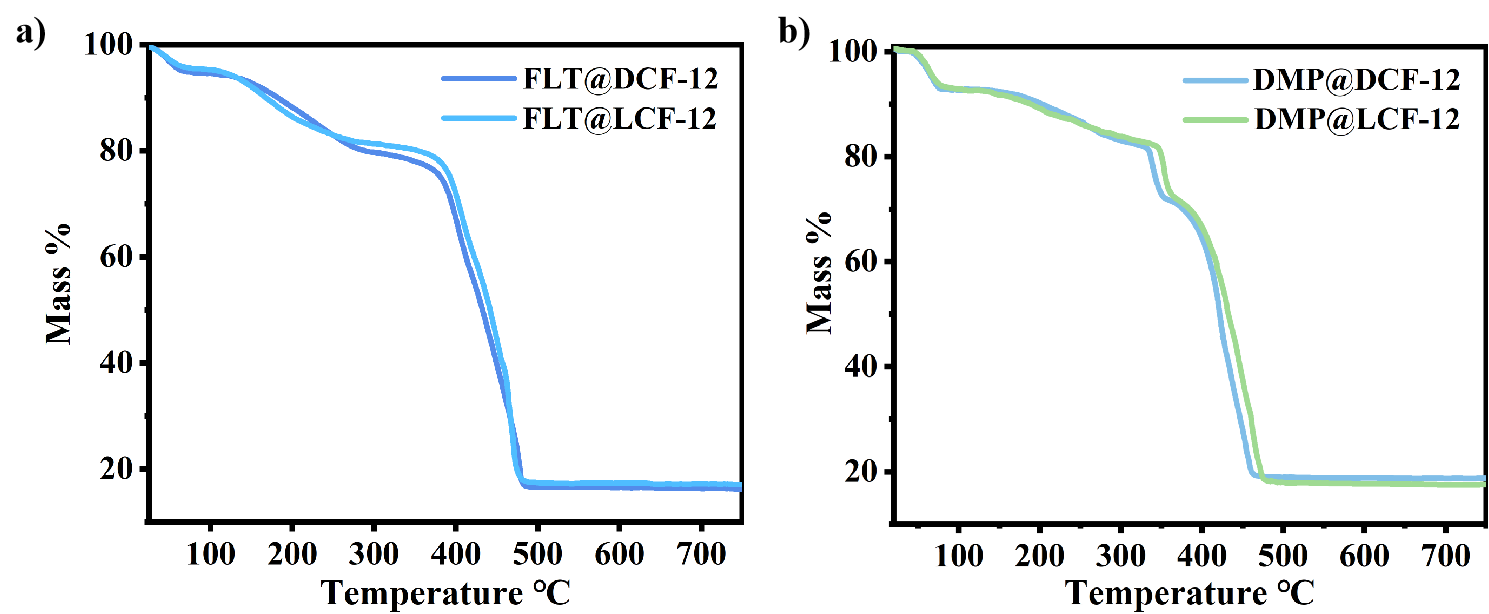


**Figure S6.** (a) The TG curves of FLT@DCF-12 and FLT@LCF-12. (b) The TG curves of DMP@DCF-12 and DMP@LCF-12.

**
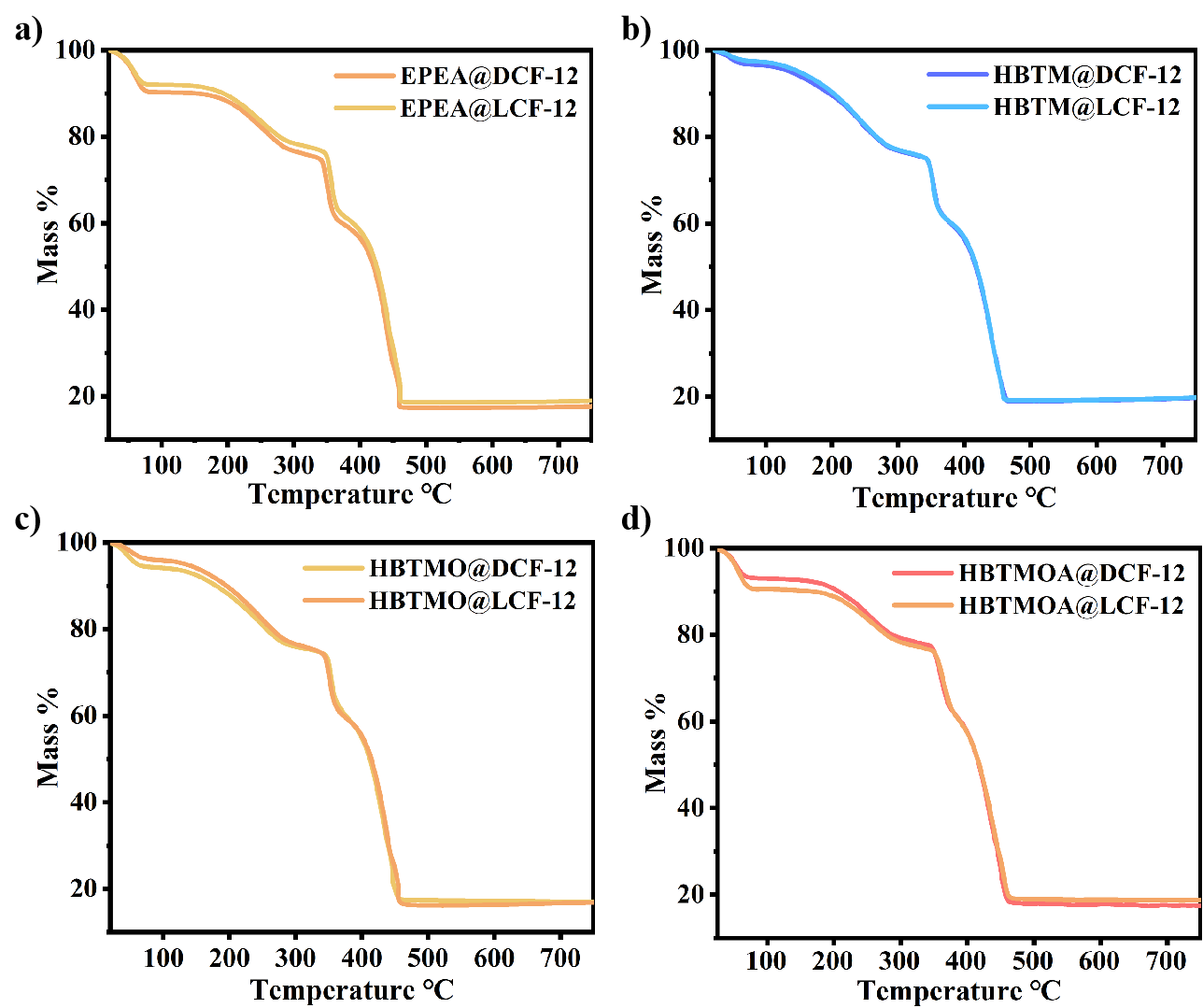
**

**Figure S7.** (a) The TG curves of EPEA@DCF-12 and EPEA@LCF-12. (b) The TG curves of HBTM@DCF-12 and HBTM@LCF-12. (c) The TG curves of HBTMO@DCF-12 and HBTMO@LCF-12. (d) The TG curves of HBTMOA@DCF-12 and HBTMOA@LCF-12.

**
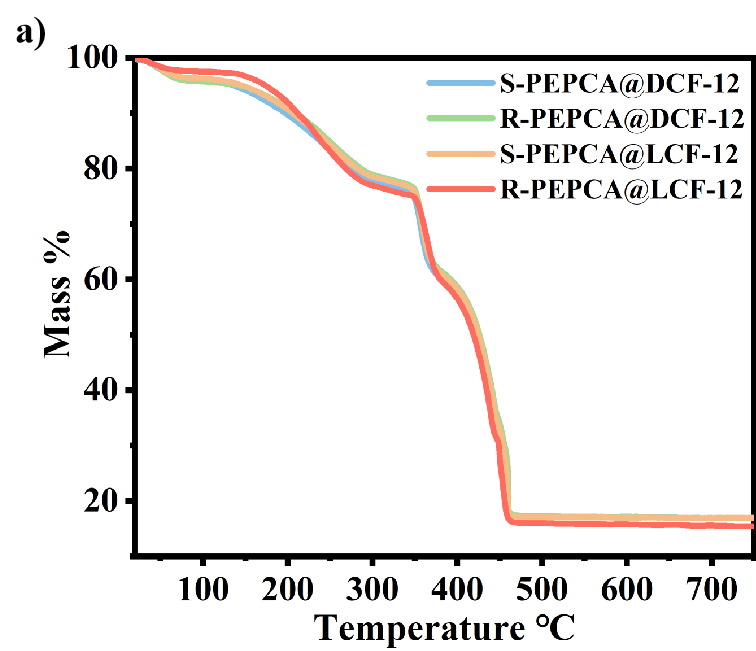
**

**Figure S8.** The TG curves of S-PEPCA@DCF-12, R-PEPCA@DCF-12, S-PEPCA@LCF-12 and R-PEPCA@LCF-12.

1. **Photophysical properties of guests and Guests@MOFs**

**
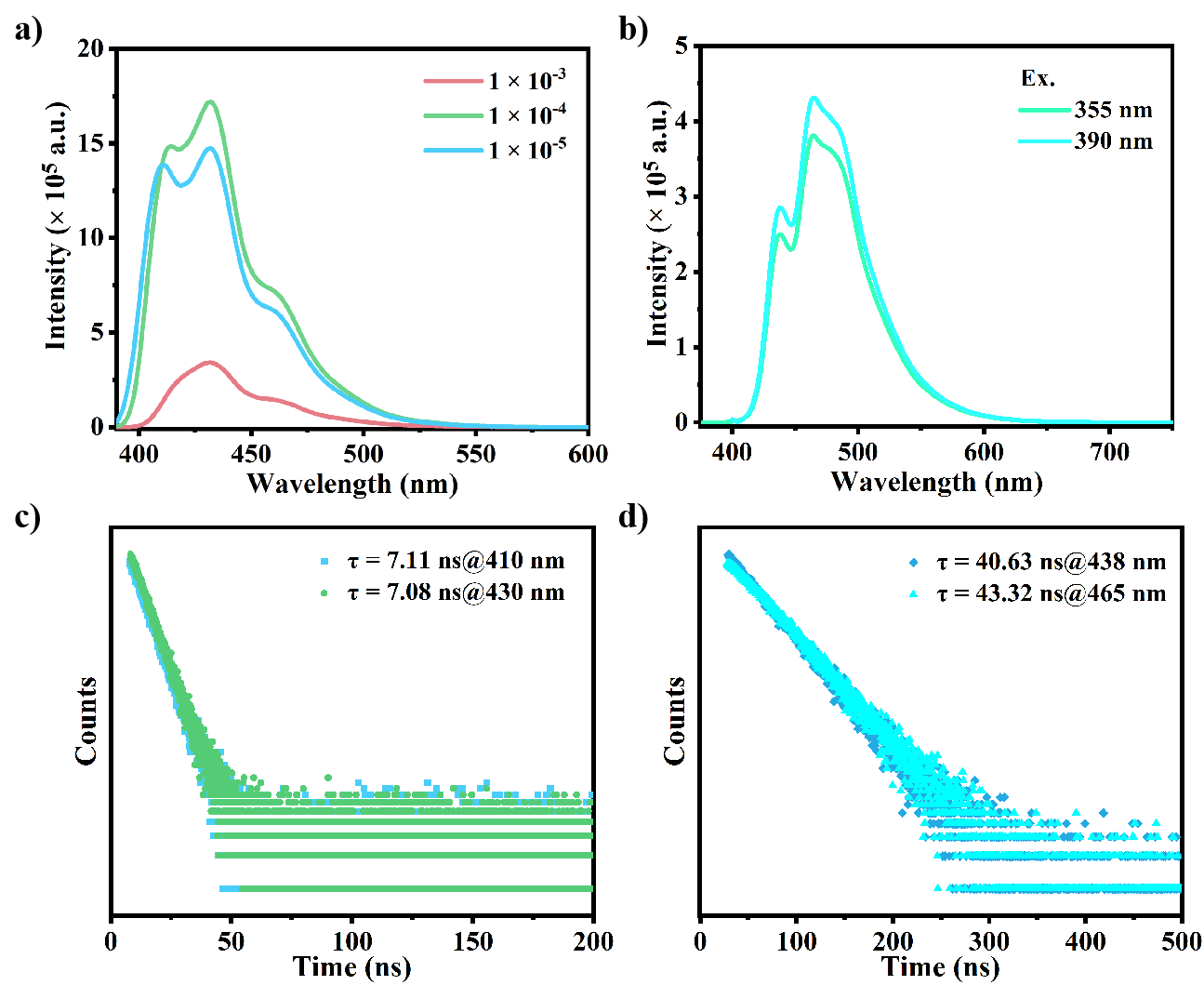
**

**Figure S9.** Fluorescence behaviors of DMA solution of FLT at different concentrations from 1 × 10^‒5^ to 1 × 10^‒3^ mol/L (a) and solid (b). Fluorescence lifetime decay profiles of FLT at 1 × 10^‒4^ mol/L (c) and solid (d) under ambient conditions.

**
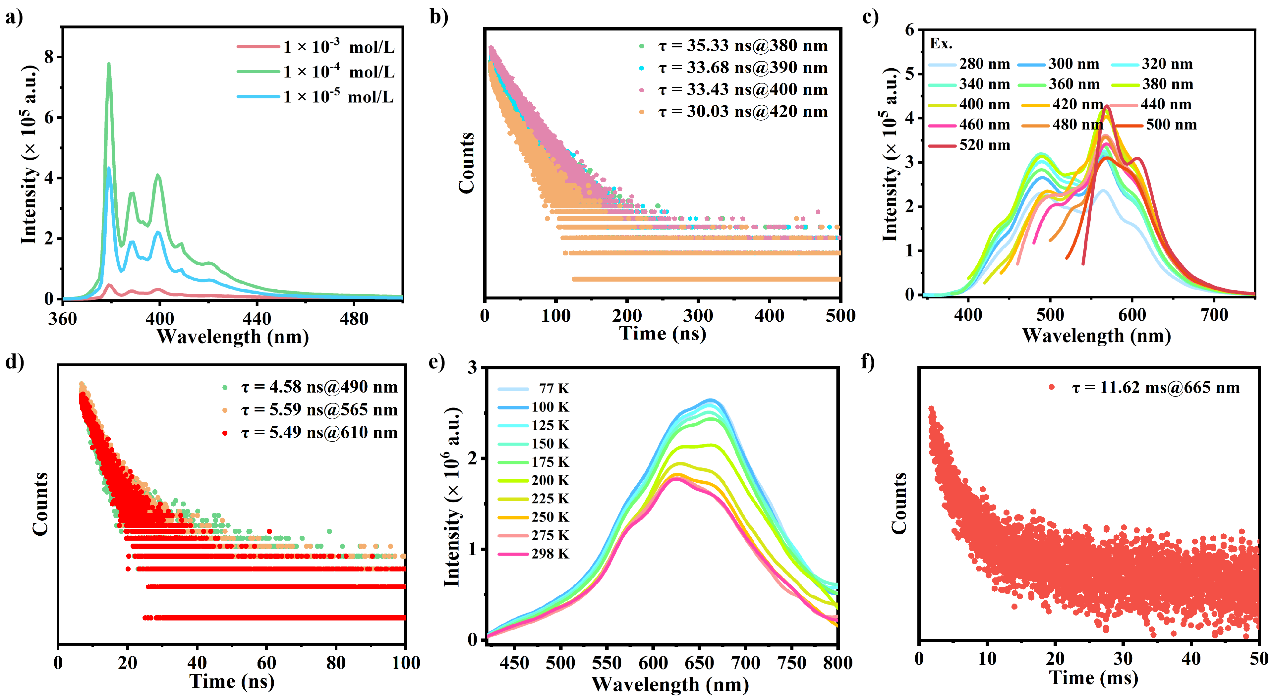
**

**Figure** **S10.** (a) The emission of DMP in DMA solution at different concentrations from 1 × 10^‒5^ to 1 × 10^‒3^ mol/L. (b) Fluorescence lifetime decay profiles of DMP at 1 × 10^‒4^ mol/L. (c) The solid-state emission of DMP at room temperature under different excitation wavelengths from 280 to 520 nm. (d) Fluorescence lifetime decay profiles of DMP at solid-state. (e) Temperature-dependent afterglow spectra for DMP with a delayed time of 0.5 ms from 77 to 298 K. (f) Time-resolved decay curves for emission of DMP at 665 nm at 77 K.


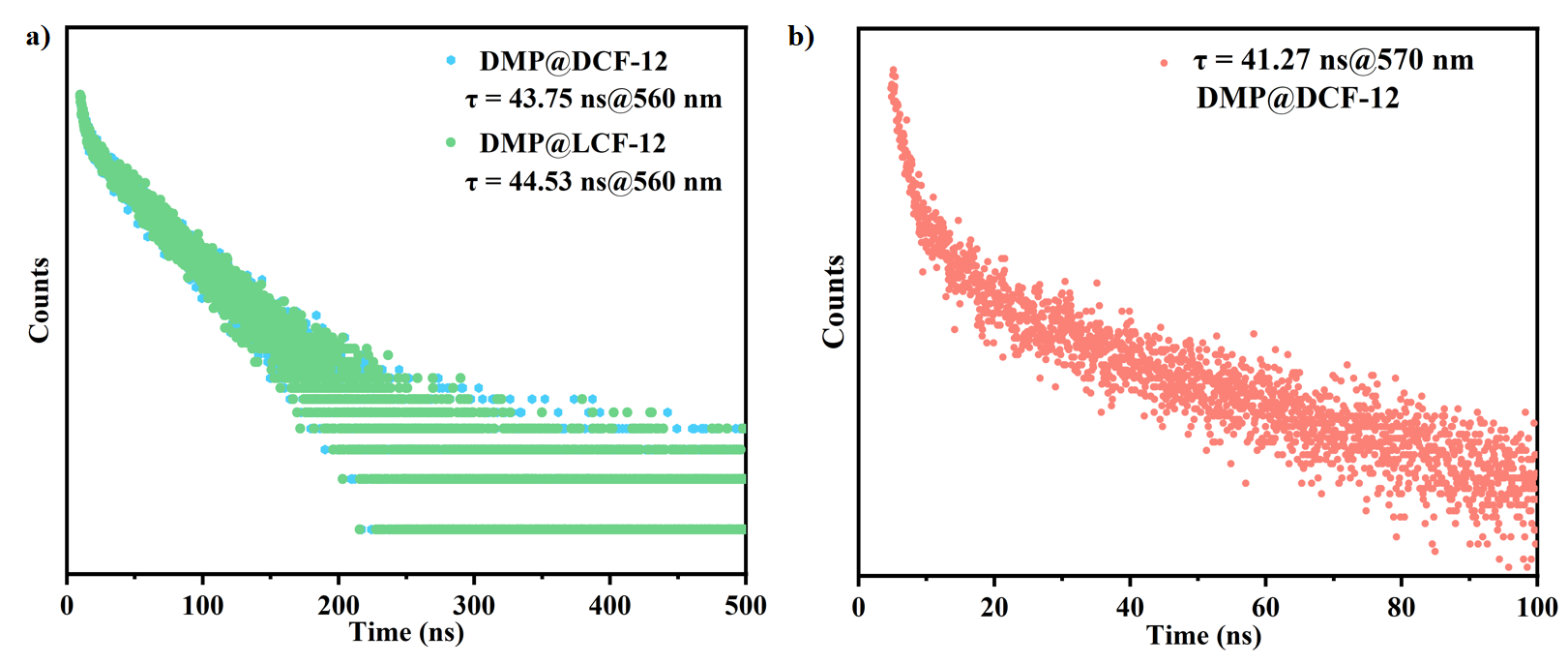


**Figure S11.** (a) Fluorescence lifetime decay profiles of DMP@DCF-12 DMP@LCF-12 at 560 nm under ambient conditions. (b) Fluorescence lifetime decay profiles of DMP@DCF-12 at 570 nm under ambient conditions.


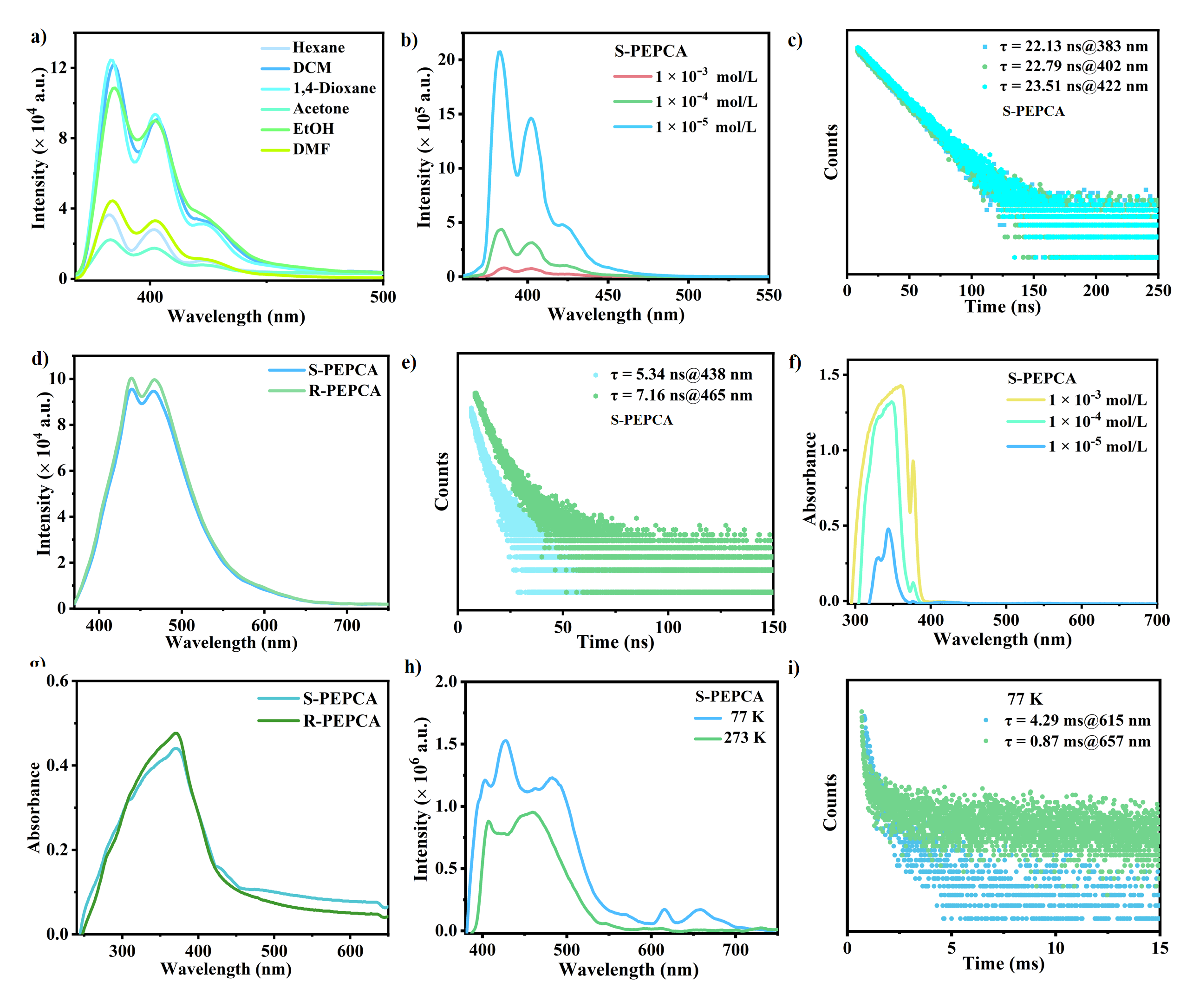


**Figure S12.** (a) The emission of S-PEPCA in different solvents at 1 × 10^‒3^ mol/L. (b) The emission of S-PEPCA in DMA solution at different concentrations from 1 × 10^‒5^ to 1 × 10^‒3^ mol/L. (c) Fluorescence lifetime decay profiles of S-PEPCA at 1 × 10^‒4^ mol/L. (d) The solid-state emission of R-PEPCA and S-PEPCA at room temperature. (e) Fluorescence lifetime decay profiles of S-PEPCA at solid-state. (f) The absorbance in DMA solution of S-PEPCA at different concentrations from 1 × 10^‒5^ to 1 × 10^‒3^ mol/L. (g) The solid-state absorption of R-PEPCA and S-PEPCA. (h) The emission of S-PEPCA in PVA film at 77 and 298 K with a delayed time of 0.5 ms. (i) Time-resolved decay curves for emission at 615 and 657 nm at 77 K with a delayed time of 0.5 ms.


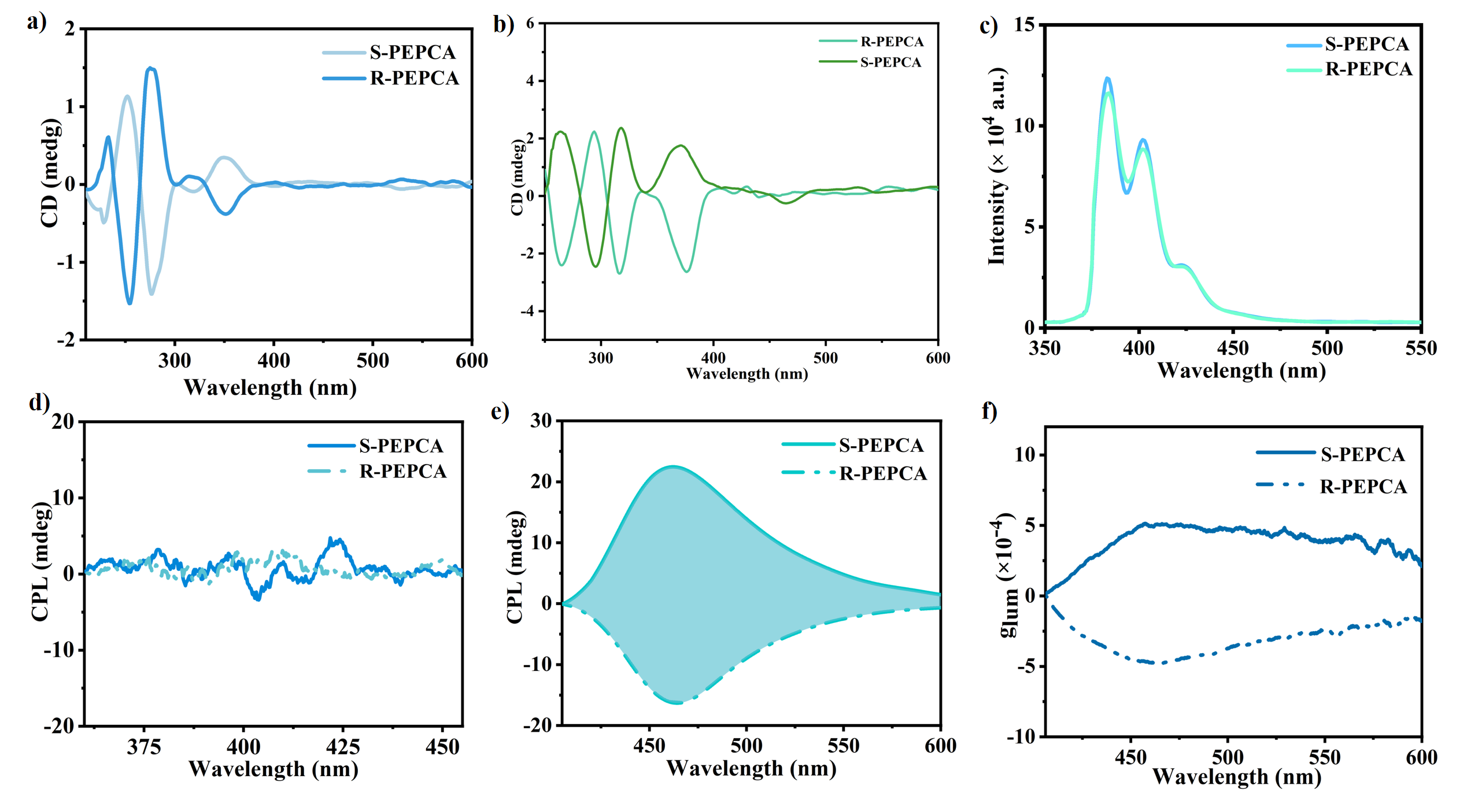


**Figure S13.** (a) CD spectra of R-PEPCA and S-PEPCA in 10^‒5^ mol/L DMF. (b) CD spectra of R-PEPCA and S-PEPCA in solid-state. (c) The emission of R-PEPCA and S-PEPCA in 10^‒3^ mol/L DMF. (d) CPL spectra of R-PEPCA and S-PEPCA in 10^‒3^ mol/L DMF. (e) CPL spectra of R-PEPCA and S-PEPCA in solid-state. (f) The *g*_lum_ values of R-PEPCA and S-PEPCA in solid-state.

**
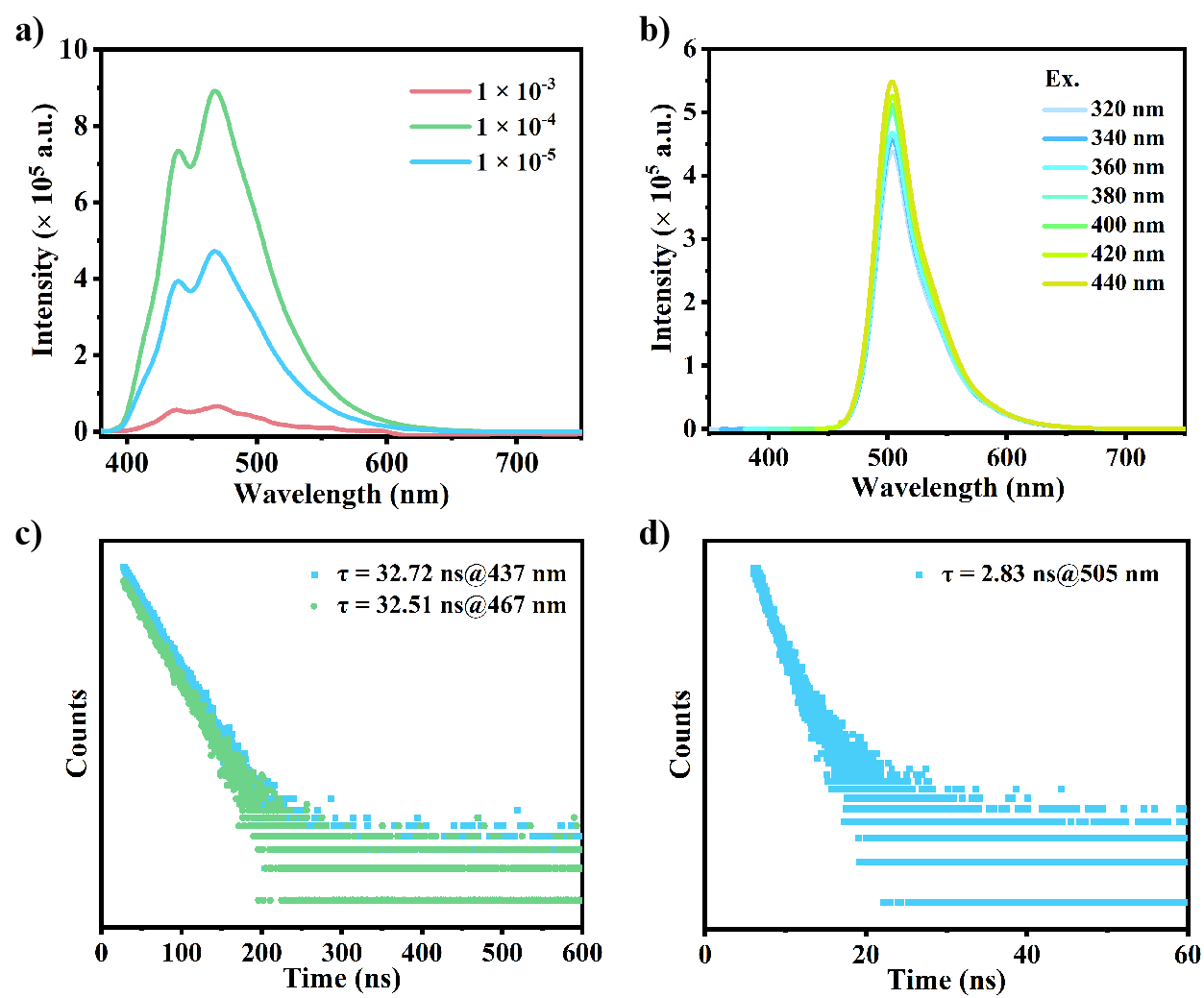
**

**Figure S14.** Fluorescence behaviors of DMA solution of EPEA at different concentrations from 1 × 10^‒5^ to 1 × 10^‒3^ mol/L (a) and solid (b). Fluorescence lifetime decay profiles of EPEA at 1 × 10^‒4^ mol/L (c) and solid (d) under ambient conditions.

**
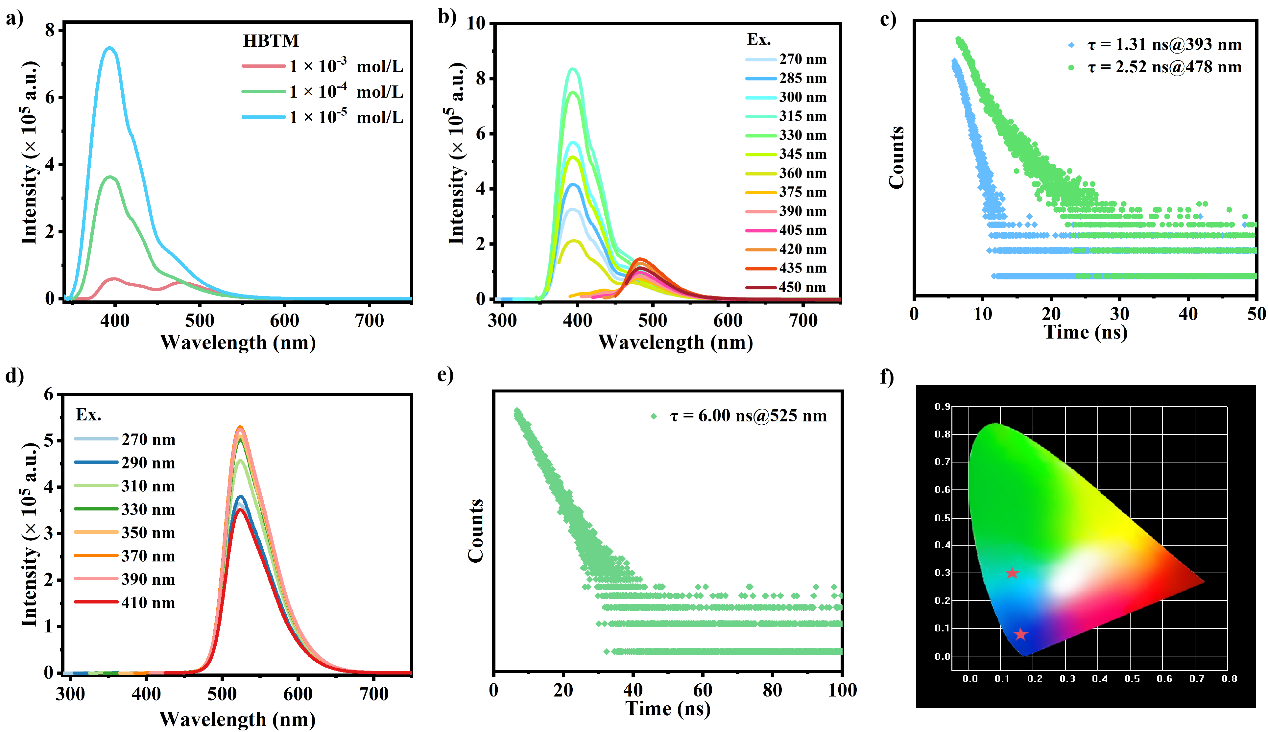
**

**Figure S15.** (a) Fluorescence behaviors of DMA solution of HBTM at different concentrations from 1 × 10^‒5^ to 1 × 10^‒3^ mol/L (b) The prompt emission of HBTM in DMA (1 × 10^‒4^ mol/L) under different excitation wavelengths from 270 to 450 nm. (c) Fluorescence lifetime decay profiles of HBTM at 1 × 10^‒4^ mol/L. (d) The prompt emission of HBTM (solid) under different excitation wavelengths changing from 270 to 410 nm. (e) Fluorescence lifetime decay profiles of HBTM at solid-state. (f) CIE coordinate diagram of the luminescence spectra of HBTM at 1 × 10^‒4^ mol/L with different excitation wavelengths at 330 and 390 nm.


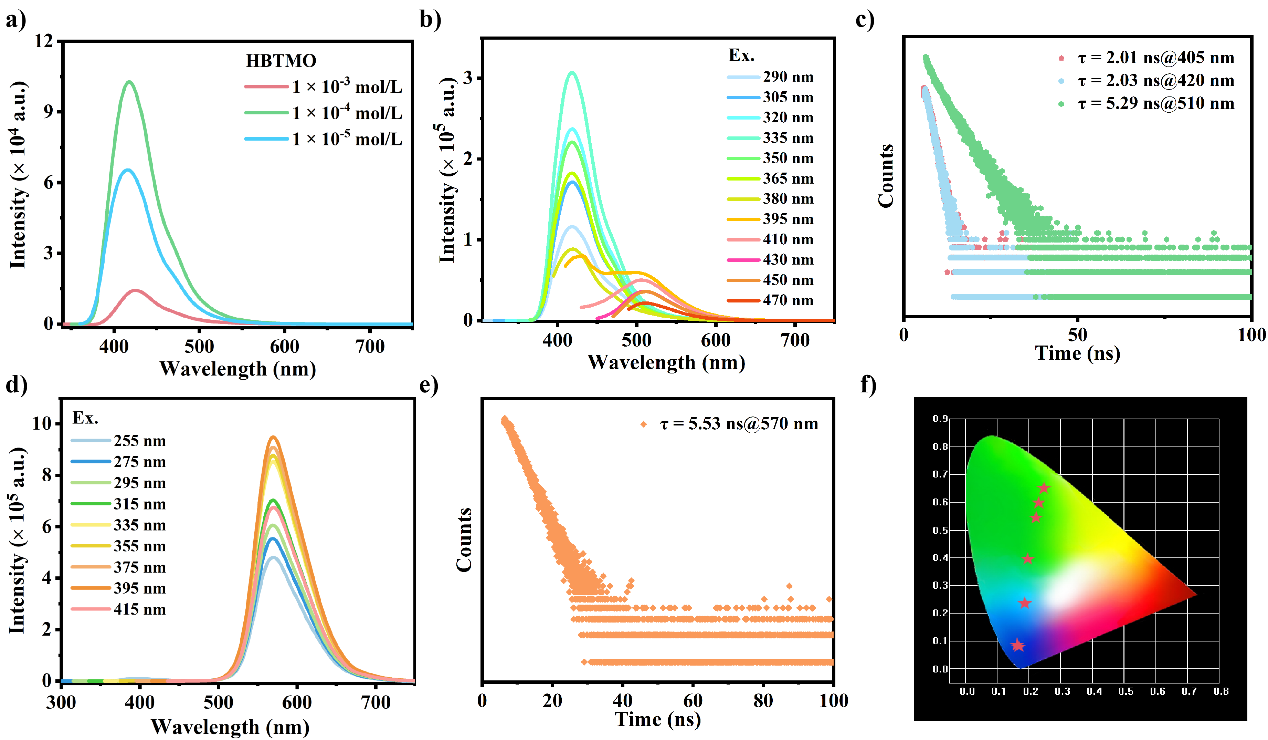


**Figure S16.** (a) Fluorescence behaviors of DMA solution of HBTMO at different concentrations from 1 × 10^‒5^ to 1 × 10^‒3^ mol/L. (b) The prompt emission of HBTMO (1 × 10^‒4^ mol/L in DMA) under different excitation wavelengths. (c) Fluorescence lifetime decay profiles of HBTMO at 1 × 10^‒4^ mol/L. (d) The prompt emission of HBTMO (solid) under different excitation wavelengths changes from 255 to 415 nm. (e) Fluorescence lifetime decay profiles of HBTMO at solid. (f) CIE coordinate diagram of the luminescence spectra of HBTMO at 1 × 10^‒4^ mol/L with the excitation wavelengths changing from 365 to 470 nm.

**
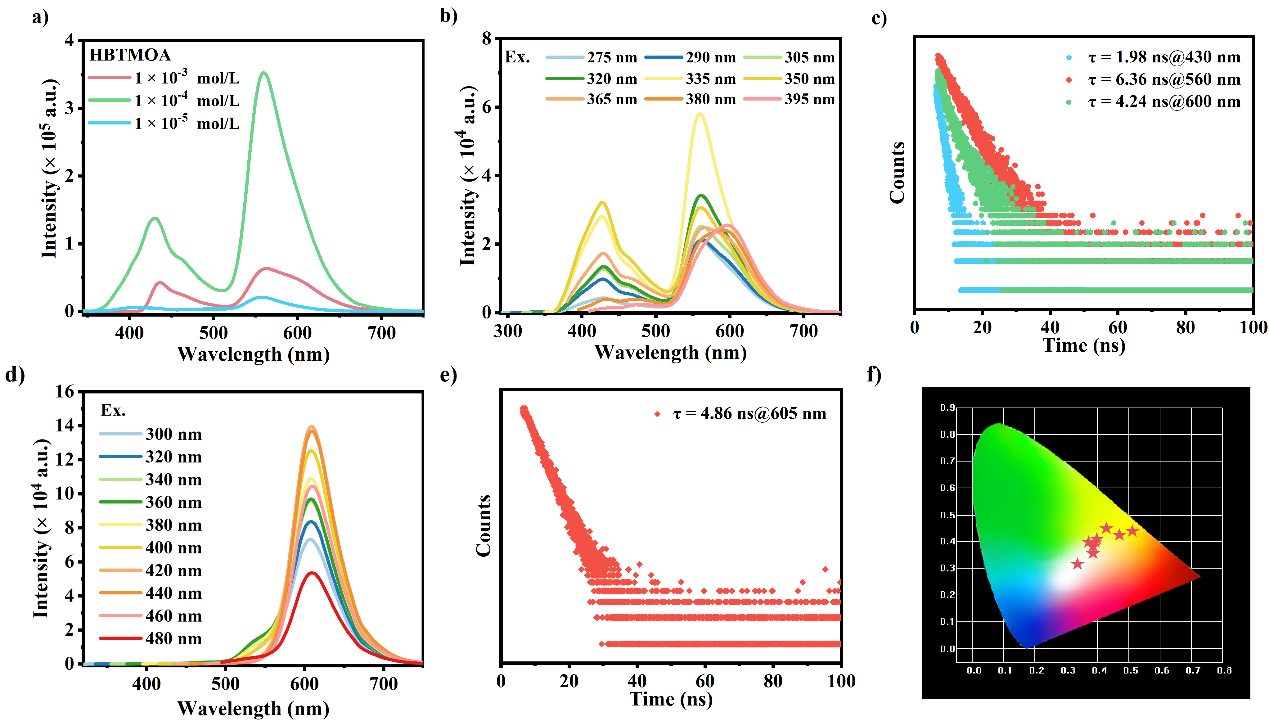
**

**Figure S17.** (a) Fluorescence behaviors of DMA solution of HBTMOA at different concentrations from 1 × 10^‒5^ to 1 × 10^‒3^ mol/L. (b) The prompt emission of HBTMOA (1 × 10^‒4^ mol/L in DMA) under different excitation wavelengths. (c) Fluorescence lifetime decay profiles of HBTMOA at 1 × 10^‒4^ mol/L. (d) The prompt emission of HBTMOA (solid) under different excitation wavelengths changing from 300 to 480 nm. (e) Fluorescence lifetime decay profiles of HBTMOA at solid. (f) CIE coordinate diagram of the luminescence spectra of HBTMOA at 1 × 10^‒4^ mol/L. with the excitation wavelengths changing from 275 to 395 nm.

**
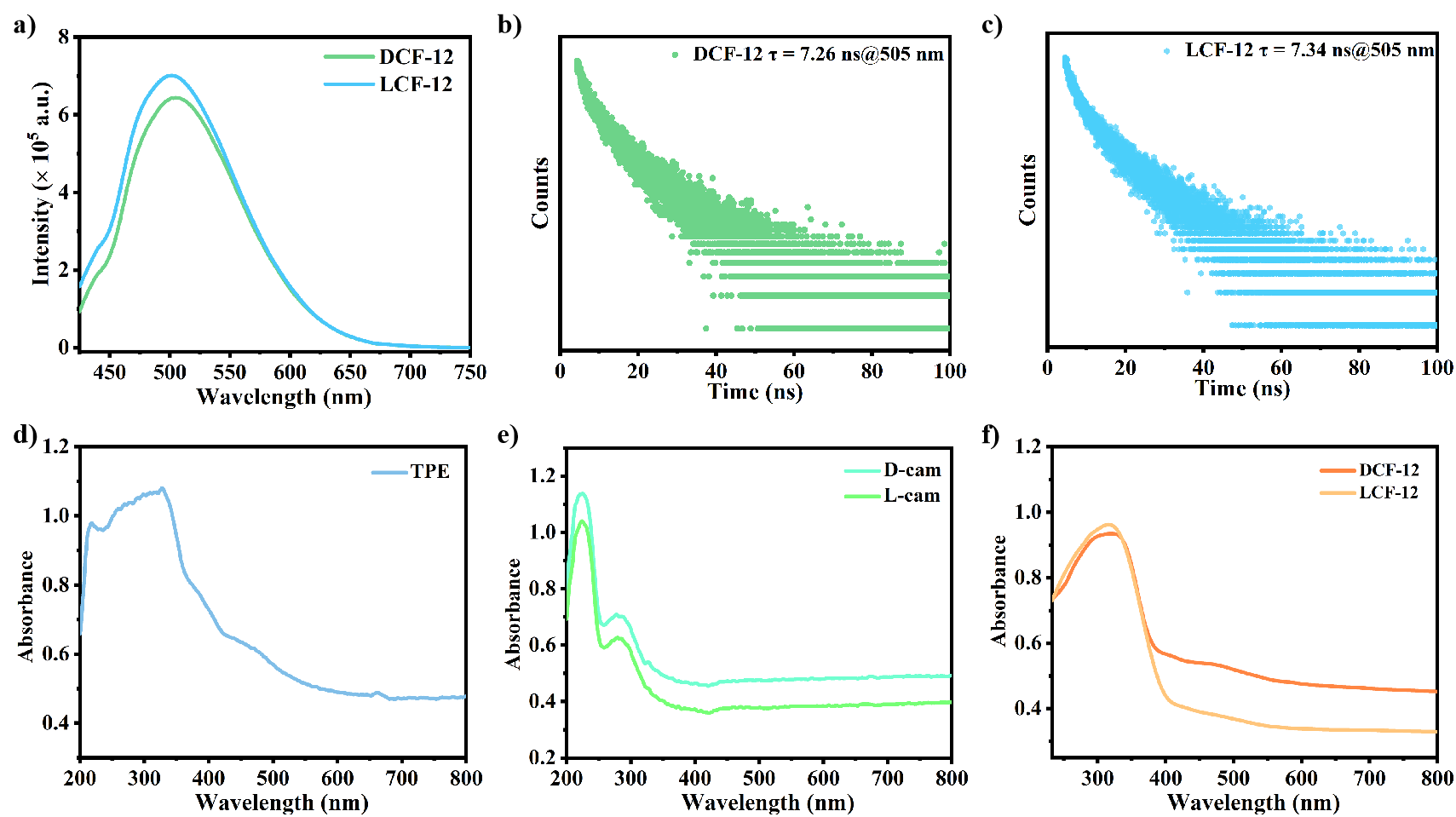
**

**Figure S18.** (a) The luminescence emission of DCF-12 and LCF-12 upon excitation wavelengths of 400 nm at ambient conditions. (b) Fluorescence lifetime decay profiles of DCF-12 at solid state. (c) Fluorescence lifetime decay profiles of LCF-12 at solid. (d) The solid-state absorption of TPE. (e) The solid-state absorption of D-cam and L-cam. (f) The solid-state absorption of DCF-12 and LCF-12.

**
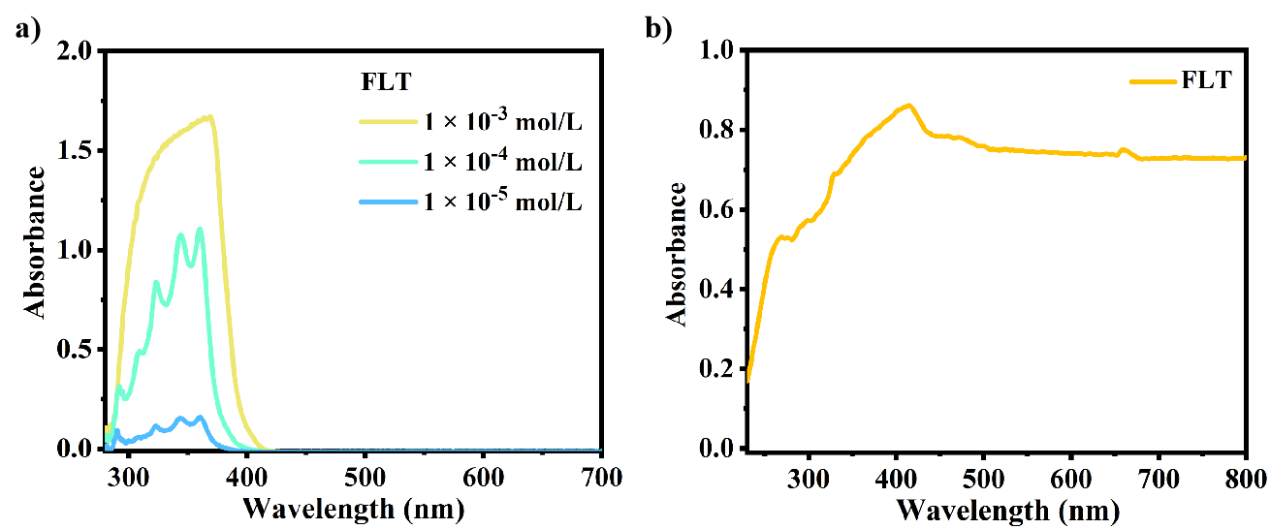
**

**Figure S19.** The absorbance in DMA solution of FLT at different concentrations from 1 × 10^‒5^ to 1 × 10^‒3^ mol/L (a) and solid-state (b).

**
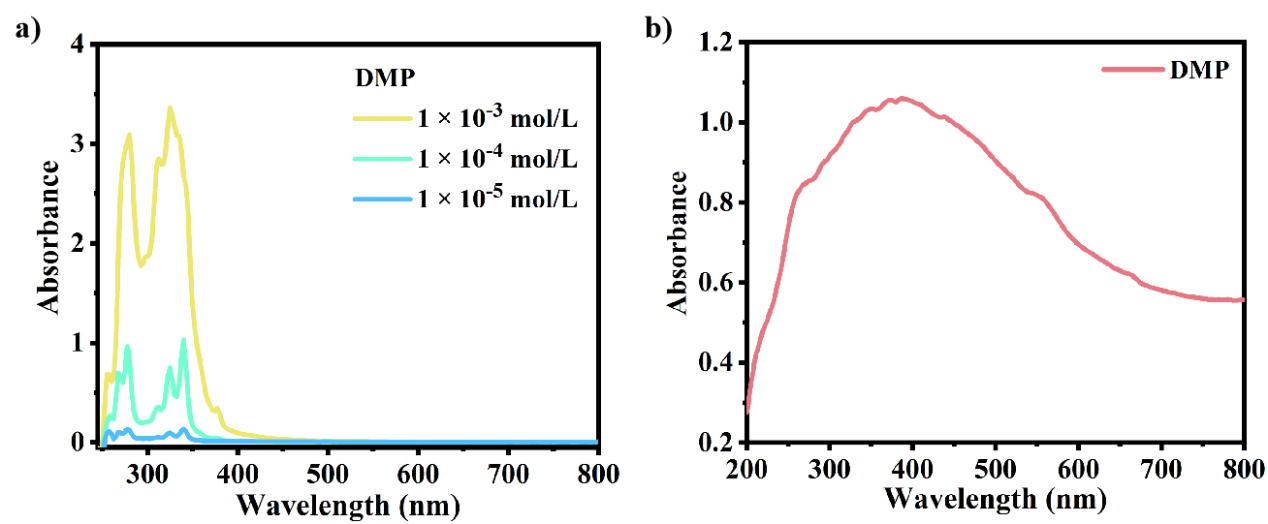
**

**Figure S20.** The absorbance in DMA solution of DMP at different concentrations from 1 × 10^‒5^ to 1 × 10^‒3^ mol/L (a) and solid-state (b).


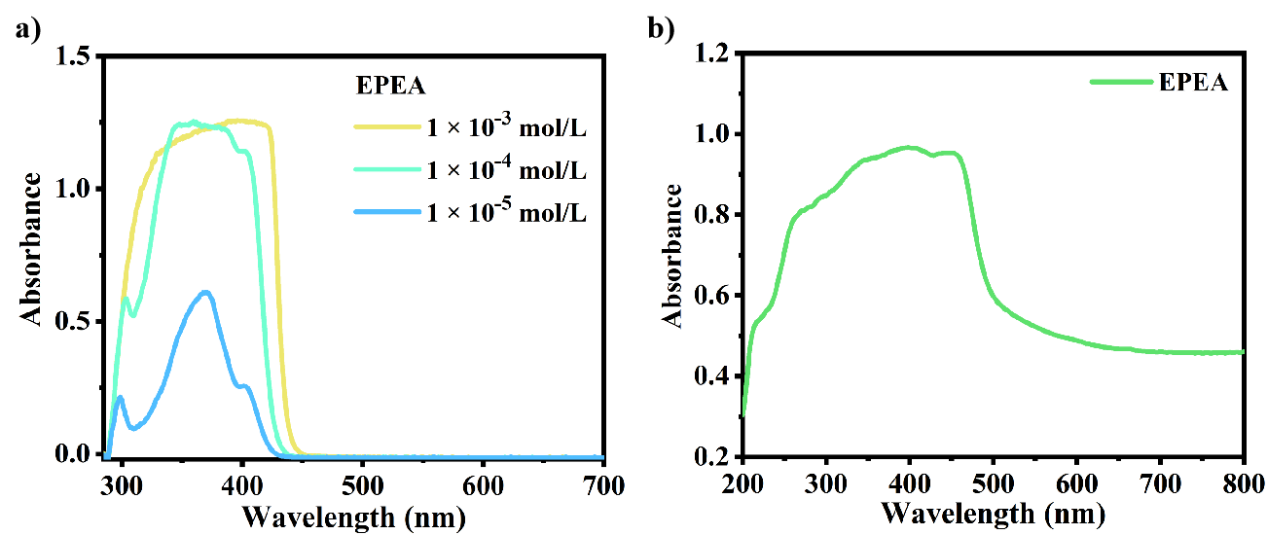


**Figure S21.** The absorbance in DMA solution of EPEA at different concentrations from 1 × 10^‒5^ to 1 × 10^‒3^ mol/L (a) and solid-state (b).


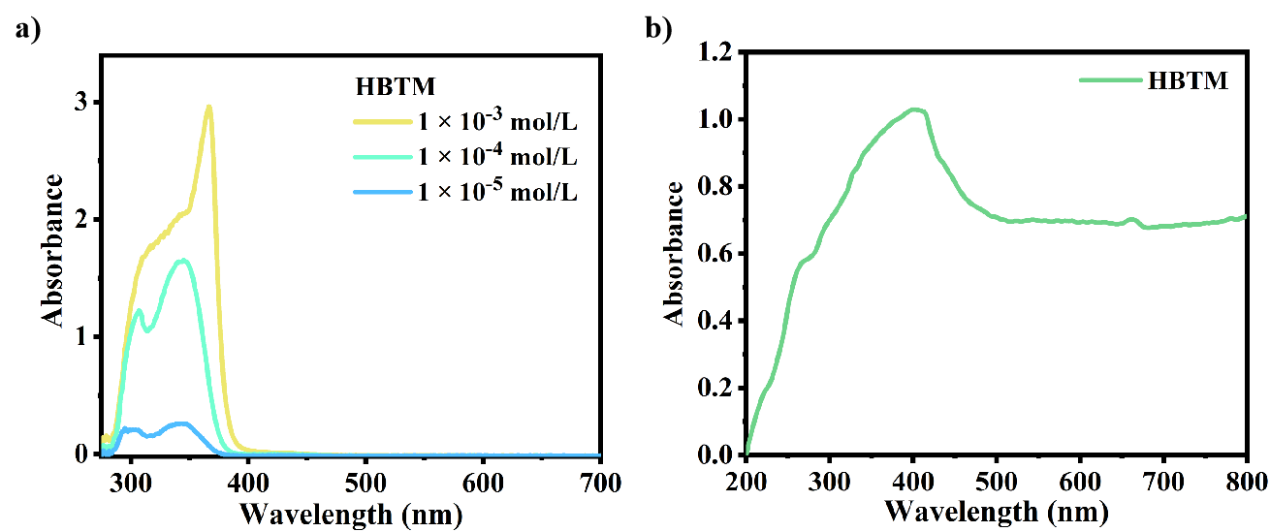


**Figure S22.** The absorbance in DMA solution of HBTM at different concentrations from 1 × 10^‒5^ to 1 × 10^‒3^ mol/L (a) and solid-state (b).


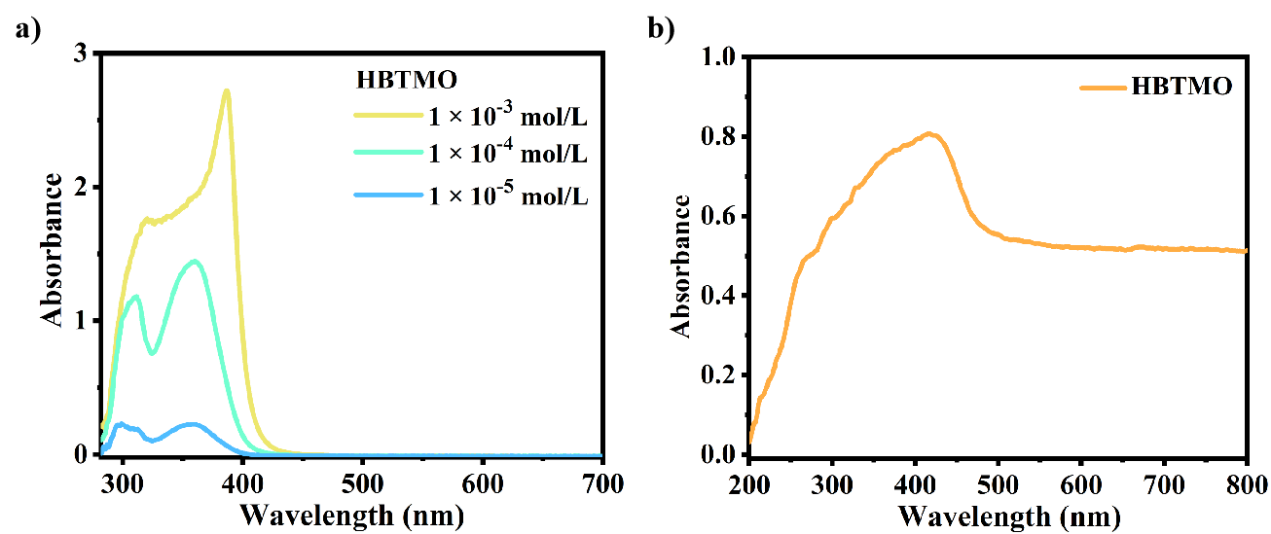


**Figure S23.** The absorbance in DMA solution of HBTMO at different concentrations from 1 × 10^‒5^ to 1 × 10^‒3^ mol/L (a) and solid-state (b).


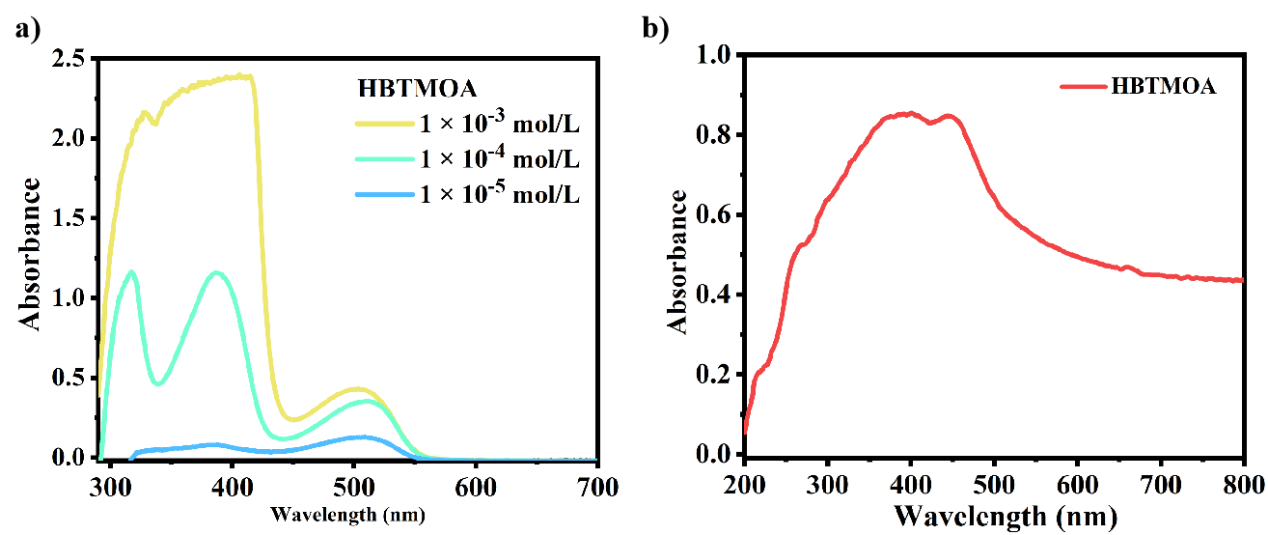


**Figure S24.** The absorbance in DMA solution of HBTMOA at different concentrations from 1 × 10^‒5^ to 1 × 10^‒3^ mol/L (a) and solid-state (b).


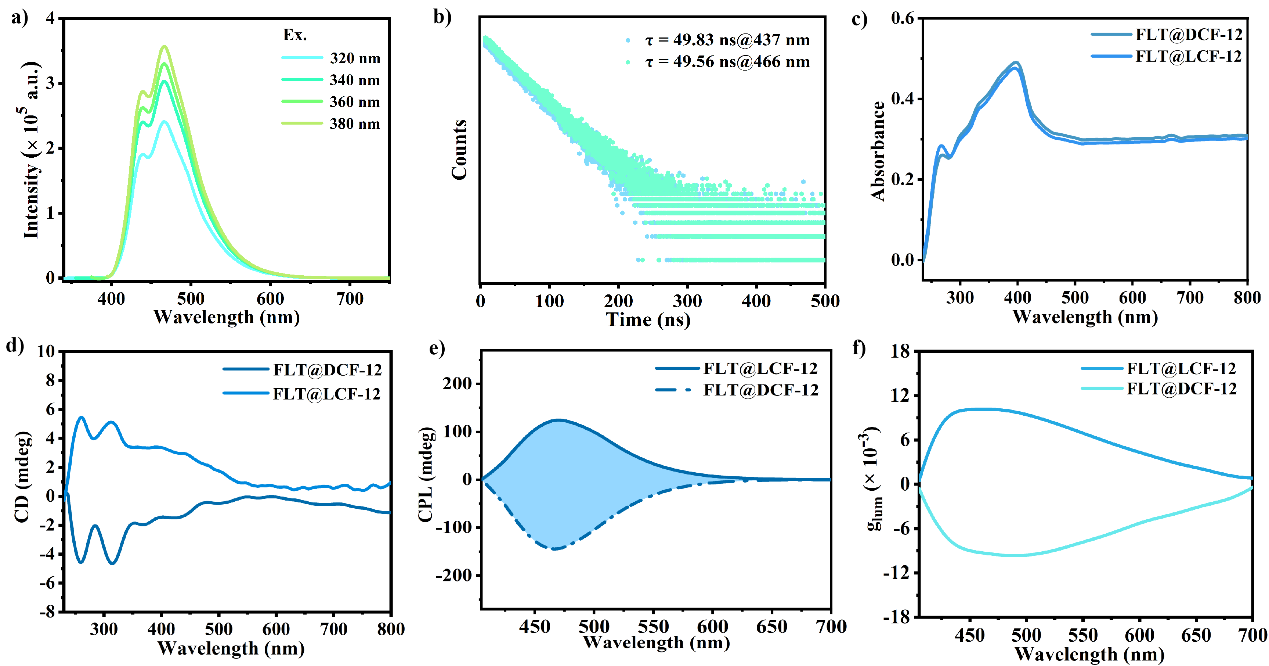


**Figure S25.** (a) The prompt emission of FLT@DCF-12 under different excitation wavelengths. (b) The lifetime of FLT@DCF-12 at 437 and 466 nm, respectively. (c) The solid-state absorption of FLT@DCF-12 and FLT@LCF-12. (d) CD spectra of FLT@DCF-12 and FLT@LCF-12. (e) CPL spectra of FLT@DCF-12 and FLT@LCF-12. (f) The *g*_lum_ values of FLT@DCF-12 and FLT@LCF-12.

**
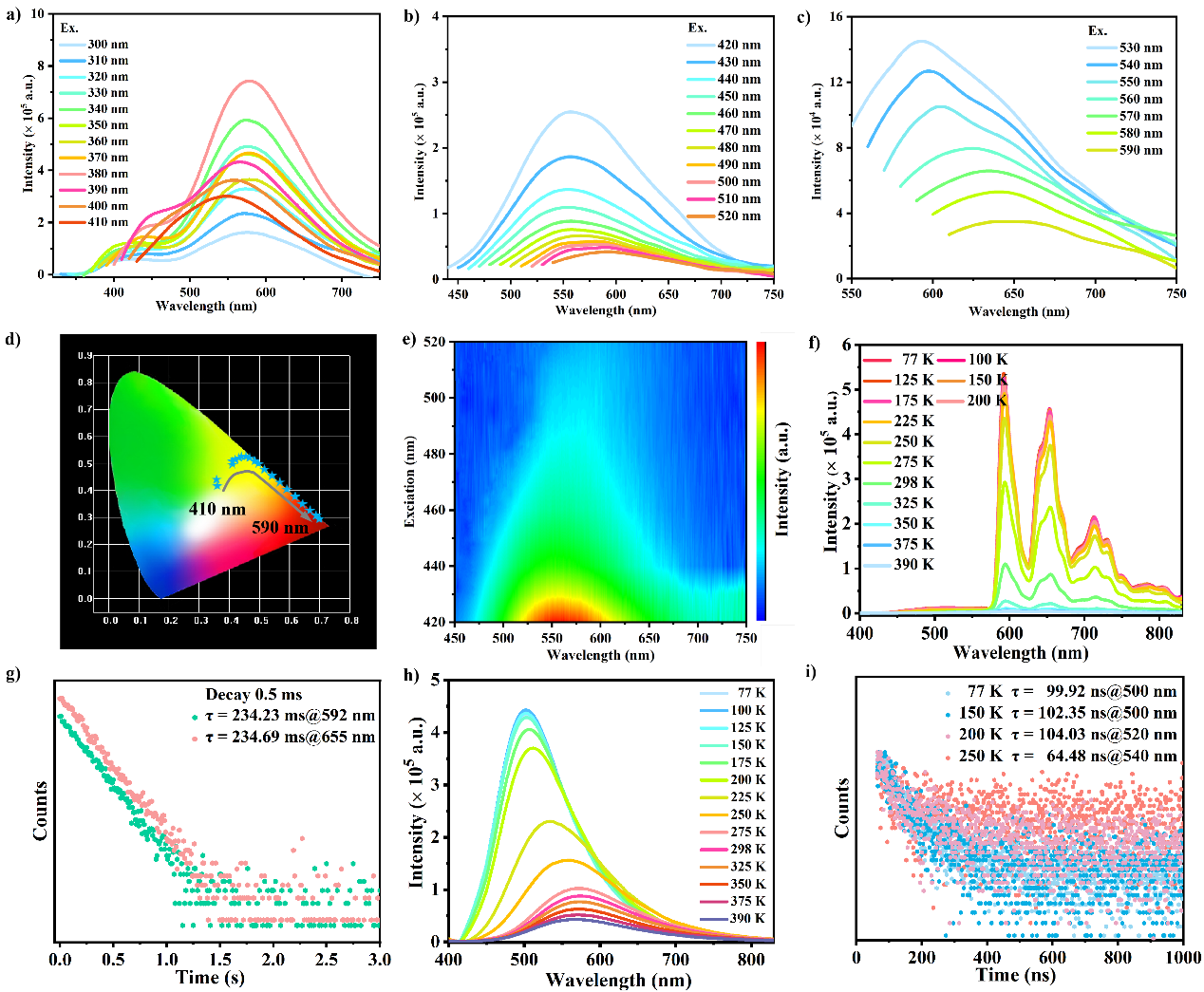
**

**Figure S26.** (a) The luminescence emission of DMP@DCF-12 under different excitation wavelengths from 300 to 410 nm. (b) The luminescence emission of DMP@DCF-12 under different excitation wavelengths from 420 to 520 nm. (c) The luminescence emission of DMP@DCF-12 under different excitation wavelengths from 530 to 590 nm. (d) CIE coordinate diagram of the luminescence spectra of DMP@DCF-12 with the excitation wavelengths changing from 410 to 590 nm. (e) Excitation-phosphorescence mapping of the DMP@DCF-12 under ambient conditions with different excitation wavelengths from 420 to 520 nm. (f) Temperature-dependent afterglow spectra for DMP@DCF-12 with a delayed time of 0.5 ms from 77 to 390 K. (g) Time-resolved decay curves for emission at 592 and 655 nm at 77 K with a delayed time of 0.5 ms. (h) Temperature-dependent luminescence emission spectra for DMP@DCF-12 from 77 to 390 K. (i) The prompt lifetime at 500, 520 540 nm under different temperatures.


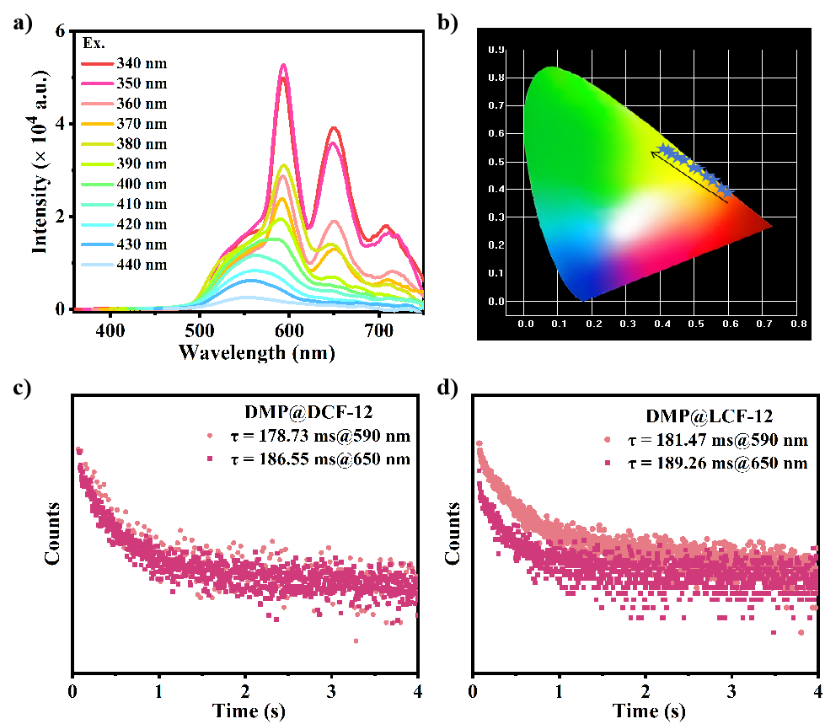


**Figure S27.** (a) The photoluminescence spectra of DMP@DCF-12 under different excitation wavelengths from 340 to 440 nm with a delayed time of 0.5 ms. (b) CIE coordinate diagram of the photoluminescence spectra of DMP@DCF-12 with the excitation wavelengths changing from 290 to 390 nm with a delayed time of 0.5 ms. (c) and (d) Time-resolved decay curves for emission of DMP@DCF-12 and DMP@LCF-12 at 590 and 650 nm with a delayed time of 0.5 ms under ambient conditions.


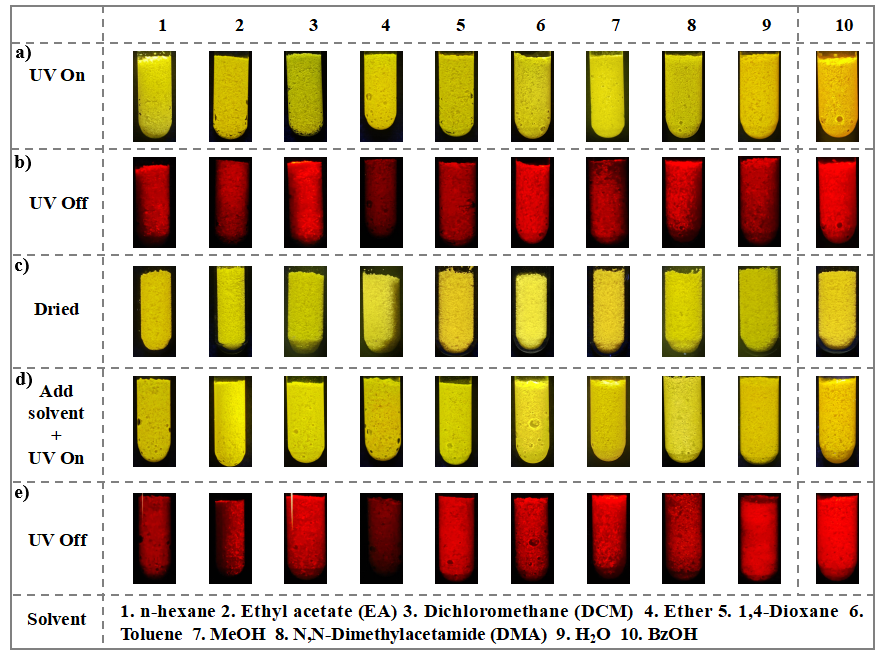


**Figure S28** (a) The visible luminescence of the solvent-activated samples under UV light. (b) The visible afterglow of the solvent-activated samples. (c) The solvent-activated samples were dried under 120 °C for 5 hours. (d) The visible luminescence of the solvent-activated samples was recovered under UV light. (e) The similar visible afterglow of the solvent-activated samples could be observed again. The single crystal sample was treated by n-hexane, EA, DCM, Ether, 1,4-dioxane, MeOH, DMA, H_2_O and BzOH, respectively.


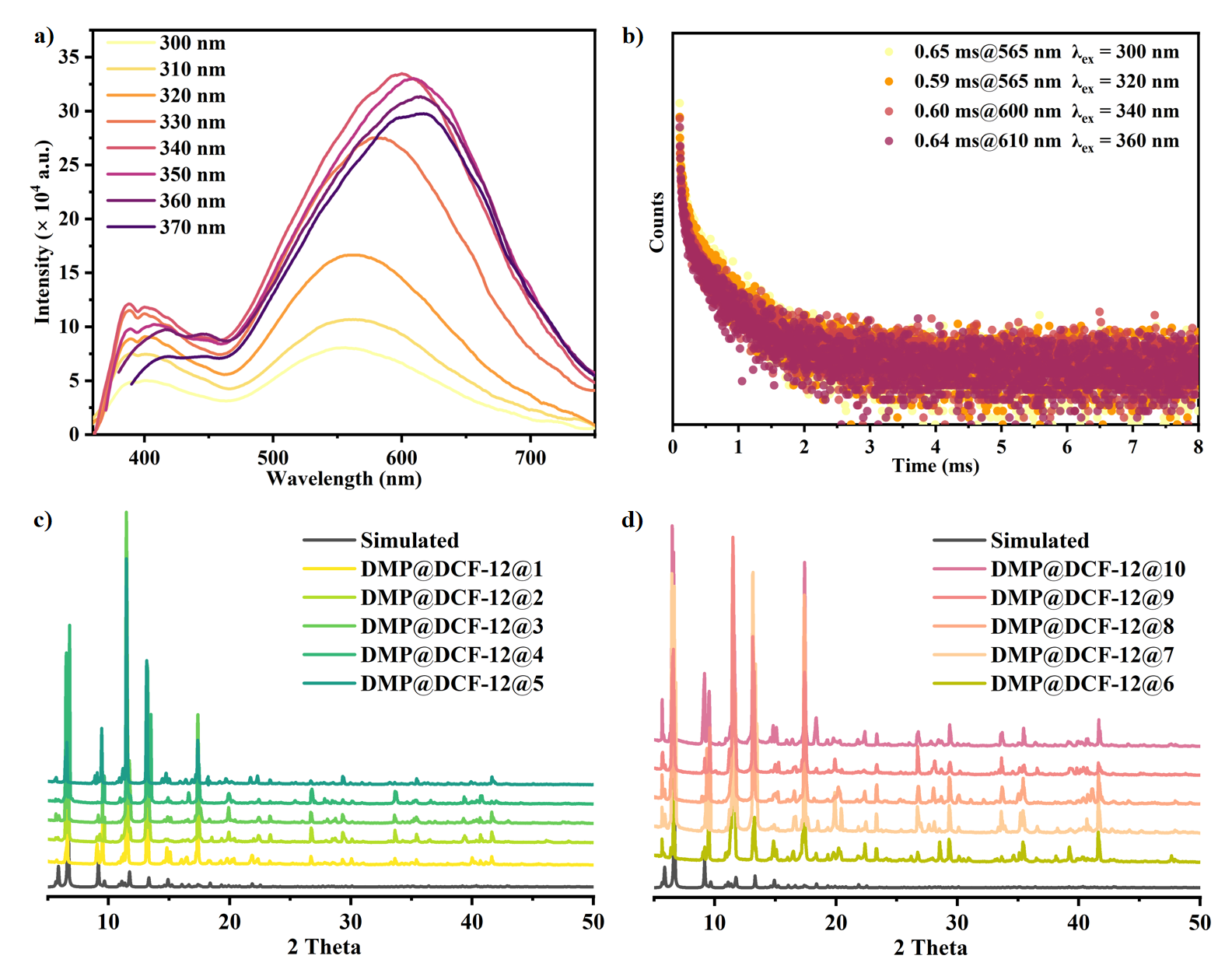


**Figure S29** (a) The phosphorescence spectra of DMP@DCF-12 after being heated under 120 ℃ for 5 hours. (b) Time-resolved decay curves for emission at 565, 565, 600 and 610 nm upon excitation at 300, 320 340 and 360 nm under ambient temperature. (c,d) PXRD patterns of DMP@DCF-12 after the cycling experiment.

In this manuscript, it has demonstrated that DMP@DCF-12 can exhibit remarkable red phosphorescence with the assistance of BzOH, and the phosphorescence intensity significantly decreased in the absence of BzOH after being heated at 120 °C for 5 h. Then the photophysical property of DMP@DCF-12 without BzOH should be measured. As shown in Figure S29a and S29b, it can find that the phosphorescence emission intensity of the dried DMP@DCF-12 between 350 to 750 nm become weaker, and the phosphorescence delayed dramatically decreased. In order to evaluate the solvent-responsive behaviors towards RTP of DMP@DCF-12, other nine kinds of organic solvents were selected as triggers. As shown in Figure S28, the dried DMP@DCF-12 crystals were soaked into the above solvents for 2 hours, the solvent-activated samples all emit yellow light with slight differences under 365 nm UV lamp. Unexpectedly, all solvent-activated samples show the naked-eye red afterglow after removing the UV irradiation sources. The reversible cyclicity was also investigated. The solvent-soaked samples were dried at 120 °C for 5 hours again, then the samples were immersed into these ten kinds of solvents, it can be found that the solvent-activated samples also show the corresponding fluorescence and red afterglow under UV irradiation and after turning off the excitation. The similar phenomenon was also could be observed after eight successive recycles. Meanwhile, the stability of solvent-activated samples was checked, the high stability and phase purification can be confirmed by PXRD (Figure S29). We also obtained the accurate single crystal structures of DMP@DCF-12 by single-crystal X-ray diffraction, the host frameworks of DMP@DCF-12 have no changes before and after solvent treatment, indicating that the chiral configuration of host framework can keep well.

In addition, many pyrene-cored molecules have been demonstrated to have the phosphorescence property, it should be noted that the phosphorescence performance of these molecules is very weak under ambient conditions, the phosphorescence intensity can be detected under low temperature, therefore, in order to achieve RTP of these pyrene-cored molecules, the multi-component systems were prepared through host–guest doping or cocrystals. The triplet energy level of the host assists the guest excitons to undergo intersystem crossing in the phosphorescence process. Besides, the maximum excitation wavelength of pyrene appears red shift in host-guest compound than itself, indicating that the excitons of pyrene molecules could be promoted with the aid of triplet state-triplet state energy transfer between host and guest emitters, further facilitating the generation of phosphorescence.^33^ For instance, the confinement of guest molecule 1 within “pyrene box” PTSK{1} and PTSG{1} cages leads to an efficient deep-red to NIR phosphorescence emission.^1^ Ding and co-workers prepared a series of Py/BPO (BPO = benzophenone, Py = anisole or dimethylaniline groups decorated pyrene) guest–host materials with different guest–host molar ratios.^2^ The phosphorescence of guests was dramatically enhanced through the host-guest doped approach. The comprehensive analysis indicated that the guests should have sufficient conjugation to reduce the T_1_ level, and the host matrix assists the guest molecules in exciton transfer and inhibits the non-radiative transition.^2^

In this manuscript, aiming at obtaining circularly polarized room temperature phosphorescence (CP-RTP), a series of pyrene-based molecules were introduced into chiral MOFs, as expected, the efficient CP-RTP was achieved. Meanwhile, the singlet and triplet energy levels of the pyrene-based guest emitters and TPE ligand in DMP@DCF-12 were calculated. The energy gap ΔE_ST_ of the S_1_ and T_1_ states of pyrene molecule is 1.92 eV, such large ΔE_ST_ makes strong against to intersystem crossing. Nevertheless, the energy gaps between the S_1_ state of pyrene and the T_1_ state of tpe molecule is down to 0.34 eV, which is favorable to excitonic ISC (Figure 5e). The similar mechanism has been demonstrated in some host–guest doped systems.^3-5^

Totally, DMP@DCF-12 exhibits the heat- and solvent- responsive RTP, but without solvent responsive selective reflection. The main role of solvent molecules may reduce the amounts of O2 in free space and increase the passage of the intersystem crossing (ISC), further facilitate the generation of the room temperature phosphorescence.^6-8^


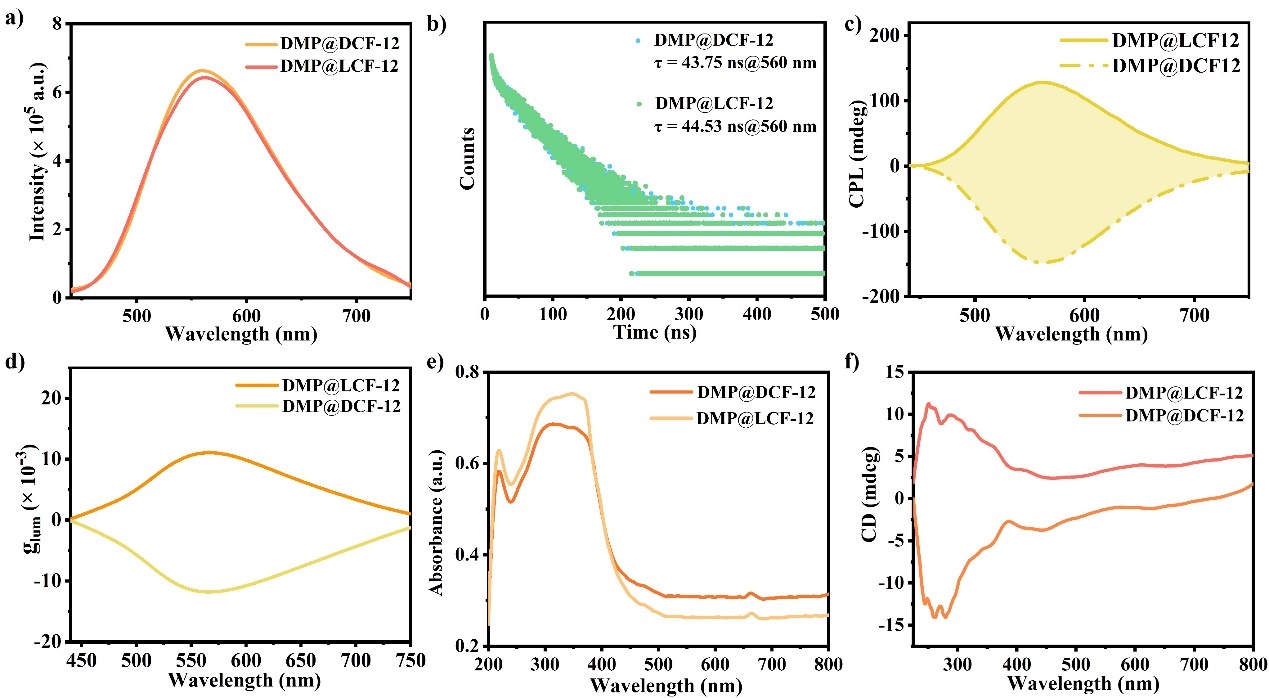


**Figure S30.** (a) The luminescence emission of DMP@DCF-12 and DMP@LCF-12 at excitation wavelengths of 410 nm. (b) The lifetime of DMP@DCF-12 and DMP@LCF-12 at 560 nm. (c) CPL spectra of DMP@DCF-12 and DMP@LCF-12. (d) The *g*_lum_ values of DMP@DCF-12 and DMP@LCF-12. (e) The solid-state absorption of DMP@DCF-12 and DMP@LCF-12. (f) CD spectra of DMP@DCF-12 and DMP@LCF-12 in solid-state.


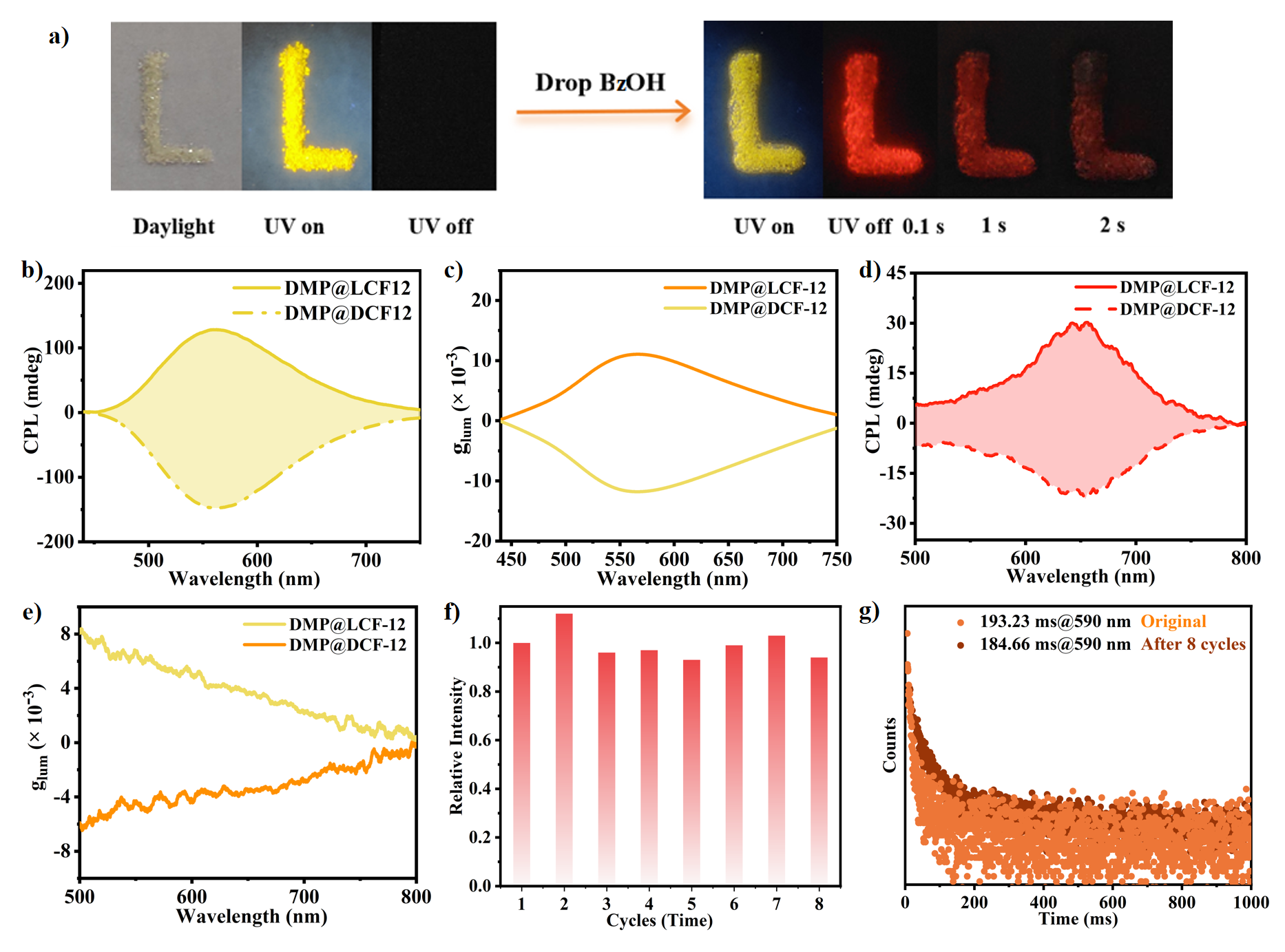


**Figure S31.** (a) The photographs for crystalline samples of DMP@DCF-12 under daylight, 365 nm, and after being dropped BzOH and turning off 365 nm irradiation. (b) CPL spectra of DMP@DCF-12 and DMP@LCF-12 under ambient conditions, when the samples were treated under 120 ℃ for 5 hours. (c) The *g*_lum_ values of DMP@DCF-12 and DMP@LCF-12, when the samples were treated under 120 ℃ for 5 hours. (d) CPL spectra of DMP@DCF-12 and DMP@LCF-12 under ambient conditions, when the samples were dropped by BzOH after 5 hours. (e) The *g*_lum_ values of DMP@DCF-12 and DMP@LCF-12, when the samples were dropped by BzOH after 5 hours. (f) The phosphorescence intensity at 590 nm of DMP@DCF-12 during the 8 cycles under ambient conditions. (g) The phosphorescence lifetimes at 590 nm of DMP@DCF-12 after 8 cycles under ambient conditions.


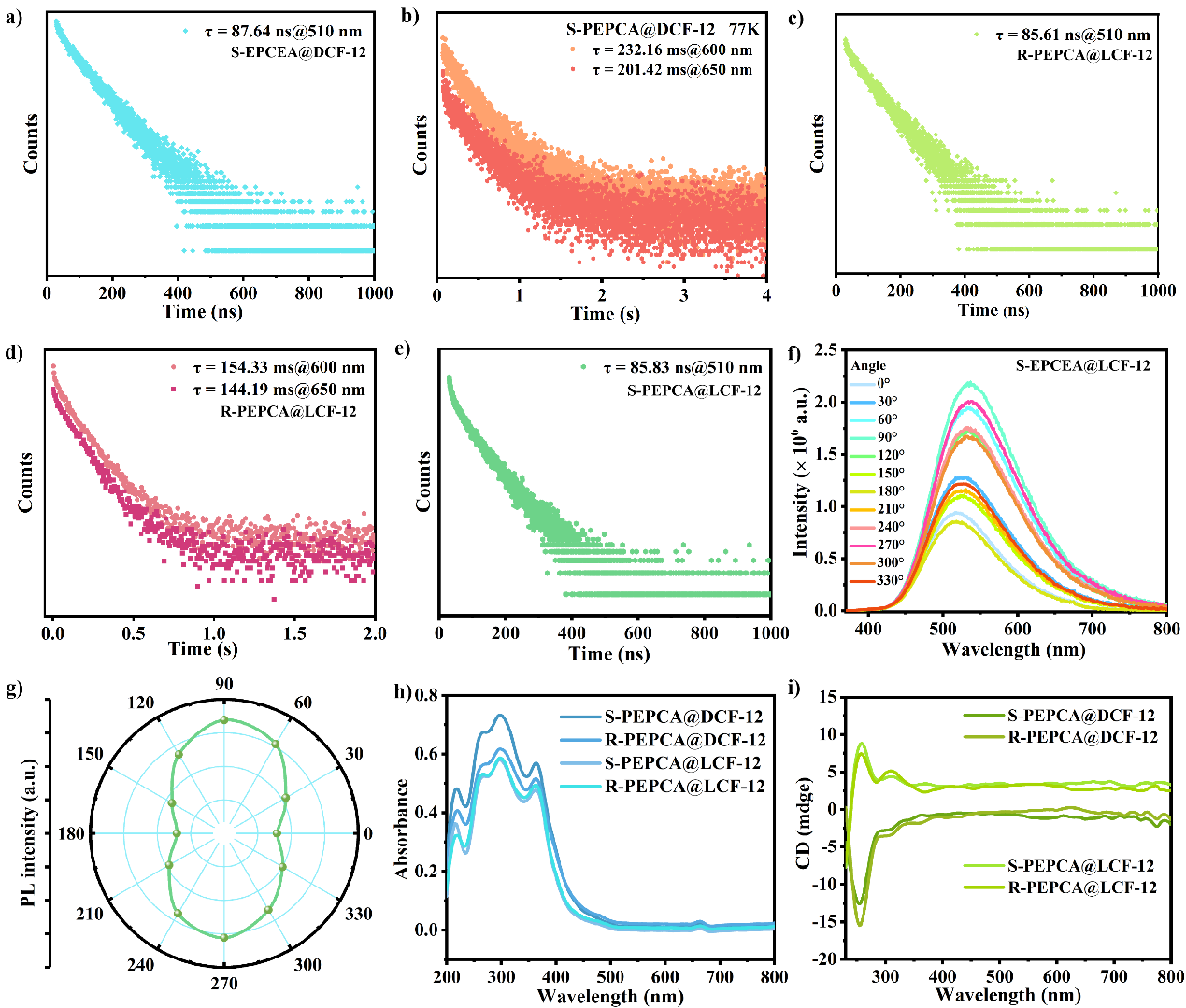


**Figure S32.** (a) The prompt lifetime of S-PEPCA@DCF-12 at 510 nm under room temperatures. (b) Time-resolved decay curves for emission of S-PEPCA@DCF-12 at 600 and 650 nm at 77 K with a delayed time of 0.5 ms. (c) The prompt lifetime of R-PEPCA@LCF-12 at 510 nm under room temperatures. (d) Time-resolved decay curves for emission of R-PEPCA@LCF-12 at 600 and 650 with a delayed time of 0.5 ms under ambient conditions. (e) The prompt lifetime of S-PEPCA@LCF-12 at 510 nm under room temperatures. (f) The maximum polarized emission spectra of S-EPECA@LCF-12 crystal powder at changed angles (0‒360°). (g) The maximum polarized emission spectra of S-PEPCA@LCF-12 crystal powder at changed angles (0‒360°). (h) The solid-state absorption of S-PEPCA@DCF-12, R-PEPCA@DCF-12, S-PEPCA@LCF-12 and R-PEPCA@LCF-12. (i) CD spectra of S-PEPCA@DCF-12, R-PEPCA@DCF-12, S-PEPCA@LCF-12 and R-PEPCA@LCF-12 in solid-state.


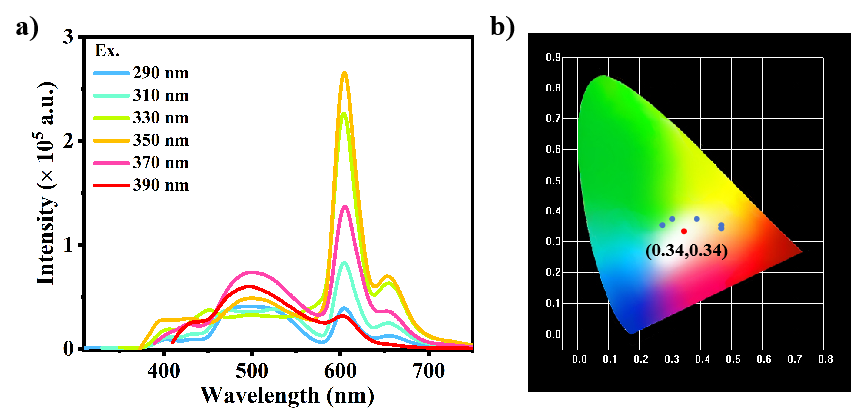


**Figure S33** (a) The photoluminescence spectra of S-PEPCA@DCF-12 under different excitation wavelengths from 290 to 390 nm with a delayed time of 0.5 ms. (b) CIE coordinate diagram of the photoluminescence spectra of S-PEPCA@DCF-12 with the excitation wavelengths changing from 290 to 390 nm with a delayed time of 0.5 ms.

**
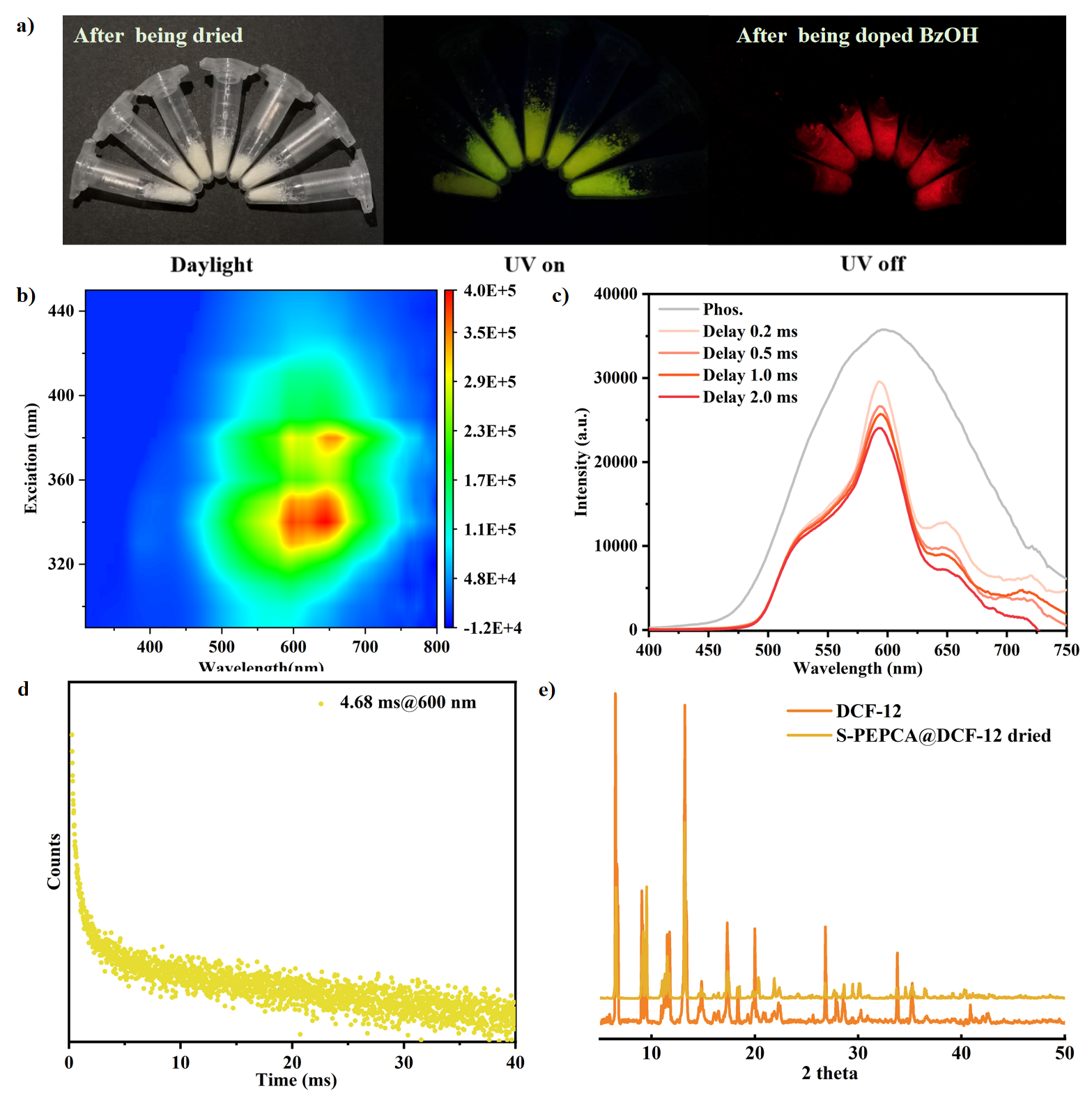
**

**Figure S34** (a) The photographs of fluorescence and afterglow for crystalline samples of S-PEPCA@DCF-12 under daylight, 365 nm, and after dropping BzOH and turning off 365 nm irradiation. (b) Excitation-phosphorescence mapping of the S-PEPCA@DCF-12 after being heated under 120 ℃ for 5 hours with different excitation wavelengths from 280 to 450 nm. (c) The phosphorescence spectra of S-PEPCA@DCF-12 after being heated under 120 ℃ for 5 hours. (d) Time-resolved decay curves for emission at 593 nm at ambient temperature with a delayed time of 0.5 ms. (e) PXRD patterns of S-PEPCA@DCF-12 and the dried S-PEPCA@DCF-12.

DCF-12 crystals were synthesized in a mixed solution of DMA (4 mL), Benzyl alcohol (BzOH, 2 mL) and Deionized water (1 mL), S-PEPCA molecule was introduced into DCF-12 via in-situ method, so the porosity of S-PEPCA@DCF-12 largely accumulates BzOH molecules. In order to confirmed the solvent effect (BzOH) on the phosphorescence behaviors of S-PEPCA@DCF-12, according to the TGA curve of S-PEPCA@DCF-12, S-PEPCA@DCF-12 sample was heated under 120 ℃ for 5 hours, then the photophysical property of S-PEPCA@DCF-12 without BzOH should be measured. As shown in Figure S34, it can find that the phosphorescence emission intensity of the dried S-PEPCA@DCF-12 between 500 to 750 nm become weaker, and the phosphorescence delayed time reduced evidently from 146.93 to 4.68 ms at 600 nm. These results indicate that the phosphorescence emission can not be observed by naked eye. It is noteworthy here that the main phosphorescence emission has remained unchanged with the intensity change, and the sample maintains the high crystallinity and stability after heating treatment.

**
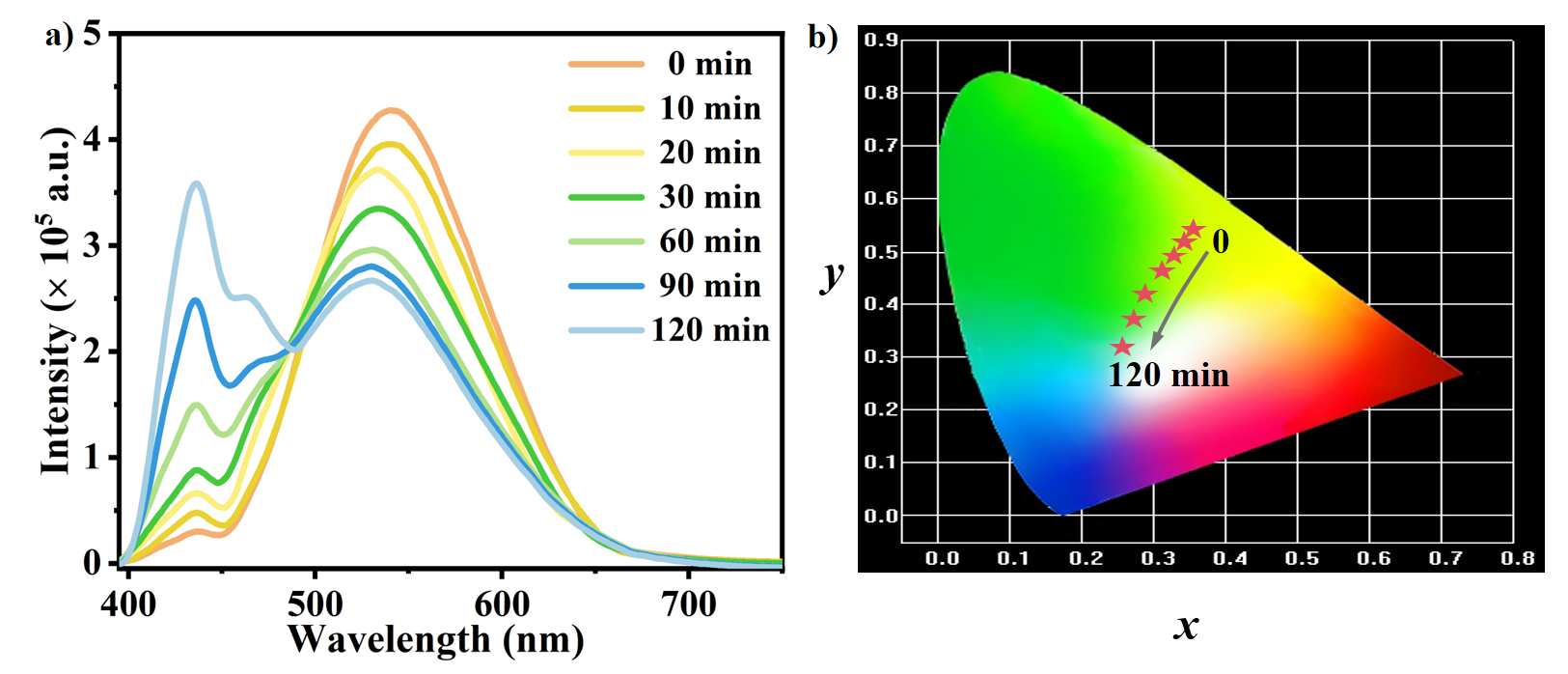
**

**Figure S35.** (a) Emission of EPEA@DCF-12 upon visible light irradiation under different times. (b) The CIE coordinates of DCF-1 and LCF-13.

**
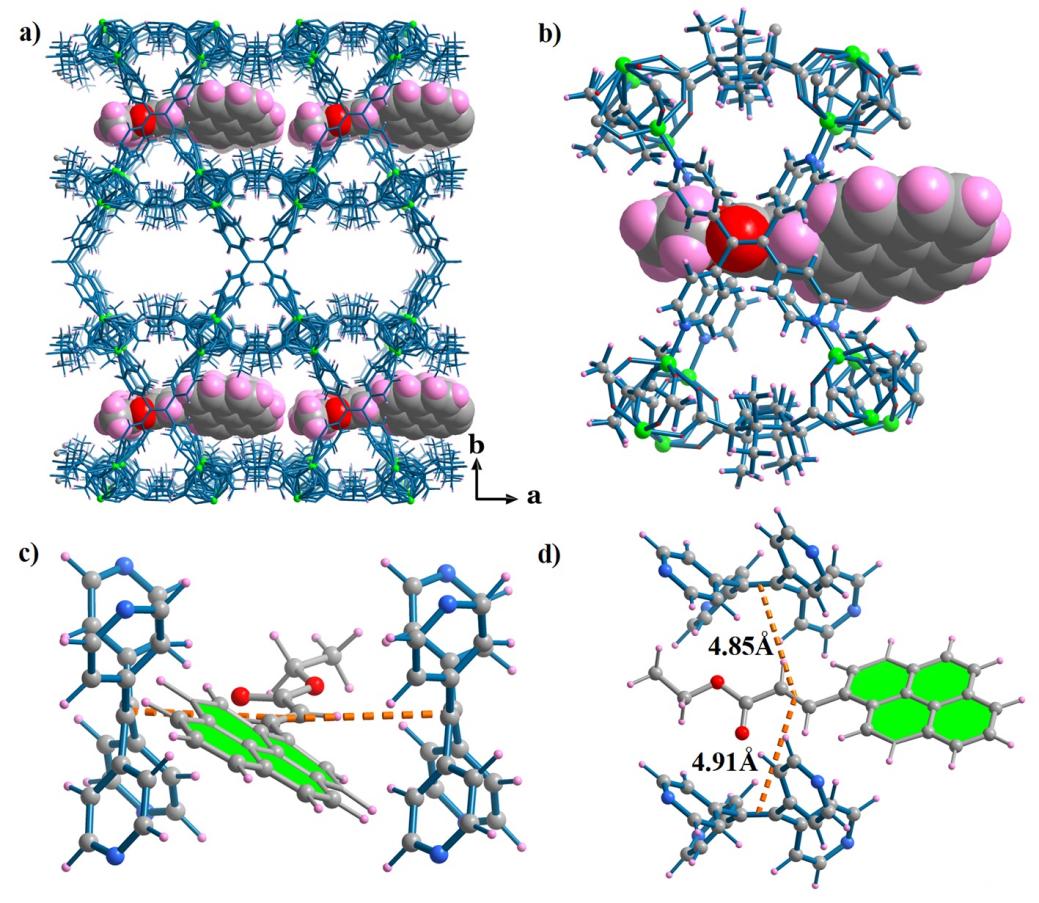
**

**Figure S36.** The EPEA@DCF-12 structures of the simulation results.


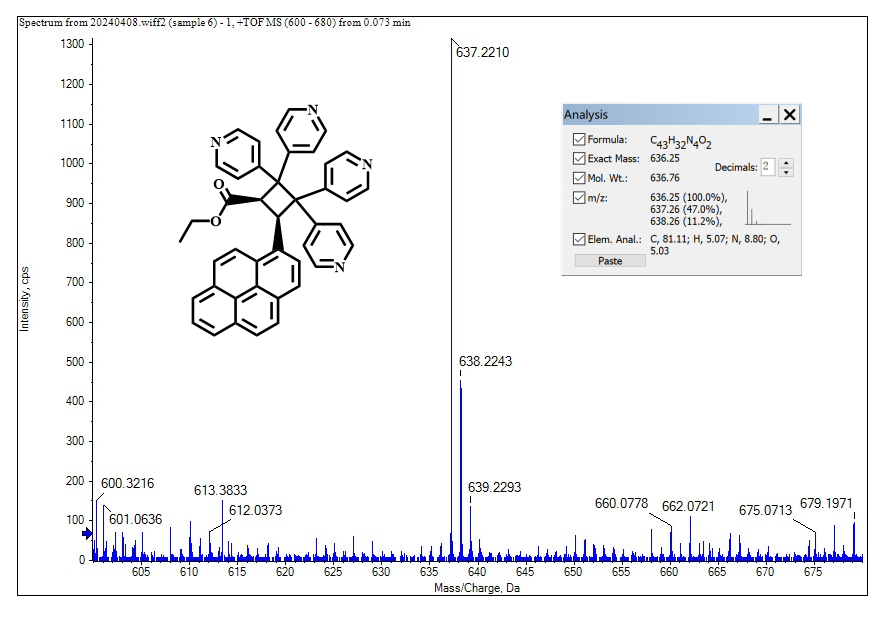


**Figure S37.** The mass spectrum: EPEA@DCF-12 which was exposed in visible for 2 hours, was dissolved in aqueous solution by concentrated hydrochloric acid (37% HCl).

**
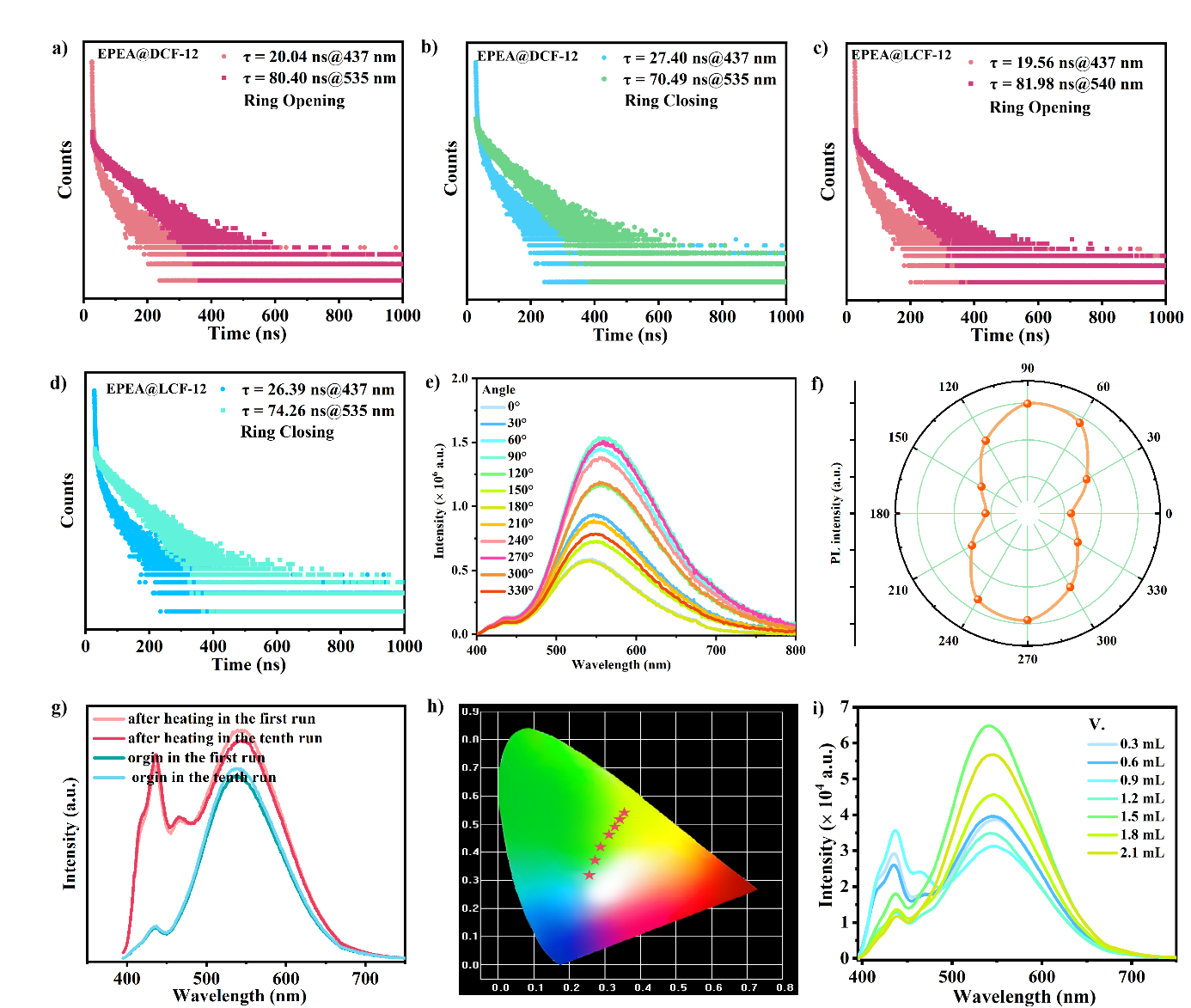
**

**Figure S38.** (a) The prompt lifetime of EPEA@DCF-12 at 437 and 535 nm at room temperature under ring opening state, namely, EPEA@DCF-12 recovered to the initial state through the heating treatment at 100 ℃. (b) The prompt lifetime of EPEA@DCF-12 at 437 and 535 nm under heating treatment at 100 ℃. (c) The prompt lifetime of EPEA@LCF-12 at 437 and 535 nm at room temperature under ring opening state, namely, EPEA@LCF-12 recovered to the initial state through the heating treatment at 100 ℃. (d) The prompt lifetime of EPEA@LCF-12 at 437 and 535 nm under heating treatment at 100 ℃. (e) The emission intensity of EPEA@LCF-12 crystal powder at changed angles (0‒360°). (f) The maximum polarized emission spectra of EPEA@LCF-12 crystal powder at changed angles (0‒360°). (g) The [2+2] cycloaddition reaction between EPEA and TPE shows the excellent reversibility. The emission behaviors at 437 and 535 nm show the good recoverability after 10 times. (h) CIE coordinate diagram of the luminescence spectra of EPEA@DCF-12 upon visible light irradiation under different times (0‒120 min). (i) Luminescence emission of EPEA@DCF-12 encapsulated with different volumes of EPEA solution (1 × 10^‒2^ mol/L in DMA).


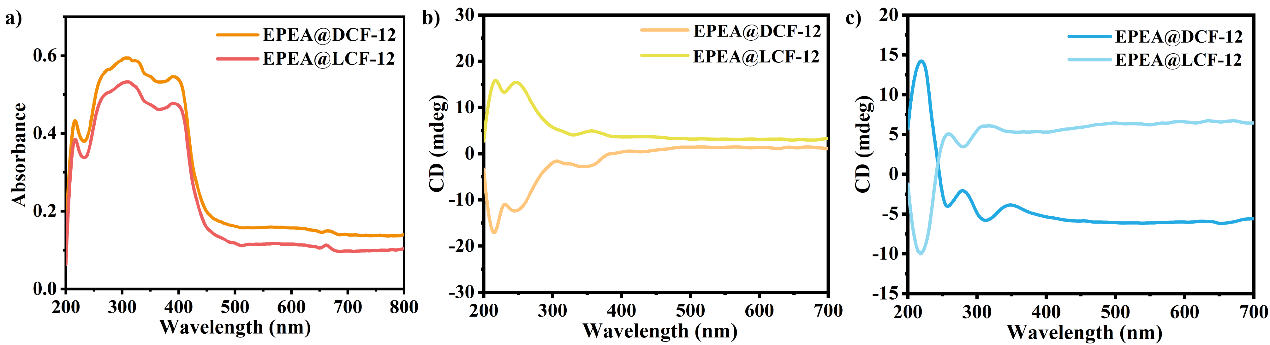


**Figure S39.** (a) The solid-state absorption of EPEA@DCF-12 and EPEA@LCF-12. (b) CD spectra of EPEA@DCF-12 and EPEA@LCF-12 under ring opening state. (c) CD spectra of EPEA@DCF-12 and EPEA@LCF-12 under ring closing state.

**
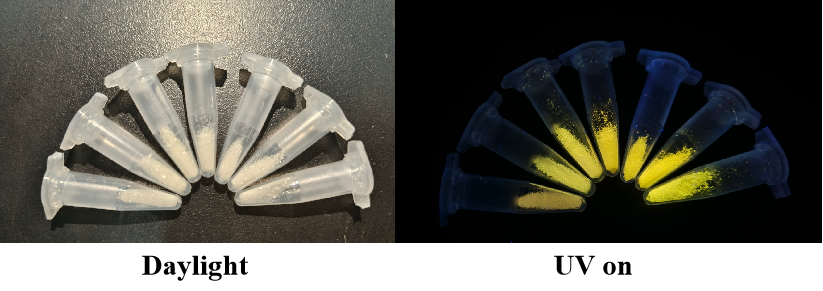
**

**Figure S40.** The photographs of EPEA@DCF-12 under daylight and UV.

**
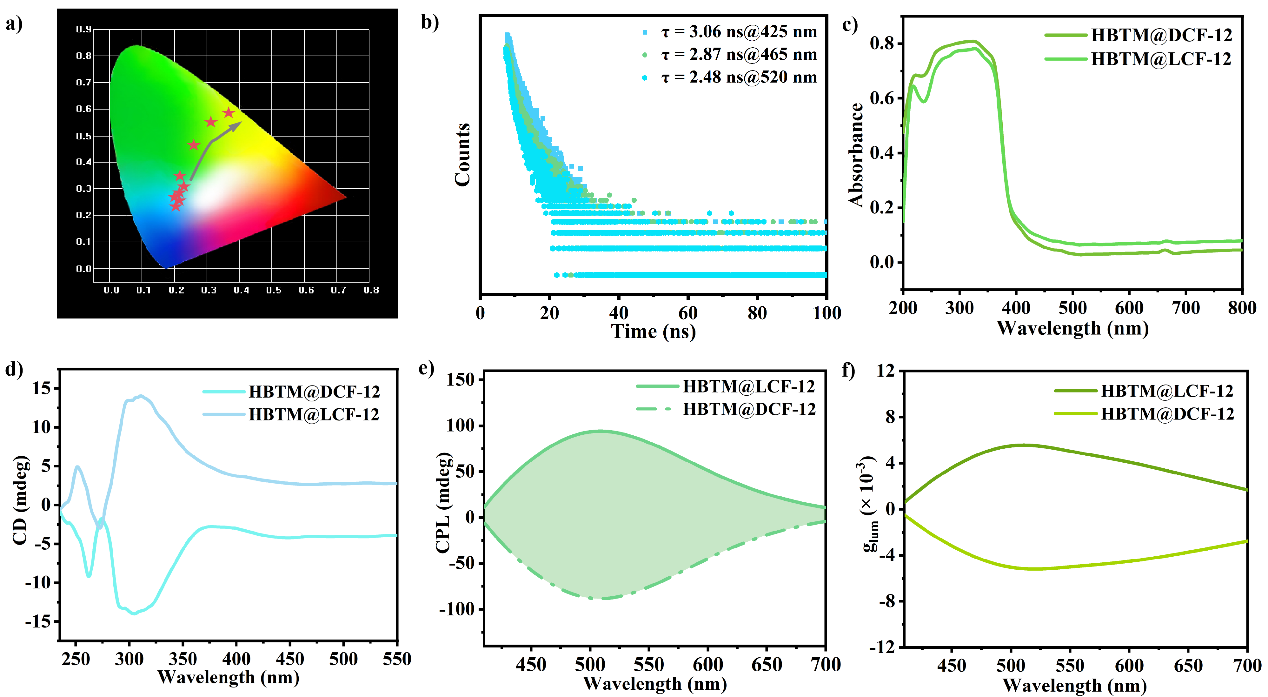
**

**Figure S41.** (a) CIE coordinate diagram of the luminescence spectra of HBTM@DCF-12 with the excitation wavelengths changing from 305 to 465 nm. (b) The prompt lifetime of HBTM@DCF-12 at 425 465 and 520 nm at room temperature. (c) The solid-state absorption of HBTM@DCF-12 and HBTM@LCF-12. (d) CD spectra of HBTM@DCF-12 and HBTM@LCF-12. (e) CPL spectra of HBTM@DCF-12 and HBTM@LCF-12. (f) The *g*_lum_ values of HBTM@DCF-12 and HBTM@LCF-12.

**
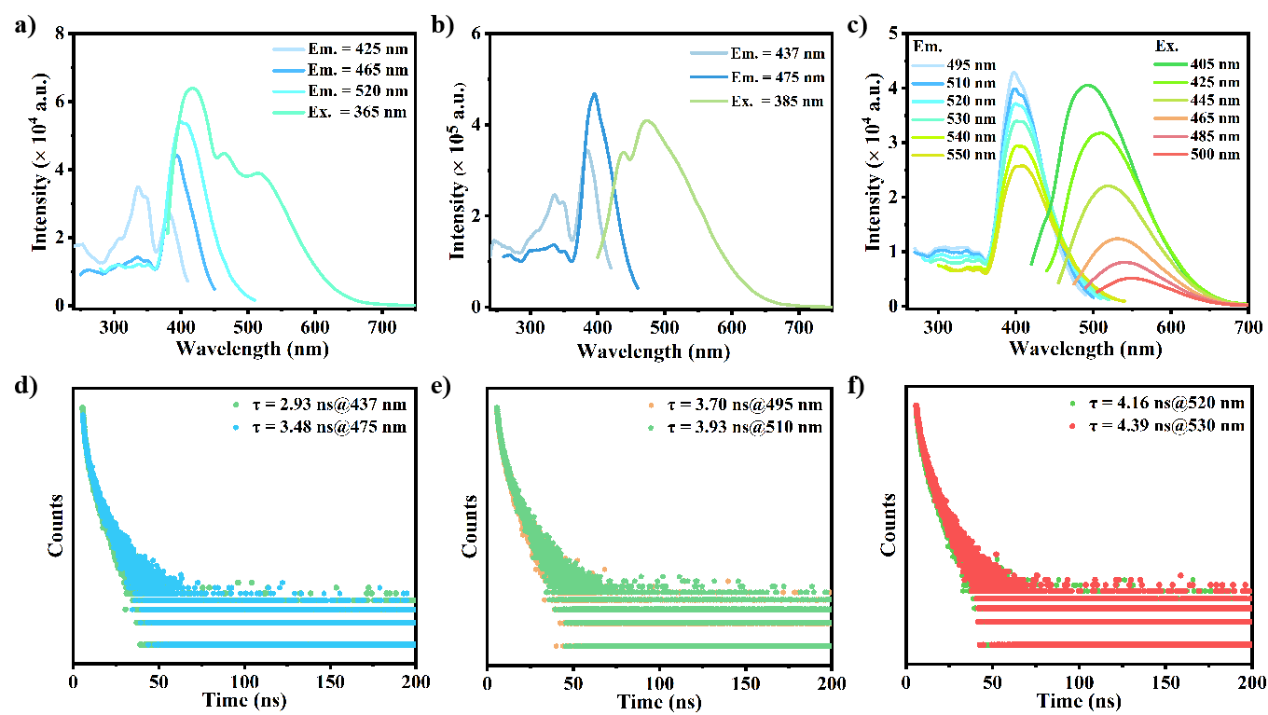
**

**Figure S42.** (a) Emission and excitation spectra of HBTM@DCF-12 at 365 nm. (b) Emission and excitation spectra of HBTM@DCF-12 at 385 nm. (c) Emission and excitation spectra of HBTM@DCF-12 under different excitation wavelengths from 405 to 500 nm. (d) The prompt lifetime of HBTM@DCF-12 at 437 and 475 nm at room temperature. (e) The prompt lifetime of HBTM@DCF-12 at 495 and 510 nm at room temperature. (f) The prompt lifetime of HBTM@DCF-12 at 520 and 530 nm at room temperature.

**Table S1.** Emission peaks and their lifetimes of HBTM@DCF-12 at different excitation wavelengths.

| **Ex. (nm)** | **Em. (nm)** | **τ (ns)** |
| --- | --- | --- |
| 365 | 425、465、520 | 3.06、2.87、2.48 |
| 385 | 437、475 | 2.93、3.48 |
| 405 | 495 | 3.70 |
| 425 | 510 | 3.93 |
| 445 | 520 | 4.16 |
| 465 | 530 | 4.39 |

**
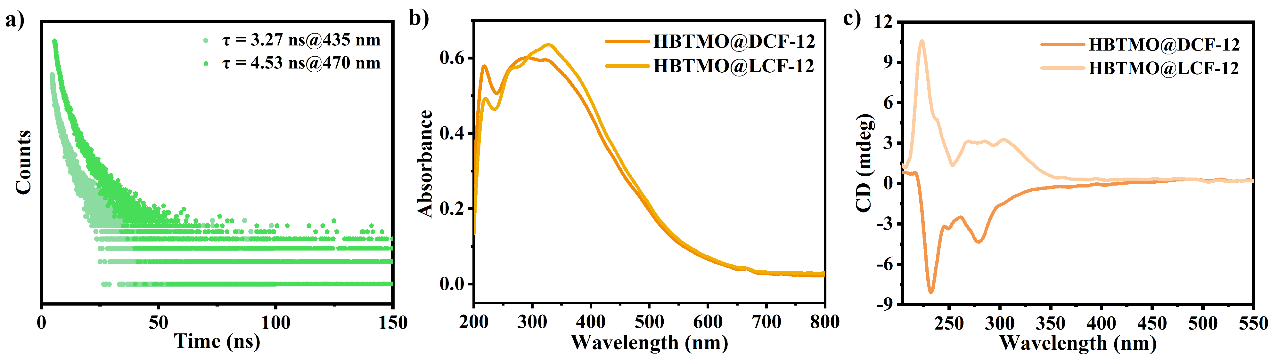
**

**Figure S43.** (a) The prompt lifetime of HBTMO@DCF-12 at 435 and 470 nm at room temperature. (b) The solid-state absorption of HBTMO@DCF-12 and HBTMO@LCF-12. (c) CD spectra of HBTMO@DCF-12 and HBTMO@LCF-12.

**
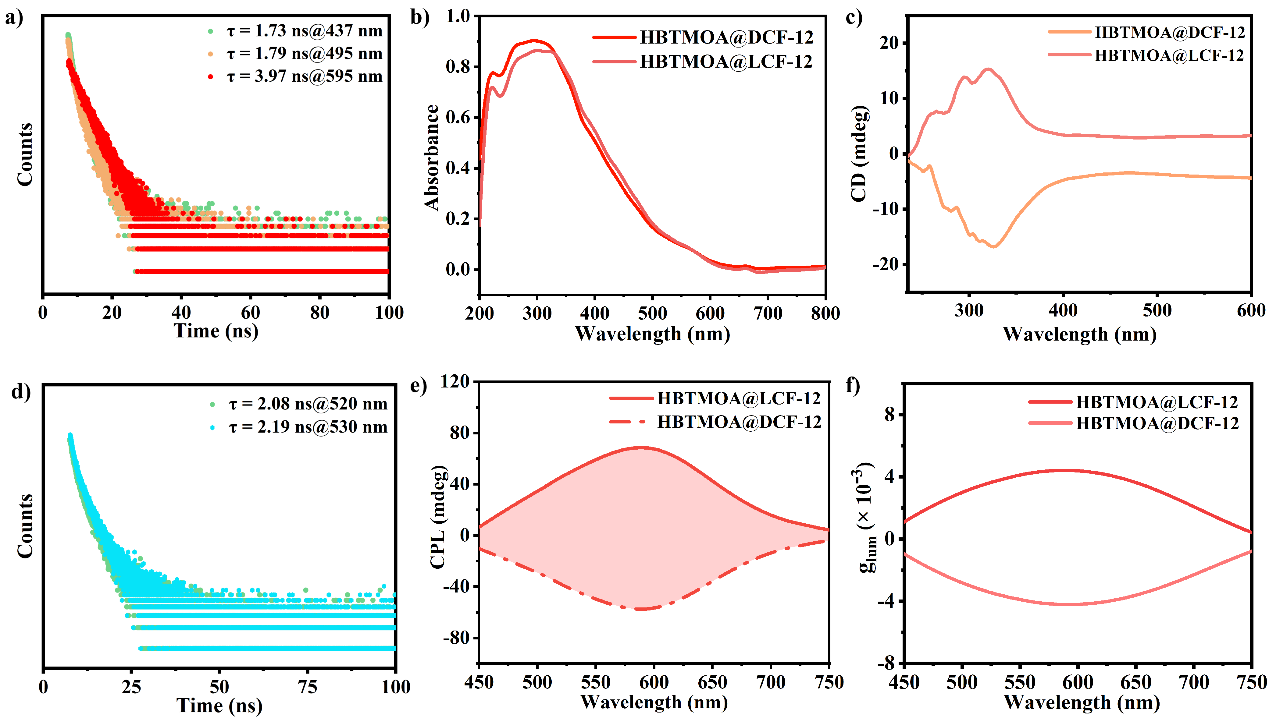
**

**Figure S44.** (a) The prompt lifetime of HBTMOA@DCF-12 at 437 495 and 595 nm at room temperature. (b) The solid-state absorption of HBTMOA@DCF-12 and HBTMOA@LCF-12. (c) CD spectra of HBTMOA@DCF-12 and HBTMOA@LCF-12. (d) The prompt lifetime of HBTMOA@DCF-12 at 520 and 530 nm at room temperature. (e) CPL spectra of HBTMOA@DCF-12 and HBTMOA@LCF-12. (f) The *g*_lum_ values of HBTMOA@DCF-12 and HBTMOA@LCF-12.

The measurement of the phosphorescent quantum yields: The quantum yields (QYs) performed on FLS1000 Edinburgh photoluminescence spectrometer (under the optimal excitation wavelengths based on the spectra measurement). The phosphorescence QYs was calculated using the following equation by reference to the literatures.^9,10^ As follows:

$$\emptyset_{Phos}= \emptyset_{PL}* \frac{A_{Phos}}{A_{FL}+ A_{Phos}}$$

Herein, Ф_Phos_ is the phosphorescence QY, Ф_PL_ is the PL QY; A_FL_ and A_Phos_ are integral peak areas of fluorescence FL and phosphorescence.

**
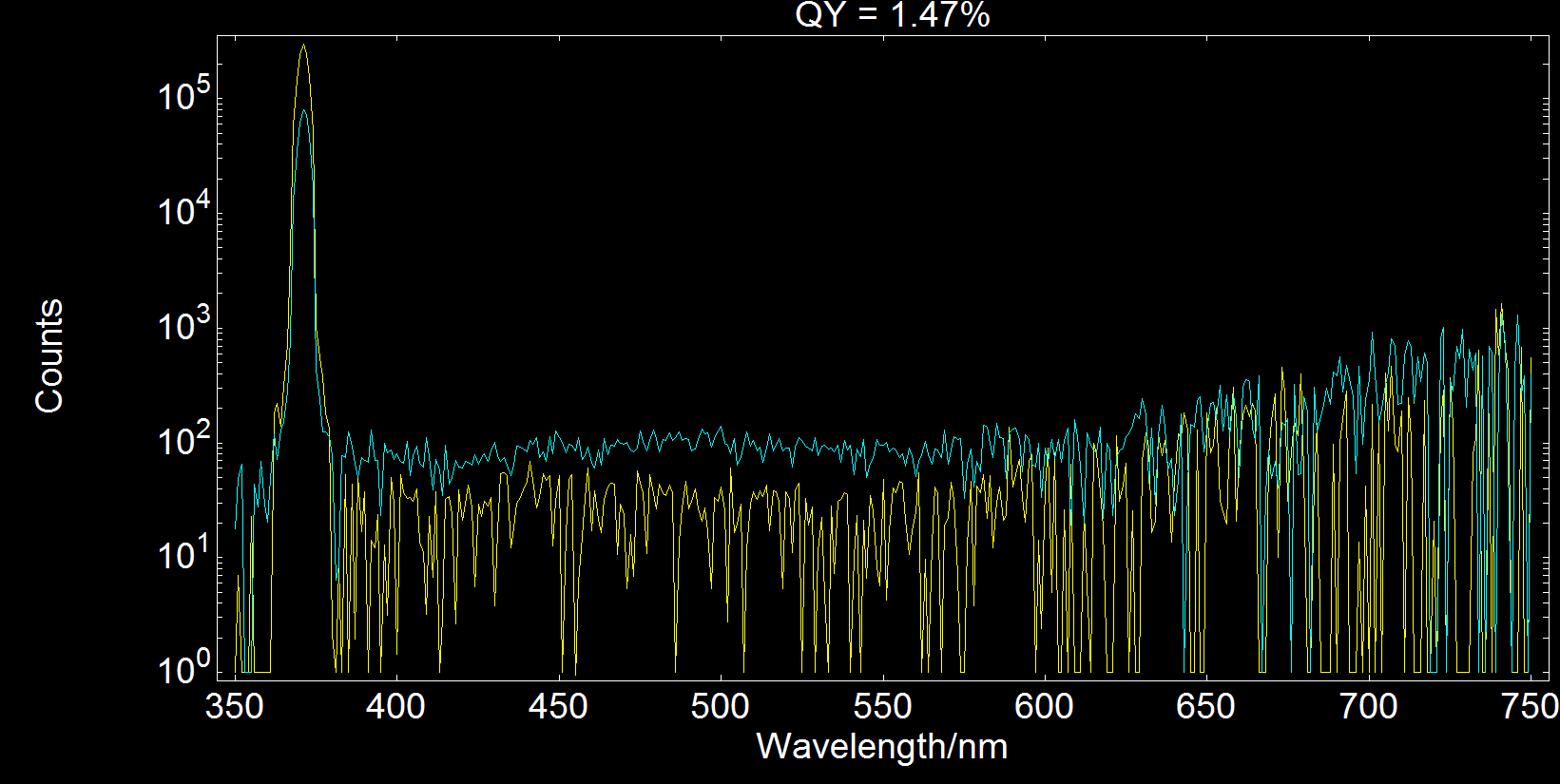
**

**Figure S45.** Fluorescence quantum yield of DCF-12 under ambient conditions.


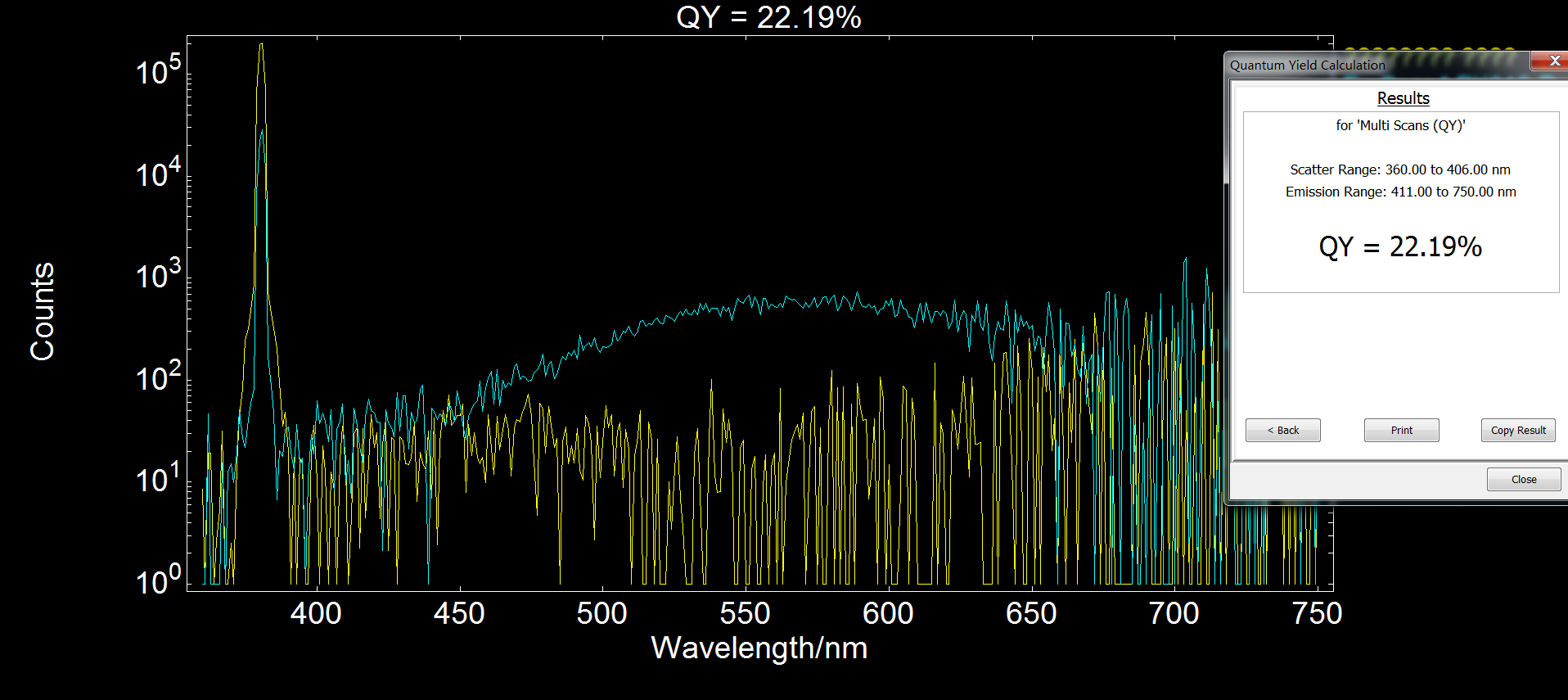


**Figure S46.** Fluorescence quantum yield of DMP@DCF-12 under ambient conditions.

**
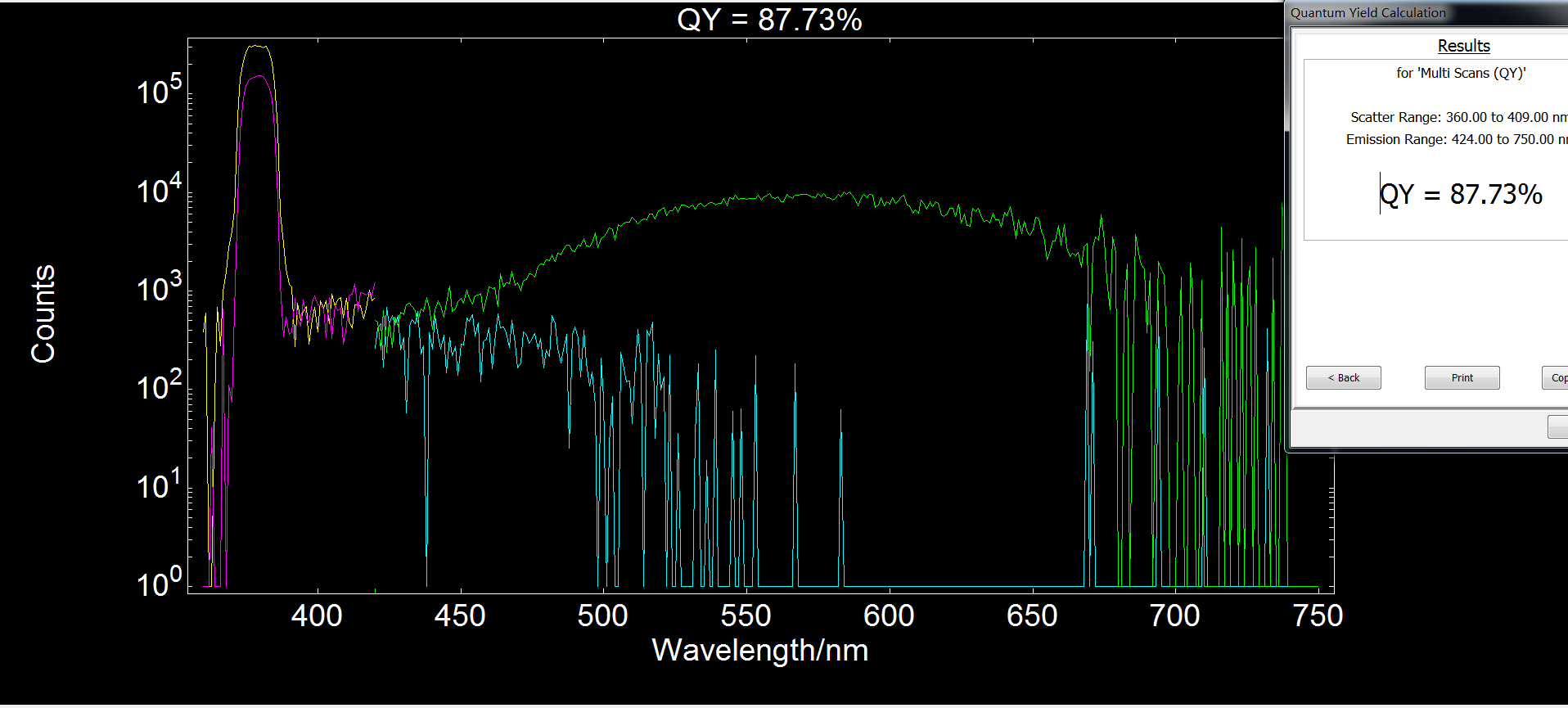
**

**Figure S47.** Phosphorescence quantum yield of DMP@DCF-12 under ambient conditions.


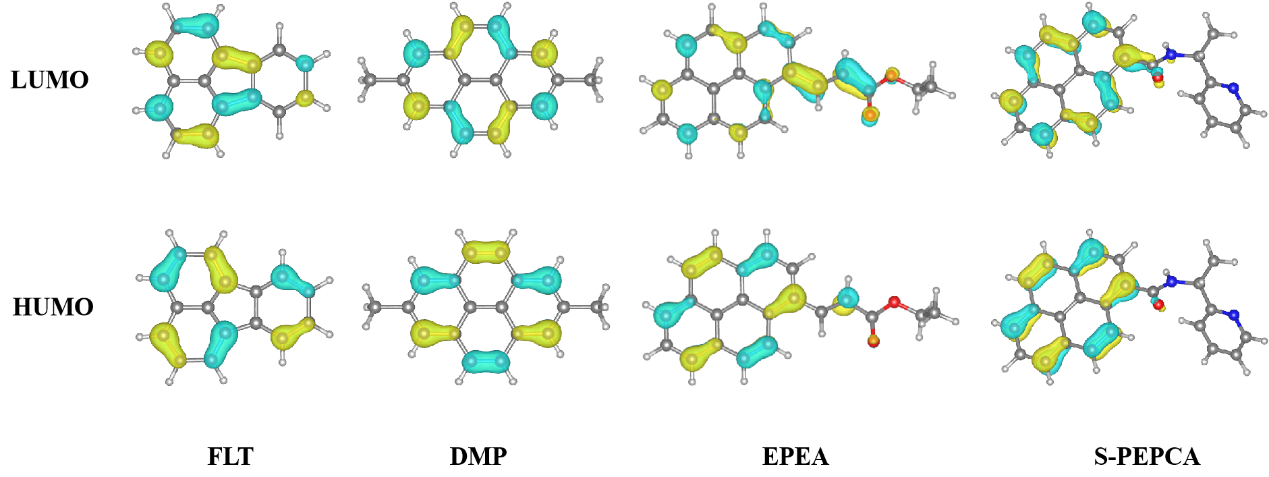


**Figure S48.** DFT-calculated HOMO and LUMO of FLT, DMP, EPEA and S-PEPCA.


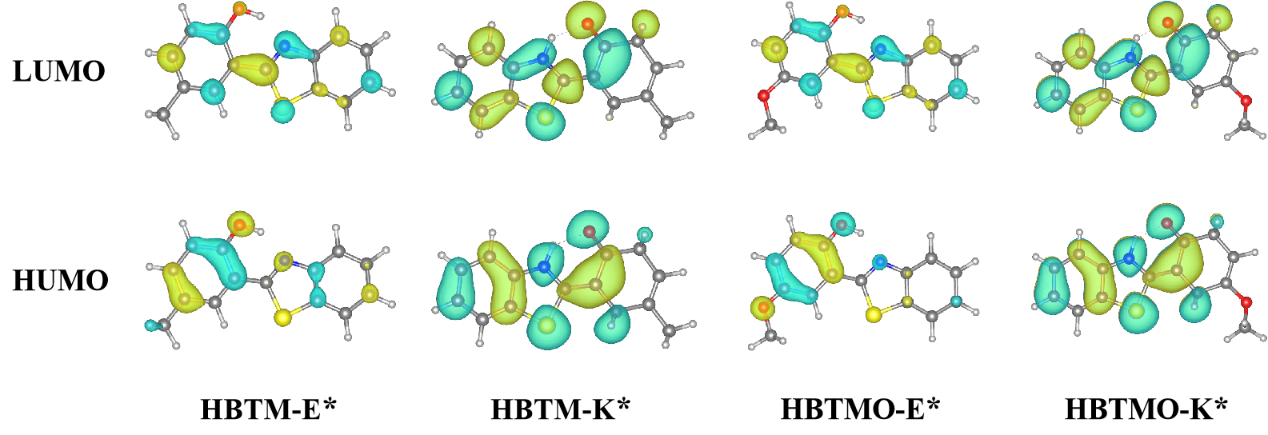


**Figure S49.** DFT-calculated HOMO and LUMO of HBTM-E*, HBTM-K*, HBTMO-E* and HBTMO-K*.

**
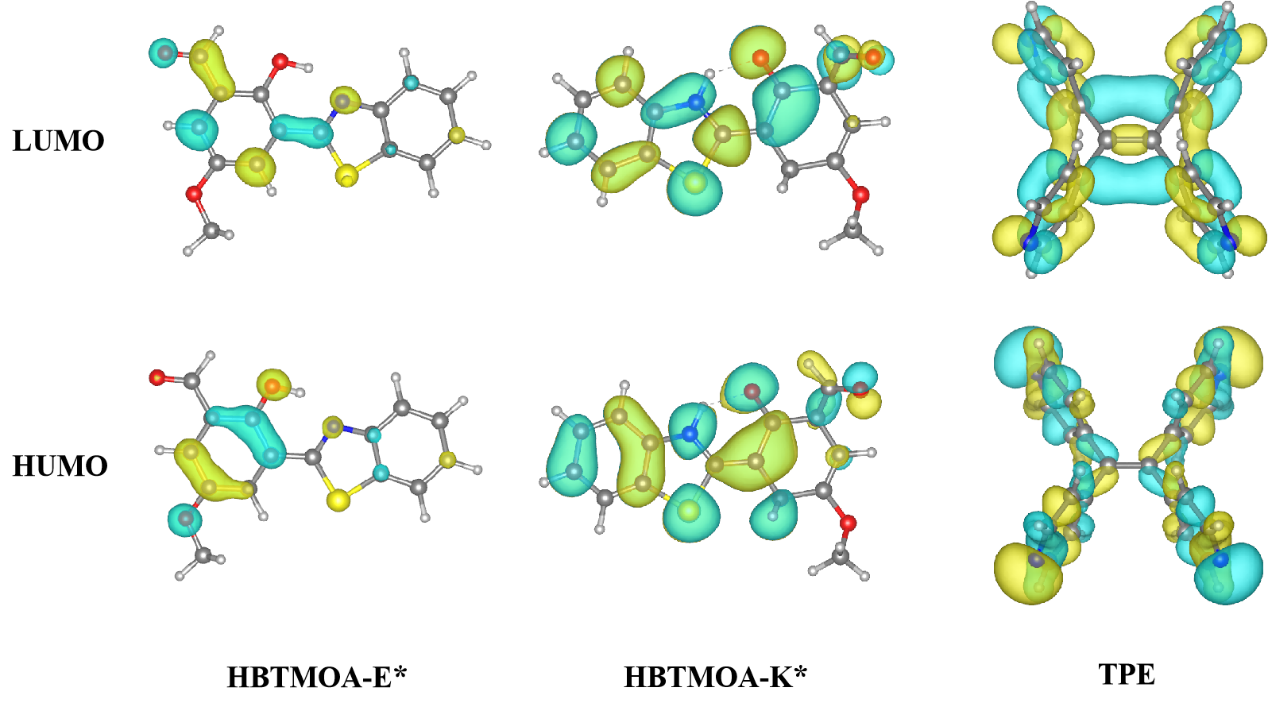
**

**Figure S50.** DFT-calculated HOMO and LUMO of HBTMOA-E*, HBTMOA-K* and TPE.


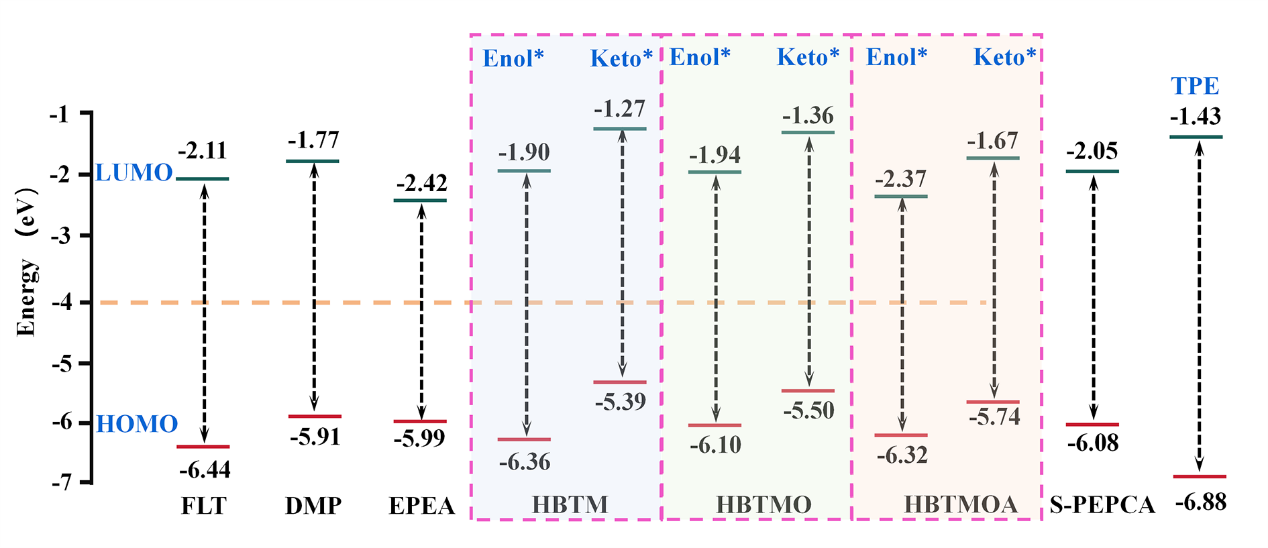


**Figure S51.** The energy levels of TPE and the guest emitters.

| **Table S2. The crystallographic parameters of FLT@DCF-12 and FLT@LCF-12** | | |
| --- | --- | --- |
| Identification code | FLT@DCF-12 | FLT@LCF-12 |
| Number of CCDC | 2391055 | 2391056 |
| Empirical formula | C_280_H_309_N_16_O_64.50_Zn_16_ | C_280_H_310_N_16_O_65_Zn_16_ |
| Formula weight | 5976.33 | 5985.34 |
| Temperature/K | 100.01(10) | 100.01(10) |
| Crystal system | orthorhombic | orthorhombic |
| Space group | *I*222 | *I*222 |
| a / Å | 18.2671(2) | 18.2866(6) |
| b / Å | 26.4467(3) | 19.1656(5) |
| c / Å | 19.1719(2) | 26.4464(9) |
| **α** / ° | 90 | 90 |
| **β** / ° | 90 | 90 |
| **γ** / ° | 90 | 90 |
| Volume / Å^3^ | 9262.03(18) | 9268.8(5) |
| Z | 8 | 8 |
| Ρcalcg/cm^3^ | 1.071 | 1.072 |
| μ / mm^-1^ | 1.075 | 1.075 |
| F(000) | 3097.0 | 3102.0 |
| Crystal size / mm^3^ | 0.13 × 0.12 × 0.1 | 0.13 × 0.12 × 0.1 |
| Radiation | Mo K**α** (**λ** = 0.71073) | Mo K**α** (**λ** = 0.71073) |
| 2θ range for data collection/° | 6.754 to 58.322 | 6.756 to 58.278 |
| Index ranges | -13 ≤ h ≤ 24, -33 ≤ k ≤ 34, -14 ≤ l ≤ 25 | -18 ≤ h ≤ 25, -25 ≤ k ≤ 23, -17 ≤ l ≤ 35 |
| Reflections collected | 14642 | 15461 |
| Independent reflections | 10085 [R_int_ =0.0152, R_sigma_  = 0.0371] | 9984 [R_int_ = 0.0183, R_sigma_  = 0.0429] |
| Goodness-of-fit on F^2^ | 1.035 | 1.060 |
| Flcak | 0.087(14) | -0.02(3) |
| R_1_ [I>2σ (I)] | R_1_ = 0.0347, wR_2_ = 0.0914 | R_1_ = 0.0614, *w*R_2_ = 0.1938 |
| wR_2_ (all data) | R_1_ = 0.0410, *w*R_2_ = 0.0958 | R_1_ = 0.0731, *w*R_2_ = 0.2074 |

**Table S3.** The yields of DCF-12/LCF-12 and guest-encapsulated MOFs.

| Name | Yield (%) | Name | Yield (%) |
| --- | --- | --- | --- |
| DCF-12 | ~65.6% | LCF-12 | ~64.3% |
| FLT@DCF-12 | ~63.3% | FLT@LCF-12 | ~64.2% |
| DMP@DCF-12 | ~62.3% | DMP@LCF-12 | ~63.1% |
| *R*-PEPCA@DCF-12 | ~61.8% | *R*-PEPCA@LCF-12 | ~61.5% |
| *S*-PEPCA@DCF-12 | ~61.3% | *S*-PEPCA@LCF-12 | ~60.4% |
| EPEA@DCF-12 | ~62.8% | EPEA@LCF-12 | ~61.9% |
| HBTM@DCF-12 | ~63.9% | HBTM@LCF-12 | ~63.4% |
| HBTMO@DCF-12 | ~64.2% | HBTMO@LCF-12 | ~62.7% |
| HBTMOA@DCF-12 | ~62.7% | HBTMOA@LCF-12 | ~64.1% |

**
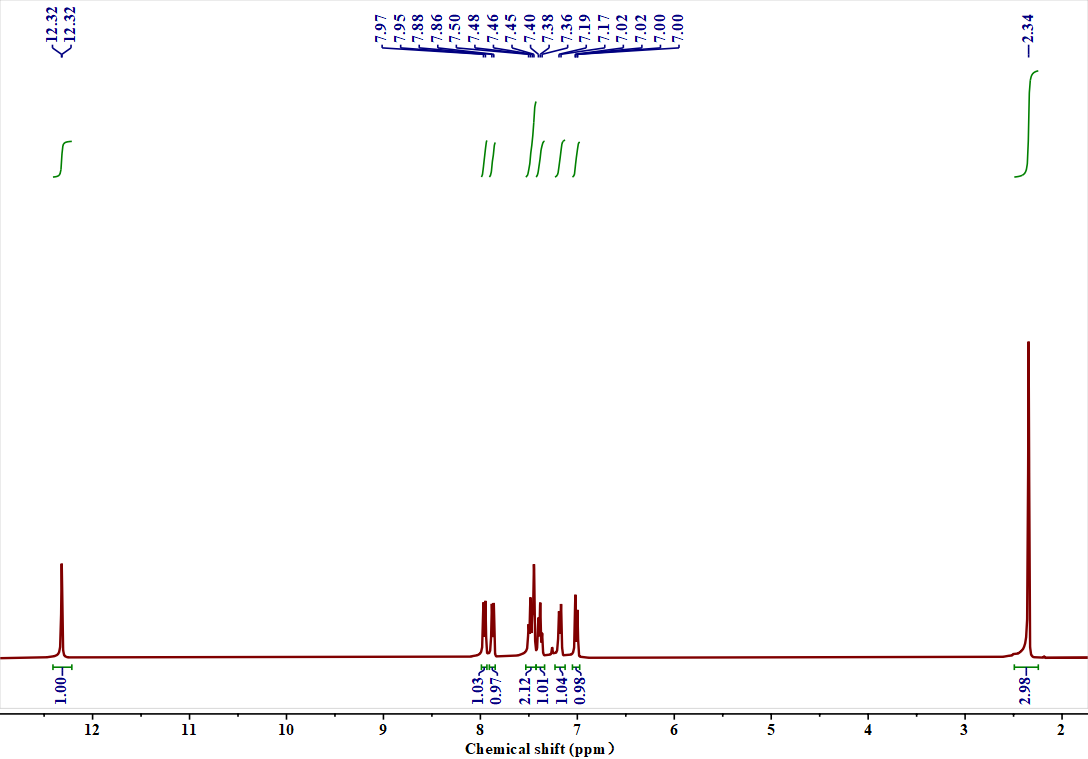
**

**Figure S52.** ^1^HNMR (400 MHz, CDCl_3_) spectrum of HBTM.


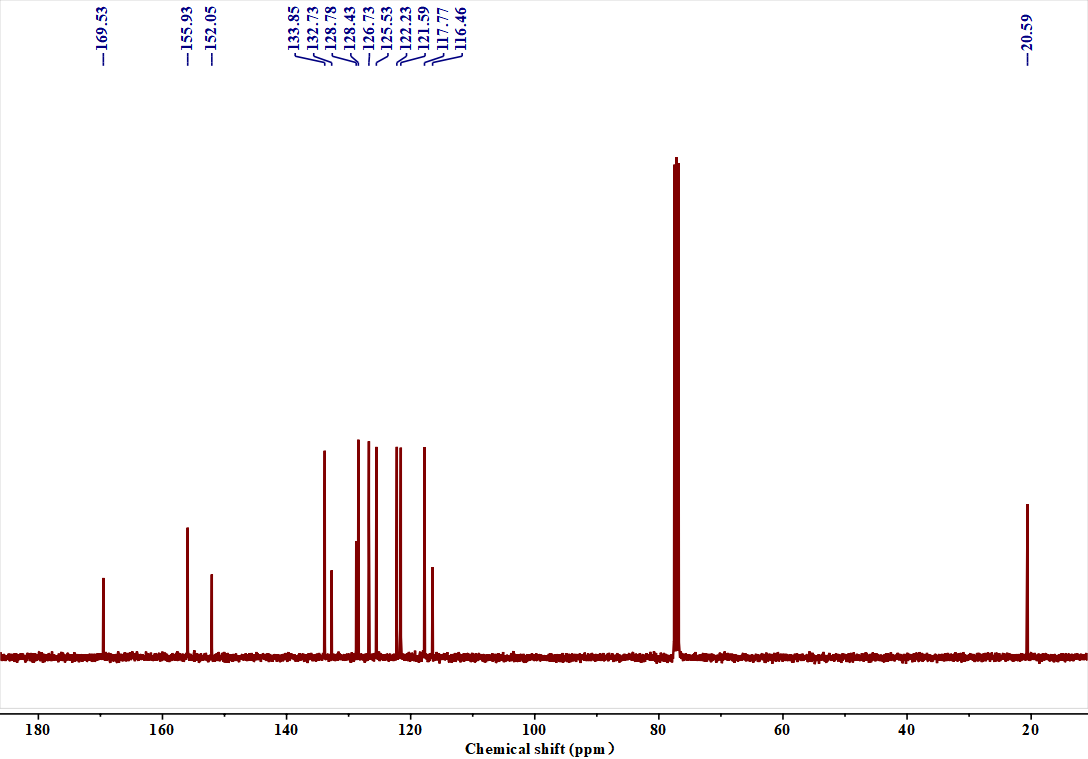


**Figure S53.** ^13^CNMR (100 MHz, CDCl_3_) spectrum of HBTM.

**
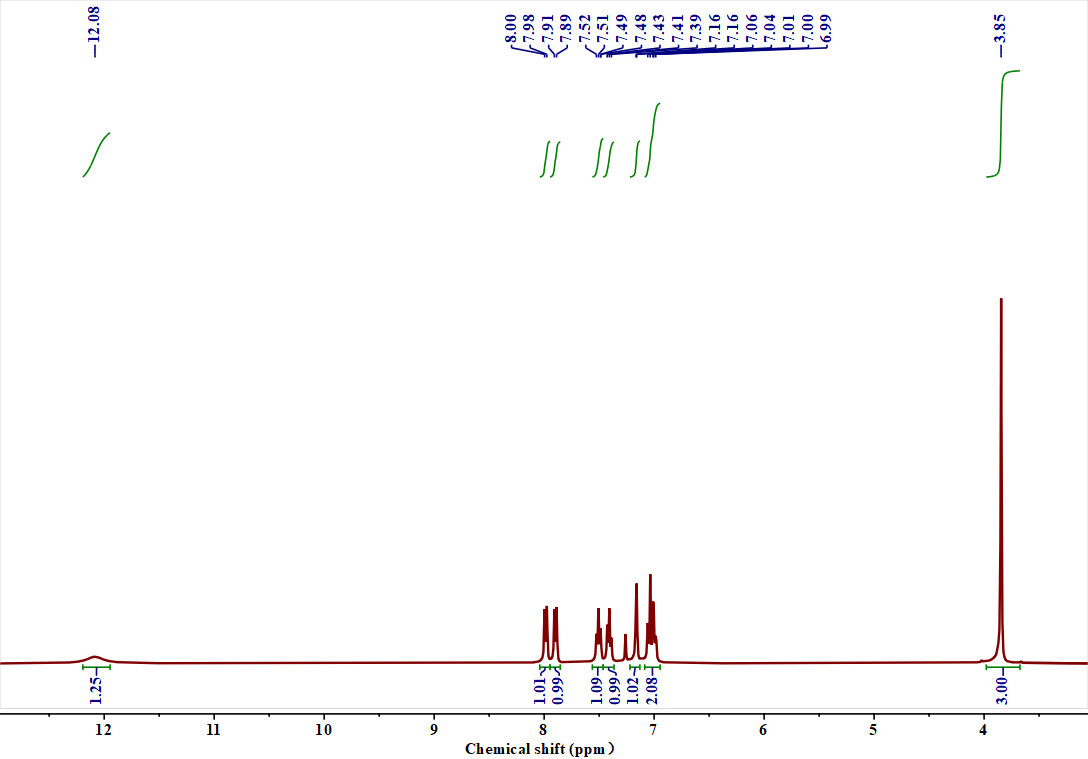
**

**Figure S54.** ^1^HNMR (400 MHz, CDCl_3_) spectrum of HBTMO.


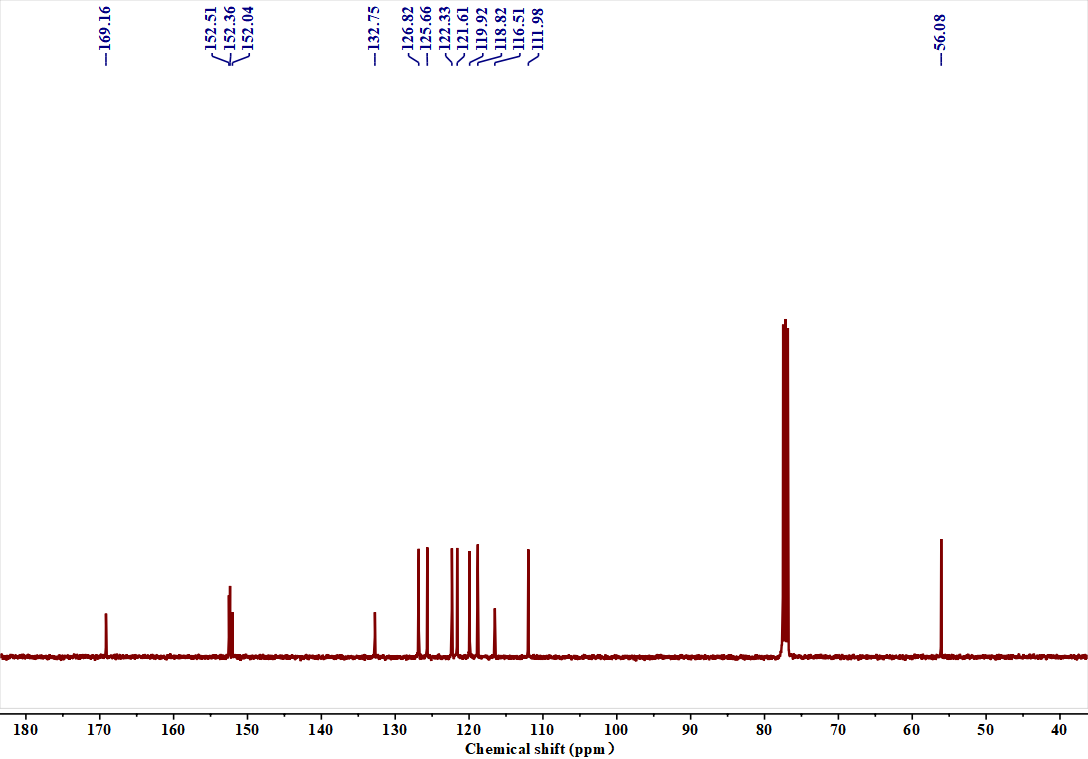


**Figure S55.** ^13^CNMR (100 MHz, CDCl_3_) spectrum of HBTMO.

**
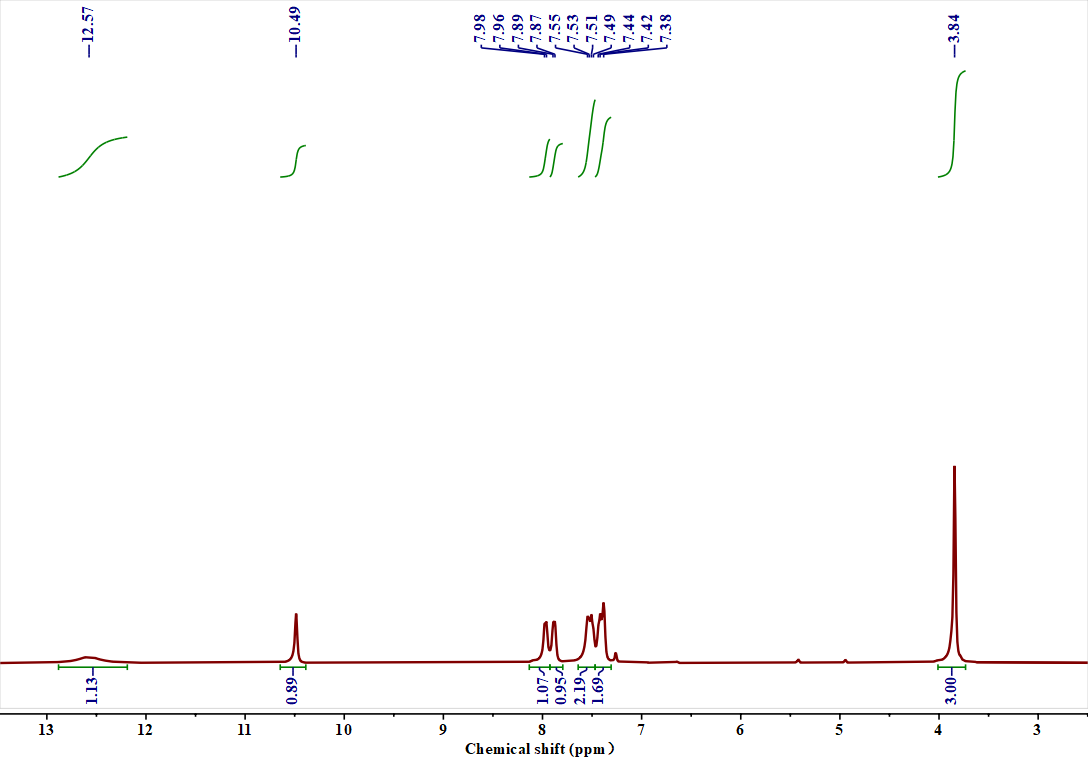
**

**Figure S56.** ^1^HNMR (400 MHz, CDCl_3_) spectrum of HBTMOA.


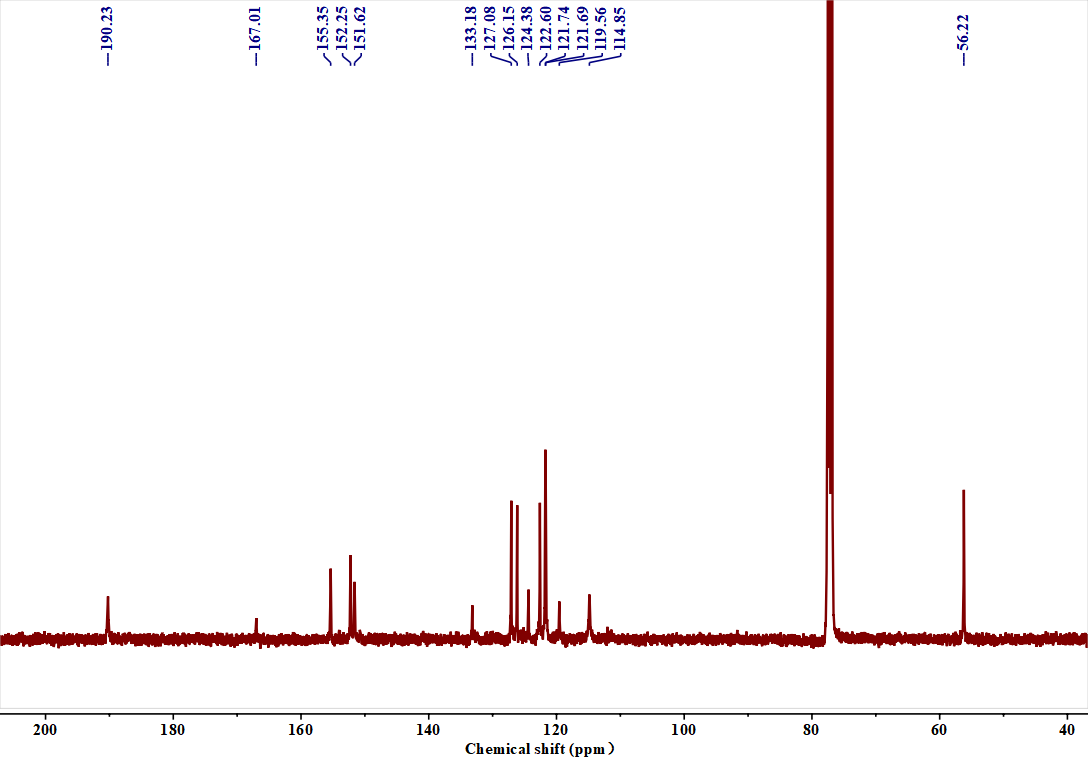


**Figure S57.** ^13^CNMR (100 MHz, CDCl_3_) spectrum of HBTMOA.

**References**

1 W. Feng, D. Chen, Y. Zhao, B. Mu, H. Yan, M. Barboiu, *J. Am. Chem. Soc.,* 2024, *146*, 2484–2493.

2 F. Xiao, H. Gao, Y. Lei, W. Dai, M. Liu, X. Zheng, Z. Cai, X. Huang, H. Wu, D. Ding, *Nat. Commun.,* 2022, *13*, 186.

3 Y. Jiang, C. Zhang, R. Wang, Y. Lei, W. Dai, M. Liu, H. Wu, Y. Tao, X. Huang, *Adv. Optical Mater.*, 2024, 12, 2302482.

4 A. Abe, K. Goushi, M. Mamada, C. Adachi, *Adv. Mater.*, 2024, *36*, 202211160.

5 Q. Chen, L. Qu, H. Hou, J. Huang, C. Li, Y. Zhu, Y. Wang, X. Chen, Q. Zhou, Y. Yang, C. Yang, *Nat Commun*, 2024, *15*, 2947.

6 A. M. Raj, G. Sharma, R. Prabhakar, V. Ramamurthy, *J. Phys. Chem. A,* 2019, *123*, 9123–9131.

7 S. K. Rajagopal, A. R. Mallia, M. Hariharan, *Phys. Chem. Chem. Phys.*, 2017, *19*, 28225–28231.

8 Y. Huang, X. Zheng, Z. Yao, W. Lv, S. Xiang, Q. Ling, Z. Lin. *Chem. Eng. J.*, 2022, *444*, 136629.

9 S. Kim, S. J. Yoon, S. Y. Park, *J. Am. Chem. Soc.* **2012**, *134*, 12091–12097.

10 W. Cao, Y. Tang, Y. Cui, G. Qian, *Small Struct.* **2020**, *1*, 2000019.
